# Automated Dynamic Flow Experimentation for Rapid Kinetic Fitting of Transition Metal Catalysis

**DOI:** 10.1002/anie.6944441

**Published:** 2026-05-15

**Authors:** Florian L. Wagner, Klara Silber, Tobias A. Doliner, C. Oliver Kappe

**Affiliations:** ^1^ Institute of Chemistry University of Graz Graz, Heinrichstrasse 28 Austria; ^2^ Center For Continuous Flow Synthesis and Processing (CCFLOW) Research Center Pharmaceutical Engineering GmbH (RCPE) Graz Austria; ^3^ Institute of Chemistry University of Graz Graz, Schubertstraße 1 Austria

**Keywords:** buchwald‐hartwig amination, copper catalysis, data‐rich experimentation, dynamic experiments, flow chemistry, kinetic, self‐optimization

## Abstract

Automated flow platforms are well‐established in the context of chemical reaction optimization leveraging techniques such as Design of Experiments and self‐optimization. However, the development of such platforms in the context of kinetic investigations proves challenging, as an underlying mechanistic model needs to be identified. In order to address these challenges, we have developed an automated dynamic flow experimentation platform to automatically fit and identify the most accurate model. The effectiveness of this platform was successfully demonstrated on three complex transition metal catalyzed transformations (Buchwald‐Hartwig reaction, Re‐catalyzed oxygen atom transfer and Cu‐catalyzed C─H activation), automatically performing dynamic flow experiments, automatically fitting the kinetic parameters and independently identifying the appropriate kinetic model from a set of candidates. The obtained models were subsequently optimized using multi‐objective Bayesian optimization and both Pareto‐optimal and non‐Pareto‐optimal points from each of the models were seamlessly transferred to continuous flow to validate the workflows efficacy.

## Graphical abstract

We have developed an automated dynamic flow experimentation platform to automatically fit and identify the most accurate kinetic model from a generated set of candidates. Three transition metal‐catalyzed transformations were performed using this workflow. The obtained models were subsequently optimized using Bayesian optimization and optimized conditions were transferred to continuous flow.

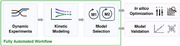

## 1 Introduction

In recent years, automated platforms have become increasingly common in synthetic chemistry laboratories and have been applied to addressing a number of chemical problems in both batch and flow [1, 2, 3]. Such automated platforms allow for large numbers of experiments to be carried out rapidly with only modest effort on the part of the experimentalist [4, 5, 6, 7, 8]. This in turn can be applied to improve process understanding or draw new conclusions about the chemical problem at hand.

Flow chemistry facilitates straightforward automation, as most commercial flow equipment readily supports remote control, resulting in the more widespread adoption of automation in flow chemistry [9, 10, 11, 12, 13, 14, 15]. Flow chemistry also facilitates the implementation of process analytical technology (PAT) allowing for experimental results to be obtained close to real time. Fast chromatographic methods like ultra‐high performance liquid chromatography (UHPLC) can produce analytical results on a minute timescale [16]. Of course, many of the inherent advantages of flow chemistry [17], such as better process safety [18], improved heat‐ and mass‐transfer [19], and efficiency [20] play a significant role in the desirability of designing processes using flow conditions. Process development relies on gaining detailed insights into the problem at hand [4, 21]. One of the crucial challenges to overcome when developing processes in flow is relatively high material consumption required to perform flow experiments, as the reactor needs to be equilibrated (“steady state”) prior to analysis. This is especially important when dealing with scarce or costly reagents and starting materials, such as transition metal catalysts, commercially unavailable substrates or precious metals. Developing kinetic models for process development, while of great importance is very costly in terms of material consumption. Employing dynamic experimentation, a wide range of data points can be obtained simultaneously in a more efficient fashion. This is accomplished by allowing the reactor to rapidly reach steady state at short residence times, then gradually ramping the parameters and continuously recording the reactor output, leading to a large number of diverse datapoints being collected in each experiment [22, 23]. For instance, varying the total flow rate of the reactor, time series data can be obtained [24, 25]. Assuming the employed flow reactor behaves as an ideal plug flow reactor (PFR) (meaning radial mixing is perfect, while axial mixing is nonexistent), varying the total flow rate within an experiment is exactly equal to performing a series of flow experiments, fully equilibrating the reactor each time [22]. Small‐diameter flow reactors exhibit excellent radial mixing and negligible axial mixing, meaning this ideal behavior can be assumed [26]. The high surface‐to‐volume ratio also means that heat transfer is excellent, making it easy to ensure isothermal conditions [17].

Kinetic investigations using this dynamic experimentation approach (the terms “time‐sweep”, “transient flow” and “ramp flow” have also been used interchangeably) and related techniques [27] have been applied to several common chemical engineering benchmarking reactions such as esterification [28, 29], Paal‐Knorr reaction [30], Knoevenagel condensation [31], carbamate formation from isocyanates [32], S_N_Ar [16, 33], and amide bond formation [34]. While these transformations are highly relevant in chemistry and chemical engineering and well established, the reaction networks of these transformations are relatively small and well‐understood. On the other hand, complex, transition metal catalyzed systems are underexplored using this technique due to their intricate reaction proceedings, allowing for a wide range of potential off‐cycle side reactions and large number of manipulable parameters, resulting in a challenging implementation. This highly relevant application requires for multiple engineering and chemical problems to be addressed, which thus far prevented thorough kinetic analysis of transition metal catalyzed transformations in this fashion. In a practical context, kinetic models today are often fitted by first identifying the reaction network, formulating the corresponding differential equations, then parametrizing those differential equations and fitting the associated parameters to real data using least squares methods [26]. This process can be challenging, as the identification of an appropriate reaction network often requires an iterative approach. To initially understand the catalytic cycles of transition metal reactions, graphical techniques like reaction progress kinetic analysis (RPKA) [35] are often implemented as well. Recently, computer‐aided techniques have also been developed leveraging machine learning methods to assist in identifying reaction networks based on obtained time series data [36, 37].

Complex reactions, such as transition metal catalysis, often behave in non‐ideal ways, especially in an intensified context [38]. Transition‐metal catalysis in particular is prone to a wide variety of side‐reactions due to the complexity of the process and the high reactivity of many of the transition metals employed, especially at higher temperatures. Many reactions of this type are well‐established and well‐understood, meaning that the basic reaction networks can be obtained from the literature, but to obtain a viable model for process development, it is necessary to quantify side reactions and deactivation steps to have an accurate representation of the reaction progress over time.

In this work, we have developed a fully automated, highly modular system that enables users to perform dynamic flow experiments, to fit kinetic models and to discriminate among models in an expedient and efficient manner. To demonstrate the effectiveness of the developed platform and workflow several transition metal catalyzed reactions have been investigated: a palladium‐catalyzed Buchwald‐Hartwig amination towards an API intermediate, the oxidation of a thioether compound by a rhenium complex and finally a copper‐catalyzed meta‐arylation of an aromatic amide using a diaryliodonium salt as a reagent.

## 2 Results and Discussion

### 2.1 Setup and Workflow

In order to automatically develop kinetic models for transition‐metal catalyzed reactions, an automated flow platform capable of accommodating these different catalytic systems was required.

**FIGURE 1:**
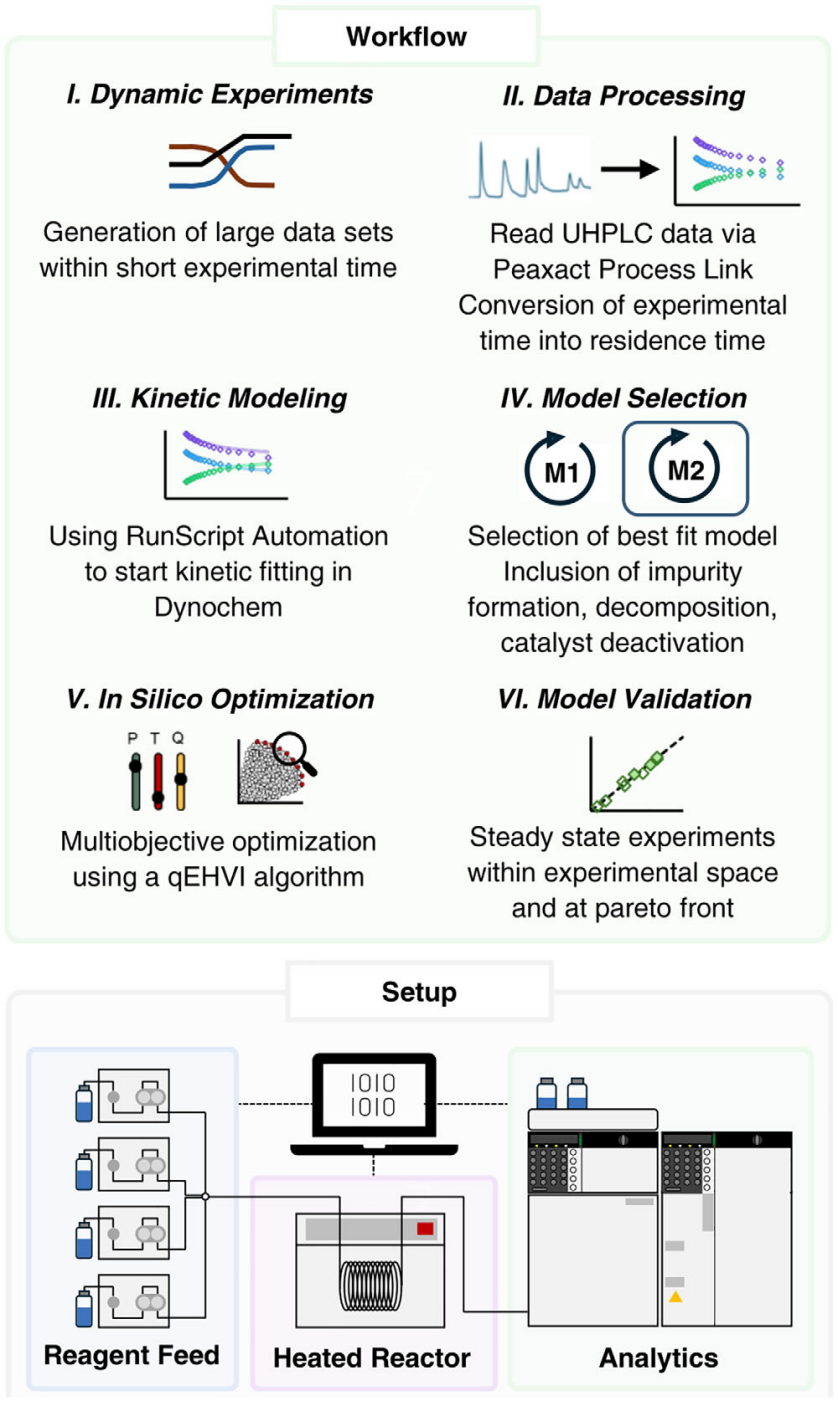
**Top**: Schematic representation of the developed workflow, steps 1 to 4 are carried out without any human intervention. **Bottom**: Schematic representation of automated flow chemistry setup.

The implementation of the necessary workflow (Figure 1 **Top**) was accomplished *via* an OPCUA server‐based approach. Such an approach allows for flexible implementation of experimental protocols and seamless transferability to new hardware (see Supporting Information for a detailed explanation of the software components). Using the OPCUA standard, device connections are established, allowing control of the relevant device parameters, as well as the retrieval of analytical results. The feeding unit of the setup is comprised of (up to) 5 Knauer HPLC pumps, delivering (up to) 4 reagent feeds and an additional solvent stream into a seven‐port mixer to prepare and introduce the reaction mixture into a heated 1/32″ PFA coil reactor. The feedstock pumps are precisely controlled by the control software over the course of the experiment. Once the thermostat reaches the desired temperature, the coil reactor is equilibrated with the desired reaction mixture composition at the initial residence time. Once the equilibration is complete, the flow rates of the pumps are steadily ramped downwards until the conclusion of the experiments. The residence time parameter is the only parameter changed continuously, as literature suggests that the potential benefit of changing many parameters simultaneously is small compared to the drastic increase in the complexity of determining the precise reaction conditions at each sample point. After passing through a heated coil reactor, the reaction mixture is transferred to the analytics using a 0.3 mm PTFA tubing in order to minimize the void volume. The reaction mixture is analyzed using UHPLC on a 3 min timescale, resulting in a theoretical output of 20 data points per experimental run. The resulting chromatograms are automatically processed, and the resulting concentration data is made available using Peaxact ProcessLink (S‐Pact), obtaining a number of calibrated concentration values for the key constituents of the reaction mixture. The residence time associated with the obtained concentration data is calculated automatically following the ideal plug flow reactor model (see further details in the Supporting Information).

After the experiment has concluded, the entire set of obtained raw data for the experiment is stored and automatically processed into a time series dataset in a. csv format. This dataset is then automatically imported into Dynochem [39] (Scale‐up Systems, Mettler Toledo) generating a set of candidate models. These are initially formulated based on plausible reaction mechanisms reported in the literature. Then, based on preliminary experiments and published reports, pathways for off cycles, such as side reactions and catalyst deactivation are formulated, resulting in a permuted set of several models. This set containing all the models is then automatically fitted using the RunScriptAutomation [40] (Scale‐up Systems, Mettler Toledo) python integration. Upon conclusion of the fit, the Residual Sum of Squares (SSQ) of each model is calculated. The model with the lowest SSQ among the candidates can be considered as the most appropriate model, as it exhibits the best goodness of fit. Other metrics were also contemplated (Dynochem model selection criterion, Akaike information criterion, Bayesian information criterion, mean average percentage error), but these metrics gave comparable results to the use of SSQ. This automated process allows for the full development of a complete, fit‐for‐purpose kinetic model within only 8 h.

The selected model has been used as the basis of a multi‐objective Bayesian optimization campaign, leveraging the low computation time of kinetic models to rapidly query the chemical space. The reaction is then optimized in silico, using the yield as well as objective relevant to each case study (e.g., catalyst loading, productivity, etc.). Finally, the kinetic model is validated by experimentally performing points from the simulated self‐optimization campaign. In effect, the workflow therefore results in a kinetic model for further in silico optimization and several sets of optimized conditions which can be considered as starting points for scaling up the reaction and further process development.

### 2.2 Case Study 1: Buchwald Hartwig Amination

**FIGURE 2:**
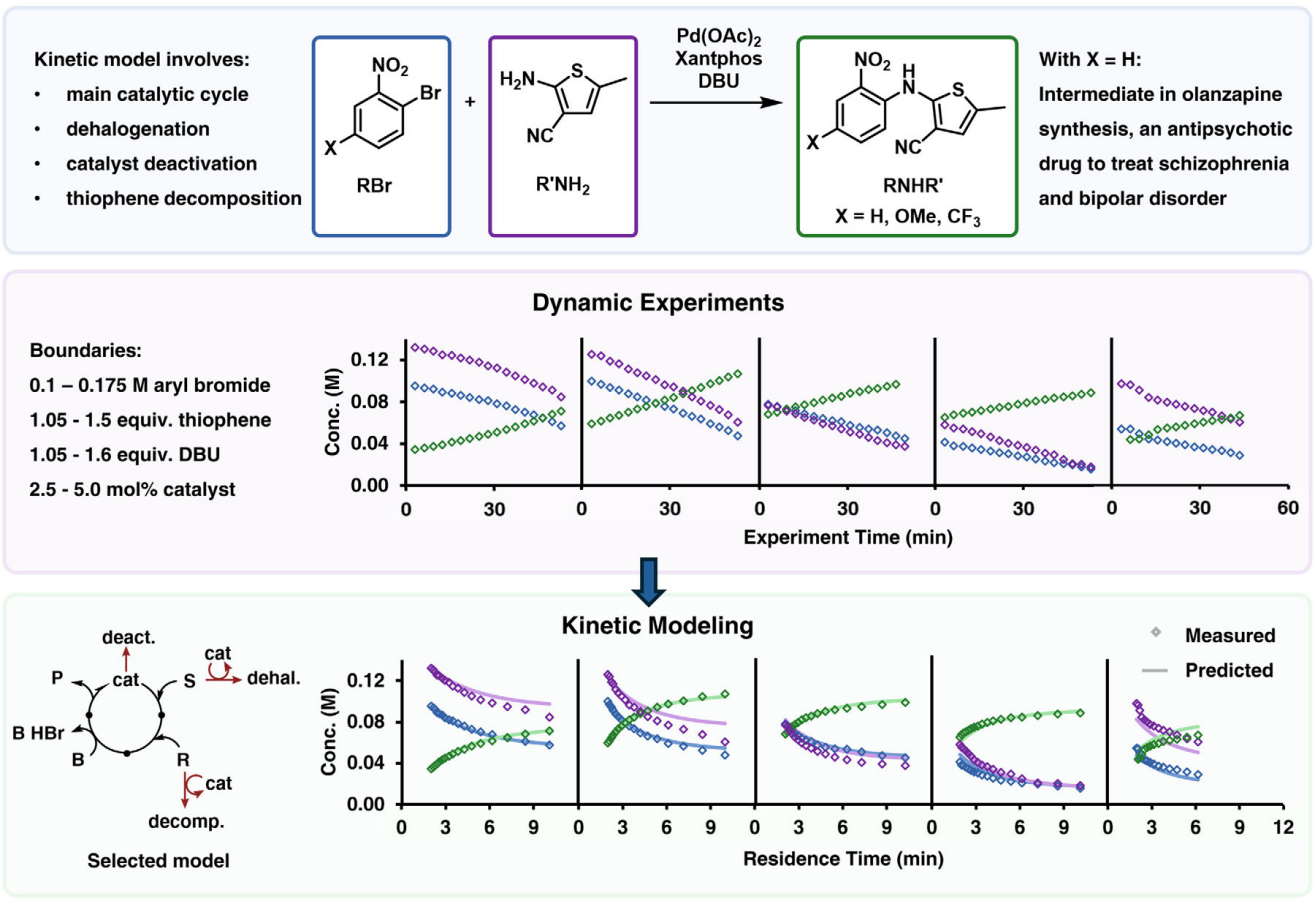
**Top**: Buchwald‐Hartwig amination to form an Olanzapine intermediate. **Middle**: Boundaries of optimization process and UHPLC concentrations plotted against experimental time. **Bottom**: Abbreviated, selected reaction model (cat: = Pd(OAc)_2_/Xantphos, S = RBr, R = R'NH_2_, P = RNHR’, deact = deactiviation of Pd catalyst, dehal. = dehalogenated RBr, decomp. = decomposition of R'NH_2_) and UHPLC concentration data transformed in the time domain to obtain reaction times. Measured results plotted as points, predicted results plotted as lines.

In the first case study, we decided to test the developed platform and workflow using well‐understood, but still challenging chemistry. The Buchwald‐Hartwig reaction is one of the most important transformations in the pharmaceutical industry to form C‐N bonds [41, 42] and has found a wide range of applications in both the initial drug discovery process as well as on production scale. Therefore, considerable effort has been invested into understanding the reaction in detail, making the catalytic cycle well‐established [41, 43]. Nonetheless, there are still challenges associated with utilizing this transformation in a practical fashion. The Buchwald‐Hartwig reaction exhibits considerable substrate dependence [41, 44]. This, coupled with the chemical complexity of pharmaceutically relevant starting materials can often lead to non‐ideal behaviors. These challenges within a well‐established chemical framework make the Buchwald‐Hartwig reaction an ideal case study for our investigation. We selected the Pd‐catalyzed cross coupling of 2‐bromonitrobenzene with a 2‐amino thiophene towards Red‐Orange‐Yellow (ROY) (Figure 2 **top**), an intermediate in the synthesis of the Active Pharmaceutical Ingredient (API) olanzapine as our model reaction. To accomplish this, 5 different parameters were varied in the model building process: concentration of the aryl bromide starting material, equivalents of the coupling partner, equivalents of base, catalyst loading and temperature. Five flow ramp experiments (Figure 2 **middle**), as well as a control without catalyst, were carried out, resulting in residence times from 2 min to 12 min. The species quantified in this reaction were the starting materials, the product and the nitrobenzene impurity. The automated UHPLC measurements resulted in 5 concentration profiles of all of the relevant, measurable constituents of the reaction mixture.

Upon conclusion of each automated flow ramp experiment, the raw data was transformed into time‐series data (Figure 2 **bottom**) and fitted automatically using Dynochem. The schematic catalytic cycle of the selected model is shown in Figure 2 **bottom**. In the first step, the activated PdL system undergoes oxidative addition with the aryl halide. Subsequently, the amine coordinates to this complex. After abstraction of the amine proton and the bromide by the base, the product can be formed *via* reductive elimination. It is important to mention that both the oxidative addition, as well as the reductive elimination can be the rate determining step in this transformation. Several side reactions are commonly reported for this type of transformation: base‐enabled dehalogenation of the aryl halide after the oxidative addition step, gradual deactivation of the Pd catalyst and decomposition of the thiophene starting material [38]. Based on this initial literature knowledge, we conceived a set of 8 plausible candidate models describing the reaction proceedings, including all possible permutations with the aforementioned side reactions. The most suitable model's results are plotted alongside the transformed UHPLC results in Figure 2 **bottom**. Based on the performed flow ramp experiments, reaction progress stalled after 6 to 7 min. The deviation between the theoretical model and the experimental results of thiophene starting material can be attributed to a more complex decomposition pathway than modeled. On the other hand, a very good fit for the other analytes could be observed with an overall good SSQ of 4.62 mM, indicating that the model matches both the analytical results and the reaction proceedings, with the oxidative addition functioning as the rate determining step.

To demonstrate the wider applicability of the workflow, two additional dynamic experimentation campaigns were carried out on two 4‐substituted 1‐bromo‐2‐nitrobenzenes, one with an electron‐donating OMe‐group and one with an electron‐withdrawing CF_3_‐group. An excellent quality of fit was obtained for both of the respective products (see Supporting Information for further details). The methoxy group slowed down the reaction rate considerably, resulting in lower yields compared to the unsubstituted starting material, but the overall reactivity trends and the oxidative addition as rate determining step remained the same. Meanwhile, the CF_3_‐group resulted in a faster overall reaction rate, as well as a significantly faster oxidative addition. In this case, the reductive elimination proceeds more slowly as the rate‐determining step. These observations closely match expected reactivity trends [44]. In short, after conducting several automated flow ramp experiment, a suitable model with suitable parameters could be indentified, resulting in a good quality of fit, allowing for further optimization to be carried out using that model.

### 2.3 Case Study 2: Re‐Catalyzed Oxygen Atom Transfer

For the second case study we turned our attention towards the transition‐metal catalyzed synthesis of sulfoxides. This moiety is commonly found in APIs and formed through oxidation of the corresponding thioethers [45, 46]. In a practical context, these types of reactions are often performed using bleach in water and is often affected by the formation of overoxidized side products. Alongside cross‐couplings, transition metals enable a variety of oxidation reactions, often with excellent selectivity. Therefore, we selected a catalytic oxygen atom transfer (OAT) reaction using a highly stable, dinuclear rhenium(VI) complex [47]. In this reaction, (per)chlorate or nitrate can serve as the oxygen donor [48, 49, 50]. While this catalyst was originally designed for the degradation of such pollutants present in wastewater, it also demonstrated potential synthetic application owing to its high stability in various solvents.

**FIGURE 3:**
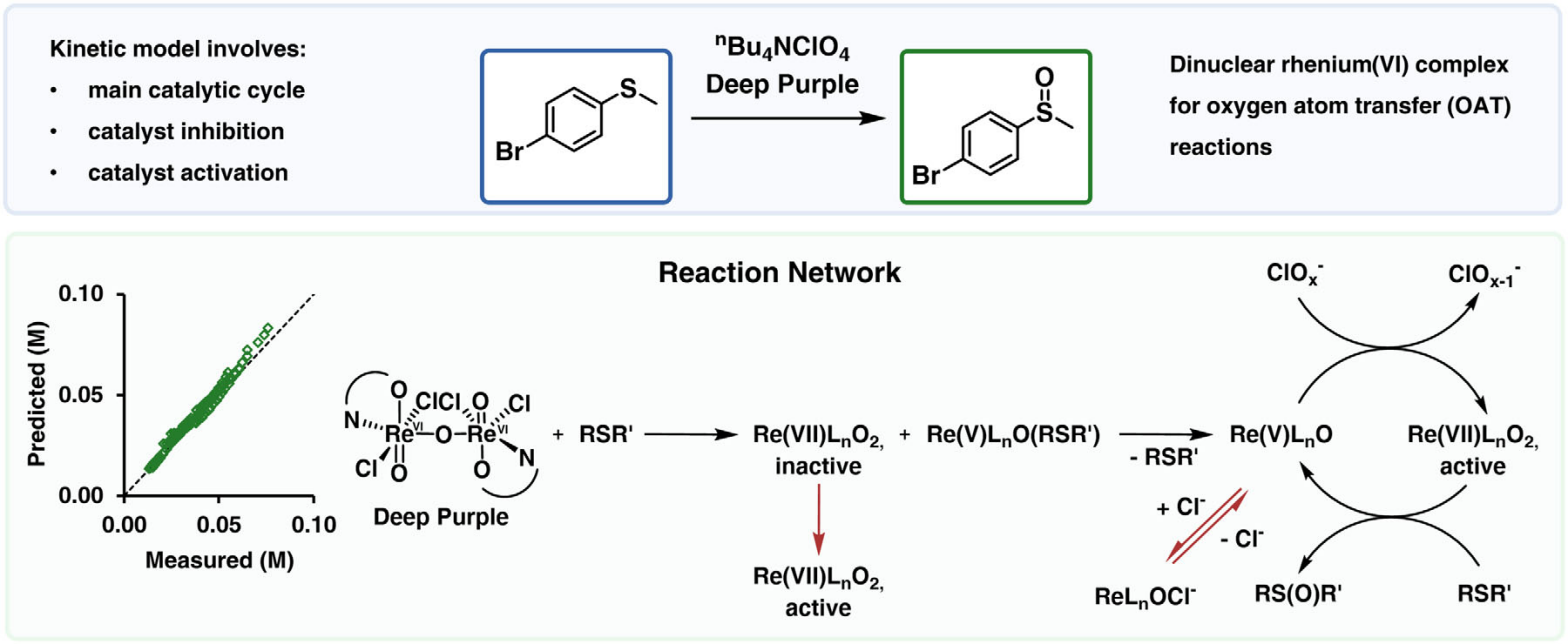
**Top**: Re‐catalyzed oxygen atom transfer from tetrabutylammonium perchlorate to 4‐bromothioanisol. **Bottom**: Predicted vs true plot for the fitted model and modelled reaction network of the oxygen atom transfer reaction.

We selected 4‐bromothioanisole (Figure 3 **top**) as the oxygen acceptor in our model reaction. This reaction proceeds *via* an activated Re(V)/Re(VII) cycle (Figure 3 **bottom**), abstracting an oxygen atom from the perchlorate on each cycle, until it is fully reduced to chloride.

The side pathways of interest considered were the catalyst inhibition by the fully reduced chloride as a reversible and irreversible reaction step and the activation of inactive Re(VII) species. To experimentally develop a kinetic model for this transformation, 6 automated dynamic flow reactions were carried out, as well as a control containing no catalyst, tracking the concentrations of both thioanisole and the respective sulfoxide product using online UHPLC. As expected, no reaction occurred within the control. Notably, the sulfone overoxidation product was not observed within any of the flow ramps performed, suggesting excellent selectivity for the desired sulfoxide product.

The best fitting model for this reaction includes reversible chloride inhibition, but no activation of the Re(VII) species, as it is not included in the identified best‐fitting model. Furthermore, upon inspection of the kinetic parameters obtained, it is apparent that the first reduction step from the perchlorate to the chlorate is the rate determining step, while the second reduction to chlorite still occurs at a relatively slow rate. Inhibition of the catalyst also occurs rapidly in the presence of chloride *via* reversible coordination. In the end, an excellent fit was obtained, with an SSQ of 0.37 mM.

In addition to the 4‐bromothioanisole, the same set of experiments was carried out for unsubstituted thioanisole. This compound followed the expected reactivity trends. Due to the more electron rich aromatic system, the transfer of the oxygen atom to the thioether was significantly slower than in the bromo‐substituted case, leading to this step being rate determining for this substrate, while the rate constants for the other steps remain virtually unchanged. In summary, the automated workflow identified a suitable kinetic model among the candidates with an excellent fit, using seven flow ramp experiments.

### 2.4 Case Study 3: Copper‐Catalyzed Meta‐Arylation

To complete this validation study, we decided to investigate the copper‐catalyzed *meta*‐arylation of pivalamides using diaryliodonium salts as the coupling partner [51]. This reaction attracted considerable attention owing to its non‐obvious regioselectivity as well as operational simplicity [52]. Nevertheless, the fundamental analysis of the reaction proceeding is cumbersome due to the promiscuous nature of the copper catalyst. For instance, this reaction requires specific reaction settings, namely the presence of 1,2‐dichloroethane in the solvent mixture [51]. In this sense, enabling the systematic investigation of such intricate reactions in an automated fashion demonstrates the robustness of this workflow.

**FIGURE 4:**
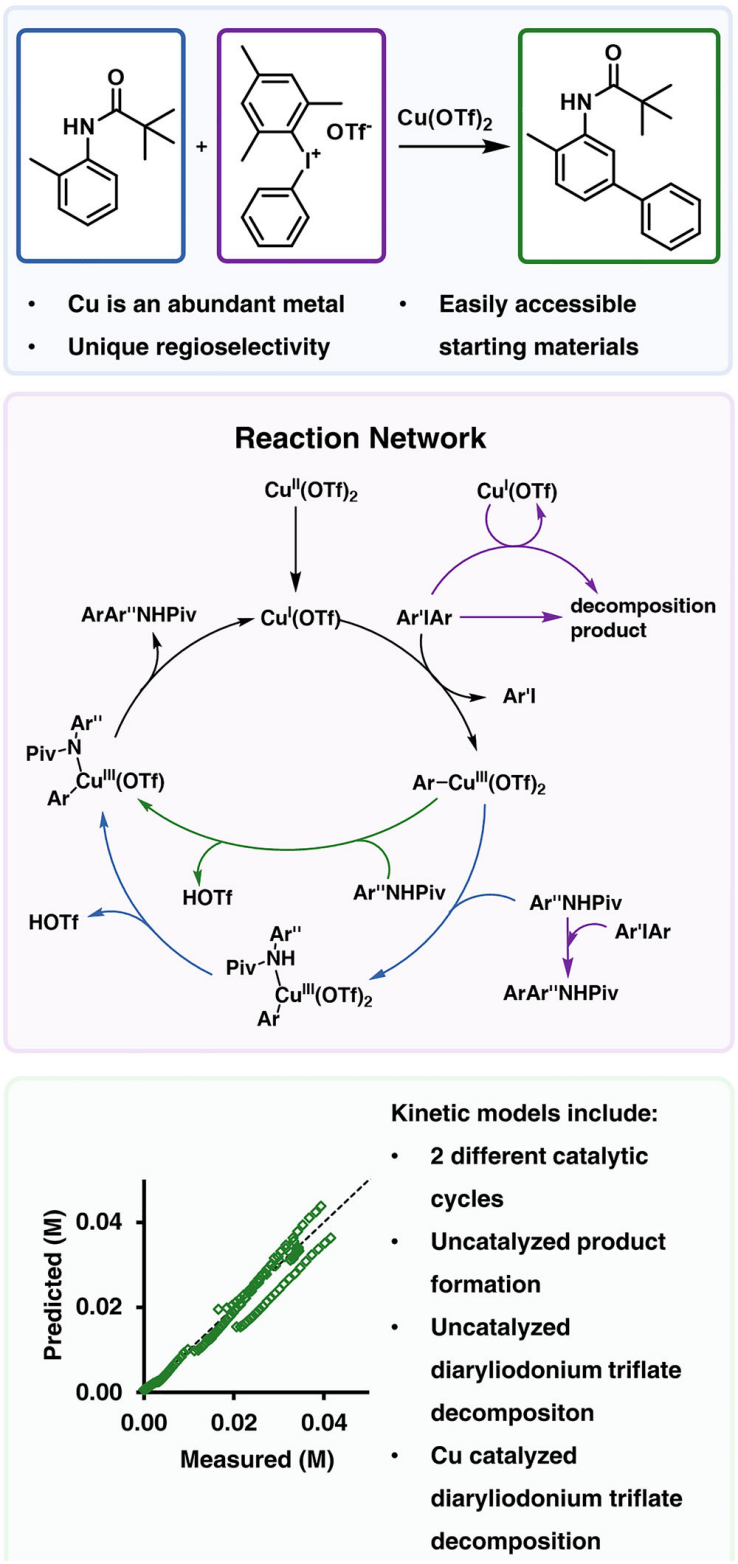
**Top**: Copper‐catalyzed, meta‐selective arylation of N‐(o‐tolyl)pivalamide by mesityl(phenyl)iodonium triflate. **Middle**: catalytic cycle of this transformation, including critical side reaction pathways. **Bottom**: predicted vs true plot of the best model identified.

We started our investigation based on literature reports [53], formulating a general reaction network (Figure 4 **middle**). First the copper catalyst is reduced to Cu(I), then the diaryliodonium salt oxidizes the copper, forming the arylated Cu(III) species in the process. Subsequently, either in a concerted or stepwise fashion, the N‐(o‐tolyl)pivalamide coordinates to the copper and is deprotonated alongside decoordination of triflic acid. This intermediate then undergoes reductive elimination forming the product and regenerating the copper catalyst. Several side reactions in this process are literature‐known or expected. The key side reactions in this transformation are the direct C‐C bond formation by the diaryliodonium salt without the participation of the Cu‐catalyst and the thermal or copper‐catalyzed decomposition of the diaryliodonium salt starting material. These considerations resulted in a total of 16 candidate models, making it very expensive to obtain the solution to this problem in a manual fashion.

Six flow ramp experiments as well as a control experiment without catalyst were carried out for this reaction. The species selected to be quantified by online UHPLC in this case study were the arylamide, the diaryliodonium salt and the m‐arylation product. In the control experiment only traces of product were observed, as previously reported. Thermal decomposition of the diaryliodonium salt proved to be a limiting factor for the intensification of the reaction. At 120° the formation and consumption of starting material and product remained constant throughout the entire flow ramp, while consumption of the remaining diaryliodonium salt could be observed over time. After executing the developed automated protocol, the best fitting model included both thermal and Cu‐catalyzed contribution towards the diaryliodonium salt decomposition, achieving a good fit with an SSQ of 2.78 mM (Figure 4 **bottom**).

### 2.5 In Silico Optimization and Model Validation

To further demonstrate the utility and validity of the obtained kinetic models, a self‐optimization campaign was carried out for each of the case studies. Self‐optimization was performed using the qEHVI multi‐objective algorithm [54] implemented using BOTorch [55] (see Supporting Information for further detail). The aim was to simultaneously optimize the yield alongside two additional objectives for each of the case studies leveraging the kinetic model to efficiently perform the optimization process. The self‐optimization was initialized using a random sample of 20 points, followed by 40 iterations of the algorithm, obtaining a solution in the form of a Pareto front.

Leveraging this “frugal twin” approach, a Bayesian optimization strategy can be employed to optimize reactions quickly and cost efficiently, once the kinetic model has been obtained. However, there are some considerations when employing such a strategy. One potential limitation is complexity in the chemical system that cannot easily be captured by a relatively simple kinetic model, such as heterogeneous behavior of a transition metal catalyst [56] and autocatalytic catalyst deactivation, especially far outside the original design space, as it is very challenging to accurately quantify every single aspect of a chemical reaction. Furthermore, the outcome of this approach is heavily reliant on the quality of the frugal twin used in the machine learning optimization process. However, despite these challenges, this approach can be employed to efficiently solve complex multi‐objective optimization problems in a fraction of the time it would take experimentally, while simultaneously reducing material costs.

For the Buchwald‐Hartwig reaction, minimizing the loading of palladium catalyst and maximizing molar productivity were selected as additional optimization objectives, due to the relative cost of palladium and the observed plateau in the kinetic study resulting in full conversion after a certain reaction time. Input parameters to be optimized were temperature (80 – 140°C), concentration of 2‐bromonitrobenzene (80 – 160 mM), equivalents thiophene (1.0 – 2.0), equivalents DBU (1.0 – 2.0), catalyst loading (3.0 – 7.0 mol%), and reaction time (2 – 15 min) for a total of 6 input variables and 3 output variables. As expected, catalyst loading is a key factor with regards to the speed of this reaction, with the productivity being strongly correlated with catalyst loading. However, good yields can still be achieved using lower catalyst amounts and longer reaction times. Furthermore, the productivity levels off with increasing catalyst loading, suggesting an upper limit to the impact of the residence time on the outcome of the reaction. Moreover, temperature also has a significant effect on the reaction outcome, with all Pareto optimal results being performed at the maximum temperature within the design space of the optimization campaign.

**FIGURE 5:**
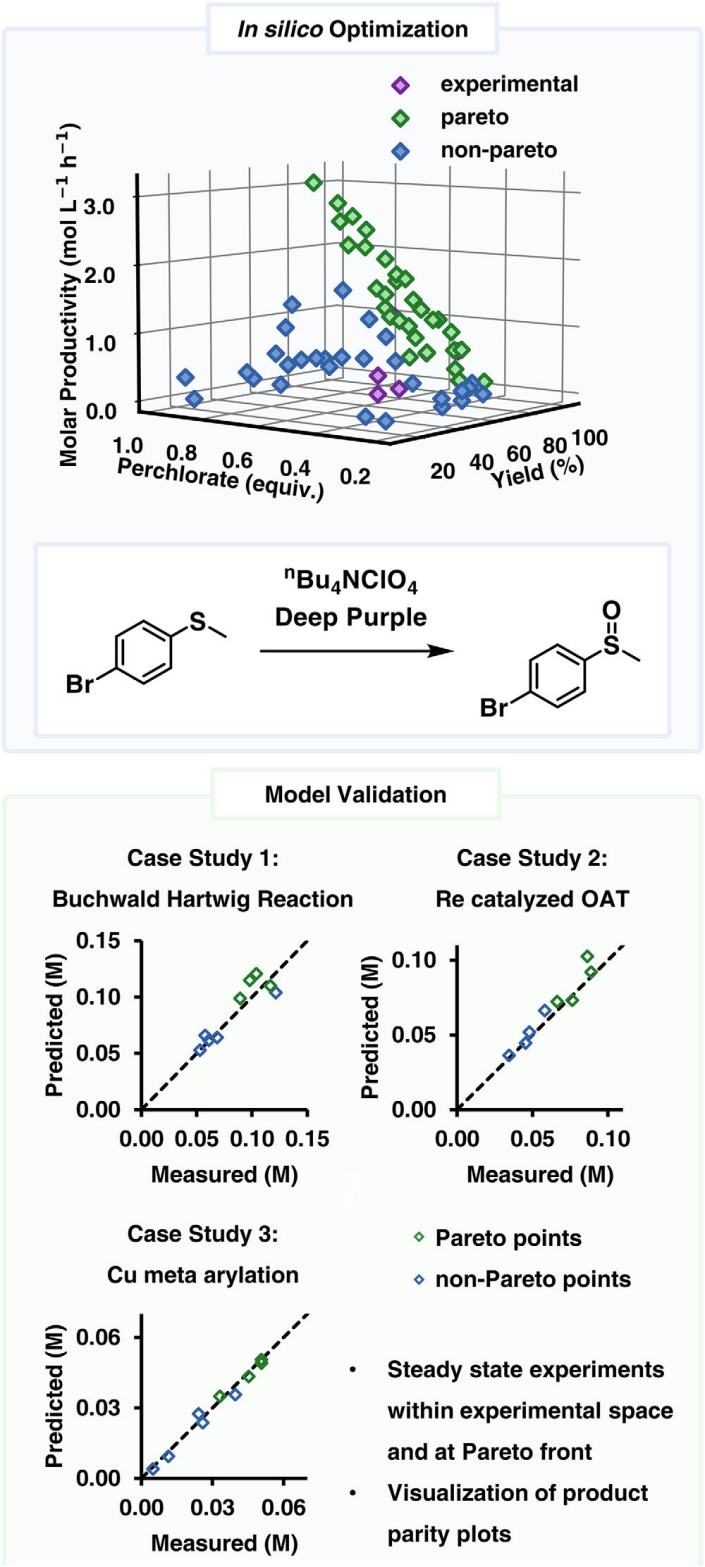
**Top**: 3D plot of in silico multi‐objective optimization of case study 3, green: Pareto values, blue: non‐Pareto values, purple: experimental results. **Bottom**: parity plots of the product of the steady state validation experiments.

The second case study was optimized adopting a similar approach, maximizing yield and molar productivity, but the third objective was changed to optimize the stoichiometry of perchlorate instead, as ideally only 0.25 equivalents of perchlorate are required to fully oxidize the starting material. The input parameters to be optimized were temperature (50 – 90°C), concentration of the anisole (80 – 150 mM), equivalents of perchlorate (0.2 – 1.0), catalyst loading (0.4 – 1.0 mol%) and reaction time (2 – 15 min). As depicted in Figure 5 **top** the green points clearly show the Pareto front, alongside the non‐Pareto optimal points highlighted in blue. The optimization algorithm efficiently identified a large number of values on the Pareto front within 5 min. Furthermore, a selection of these points was also confirmed experimentally (highlighted in purple). Of particular note is the large number of results with moderate yields, but high molar productivity, showing a strong correlation between residence time and yield. The optimization algorithm also identified a large number of Pareto values that employ low equivalents of perchlorate, while still leading to good yields.

For the final copper catalyzed C‐H activation reaction, the non‐yield objectives were selected to minimize the amount of copper, as well as the diaryliodonium salt. The decision to minimize the diaryliodonium salt was made, because many of these salts are not commercially available. The variables to be tuned by the algorithm were temperature (60 – 140°C), amide concentration (50 – 85 mM), diaryliodonium equivalents (1.0 – 3.0), catalyst loading (1 – 60 mol%) and reaction time (2 – 15 min). A strong correlation between both catalyst loading and equivalents of diaryliodonium salt and the resulting yield can be observed. Based on the results of the multi‐objective optimization campaign, maintaining a precise temperature plays a significant role in this reaction, as increasing it too far leads to rapid decomposition of the diaryliodonium salt and low yields, even at high equivalents of the coupling partner.

To validate the results obtained during the in silico self‐optimization campaigns and to validate the model building process, a series of steady‐state flow experiments were carried out. The reactor was operated in continuous flow mode, and eight different sets of reactor inputs were performed for each of the case studies. Four of the points in each validation campaign were selected from among the Pareto front of the self‐optimization campaign, while the remaining points were chosen within the entire dataset. The obtained analytical results at these experimental inputs were then compared to the outputs of the kinetic models (Figure 5b). In each of the case studies it can be observed that the predicted results and the analytically obtained outcomes match closely across a wide range of concentrations, finding an RMSE of 11.1 mM for the product of the Buchwald‐Hartwig reaction, 7.45 mM for the Re‐catalyzed OAT and 2.22 mM for the Cu‐catalyzed C‐H activation reaction.

## 3 Conclusion

In conclusion, we developed and validated a workflow using an automated dynamic experimentation platform for the fitting of several complex transition metal catalyzed reactions. A palladium catalyzed C‐N cross coupling, rhenium‐catalyzed thioether oxidation and a copper‐catalyzed C‐H activation reaction were successfully investigated, and accurate models were automatically developed for these reactions. The automated platform was designed to be easily adaptable and amenable to most automated chemistry problems, with flexible OPCUA integration and user‐friendly procedure development. The platform employs fully automated analytics, efficiently addressing the challenges of dynamic experimentation. Leveraging an automated error minimization kinetic fitting approach, the process of fitting practical kinetic models has been greatly accelerated. Notably, one case study was fitted within 8 h. Finally, leveraging powerful machine learning techniques, we have optimized the reaction from the obtained models and validated the developed workflow by executing the optimized conditions in a steady‐state flow reactor. We believe that this innovative approach will greatly serve the community in enabling the rapid exploitation of transition metal catalyzed reactions to streamline their process applications.

## Author Contributions

**Florian L. Wagner**: writing – review and editing, writing – original draft, conceptualization, methodology, software, investigation, validation, formal analysis. **Klara Silber**: conceptualization, methodology, writing – review and editing, visualization, investigation, validation, formal analysis. **Tobias A. Doliner**: investigation. **C. Oliver Kappe**: supervision, resources, project administration, writing – review and editing, funding acquisition.

## Conflicts of Interest

The authors declare no conflict of interest.

## Supporting information




**Supporting File 1**: anie72698‐sup‐0001‐SuppMat.pdf.

**Supporting File 2**: anie72698‐sup‐0002‐Data.zip.

## Data Availability

The data that support the findings of this study are available in the supplementary material of this article.
