# Supplementary material for: Automated Dynamic Flow Experimentation for Rapid Kinetic Fitting of Transition Metal Catalysis
Source: Angew Chem Int Ed Engl. 2026 May 15;65(28):e6944441. doi: 10.1002/anie.6944441 (PMC13340506; doi:10.1002/anie.6944441)
Supplement: Supplementary file 1 — Supporting File 1: anie72698‐sup‐0001‐SuppMat.pdf. [file ANIE-65-e6944441-s002.pdf]

Supporting Information
©Wiley-VCH 2021
69451 Weinheim, Germany

Automated Dynamic Flow Experimentation for Rapid Kinetic Fitting of Transition Metal Catalysis

Florian L. Wagner,[a,b]+ Klara Silber,[a,b] + Tobias A. Doliner[c] and C. Oliver Kappe[a,b]*

[a] Institute of Chemistry, University of Graz, NAWI Graz, Heinrichstrasse 28, 8010 Graz, Austria

[b] Center for Continuous Flow Synthesis and Processing (CCFLOW), Research Center Pharmaceutical Engineering GmbH (RCPE), Inffeldgasse 13, 8010 Graz, Austria.

[c] Institute of Chemistry, University of Graz, NAWI Graz, Schubertstraße 1, 8010 Graz, Austria

\+ Contributed Equally

*Corresponding author: C. Oliver Kappe, Email: xxxx@email.com, ORCID: https://orcid.org/0000-0003-2983-6007

Abstract: Automated flow platforms are well-established in the context of chemical reaction optimization leveraging techniques such as Design of Experiments and self-optimization. However, the development of such platforms in the context of kinetic investigations proves challenging, as an underlying mechanistic model needs to be identified. In order to address these challenges, we have developed an automated dynamic flow experimentation platform to automatically fit and identify the most accurate model. The effectiveness of this platform was successfully demonstrated on three complex transition metal catalyzed transformations (Buchwald-Hartwig reaction, Re-catalyzed oxygen atom transfer and Cu-catalyzed C-H activation), automatically performing dynamic flow experiments, automatically fitting the kinetic parameters and independently identifying the appropriate kinetic model from a set of candidates. The obtained models were subsequently optimized using multi-objective Bayesian optimization and both Pareto-optimal and non-Pareto-optimal points from each of the models were seamlessly transferred to continuous flow to validate the workflows efficacy.

Table of Contents

1. General experimental details ... 3
2. General Flow Configuration ... 3
2.1. Case Study 1: Buchwald Hartwig Reaction ... 4
2.2. Case Study 2: Rhenium catalyzed thioanisole oxidation ... 4
2.3. Case Study 3: Copper catalyzed meta-selective coupling ... 5
3. Process Analytical Technology ... 6
3.1. Online UHPLC ... 6
3.1. Inline FTIR ... 11
4. Residence Time Distribution ... 11
5. Automation and Data Handling ... 13
5.1. Device communication ... 13
5.2. Execution of experiments ... 13
5.3. Data Processing ... 14
5.4. Automatic Kinetic Fitting ... 14
6. Automated Dynamic Experiments ... 15
6.1. Case Study 1: Buchwald Hartwig Reaction ... 16
6.1. Case Study 2: Rhenium catalyzed thioanisole oxidation ... 18
6.2. Case Study 3: Copper catalyzed meta-selective coupling reaction ... 20
7. Automated Kinetic Fitting ... 21
7.1. Case Study 1: Buchwald Hartwig Reaction ... 21
7.2. Case Study 2: Rhenium catalyzed thioanisole oxidation ... 27
7.3. Case Study 3: Copper catalyzed meta-selective coupling reaction ... 31
8. *In Silico* Optimization ... 38
8.1. Case Study 1: Buchwald Hartwig Reaction ... 38
8.2. Case Study 2: Rhenium catalyzed thioanisole oxidation ... 41
8.3. Case Study 3: Copper Catalyzed meta-selective Coupling ... 44
9. Model Validation ... 47
9.1. Case Study 1: Buchwald Hartwig Reaction ... 47
9.1. Case Study 2: Rhenium catalyzed thioanisole oxidation ... 48
9.1. Case Study 3: Copper Catalyzed meta-selective Coupling ... 49
10. Product Synthesis ... 50
10.1. Case Study 1: Synthesis of Buchwald Hartwig Products ... 50
10.2. Case Study 3: Synthesis of Starting Materials and Product of Copper Catalyzed meta-selective Coupling ... 51
11. NMR Spectra ... 52
References ... 59

1. General experimental details

Solvents and chemicals were purchased from commercial suppliers and used without further purification. 1-bromo-2-nitrobenzene (98 %), 2-fluoronitrobenzene (99 %), $Pd(OAc)_2$ (> 99.9 %), thioanisole (> 99 %), trimethylacetylchloride (99 %), mesitylene (98 %), iodobenzene (98 %), $Cu(OTf)_2$ (98 %), ethanol (> 99.8 %), dichloroethane (> 99 %) and 2-methyltetrahydrofurane (> 99 %) were purchased from Sigma Aldrich. 1-Bromo-2-nitro-4-(trifluormethyl)benzene (98 %), 1-bromo-4-methoxy-2-nitrobenzene (97%), 2-amino-5-methyl-3-thiophenecarbonitrile (97 %), 4-bromothioanisole (99.34 %), 1-bromo-4-(methylsulfinyl)benzene (98 %), 4-bromophenylmethylsulfone (99.72 %), methylsulfinylbenzene and methylphenylsulfone (99.99 %) were obtained from BLD pharm. Xantphos (95 %) was obtained from Fluorochem. 1,8-diazabicyclo[5.4.0]undec-7-ene (DBU, 98 %), tetrabutylammonium perchlorate (> 98 %), triethylamine (> 99 %) and trifluormethanesulfonic acid (> 98 %) were purchased from TCI. *o*-Toluidin (98 %) was obtained from Alfa Aesar. 2-Chloroperbenzoic acid (70-75 % in water), chlorobenzene (>99.9 %) and chloroform (99.9 %, extra dry) were obtained from ThermoScientific. Ethylacetate (> 99.8 %) and dichloromethane (> 99%) were purchased from VWR.

2. General Flow Configuration

The reactions were performed in a 4.67 mL coil reactor (case study 1 and 2) or a 4.84 mL coil reactor (case study 3) (PFA, 0.8 mm i.d.) and the reaction stream was analyzed using a Mettler Toledo ReactIR 702L and a Shimadzu Nexera X2 UHPLC (Figure S1). The feed solutions were delivered using Knauer AZURA P 4.1S HPLC pumps (10 mL/min pump head, Hastelloy or ceramic with pressure sensor). A back pressure regulator (BPR, Upchurch, P-465) equipped with a 34 bar (green, P-765) cartridge was attached directly after each HPLC pump. A 7-port mixer (IDEX P-151, 83 µL i.V.) was used to combine the inlet streams. The coil reactor was connected using PFA tubing (0.8 mm i.d.) and heated using a thermostat (Huber, Ministat 240). The reaction mixture was analyzed using inline FTIR (Mettler Toledo, React IR 702L) with a flow cell (Mettler Toledo, DS Micro Flow Cell Diamond). PTFE tubing (0.05 mL, 0.3 mm i.d.) was used to connect the outlet of the reactor to the FTIR and the FTIR to the sampling point of the UHPLC (Shimadzu, Nexera X2) (0.10 mL, 0.3 mm i.d.). A membrane-based BPR (Zaiput, BPR-10), set to 8 bar was integrated after the UHPLC. A photograph of the flow setup in the lab is shown in **Figure S1**.

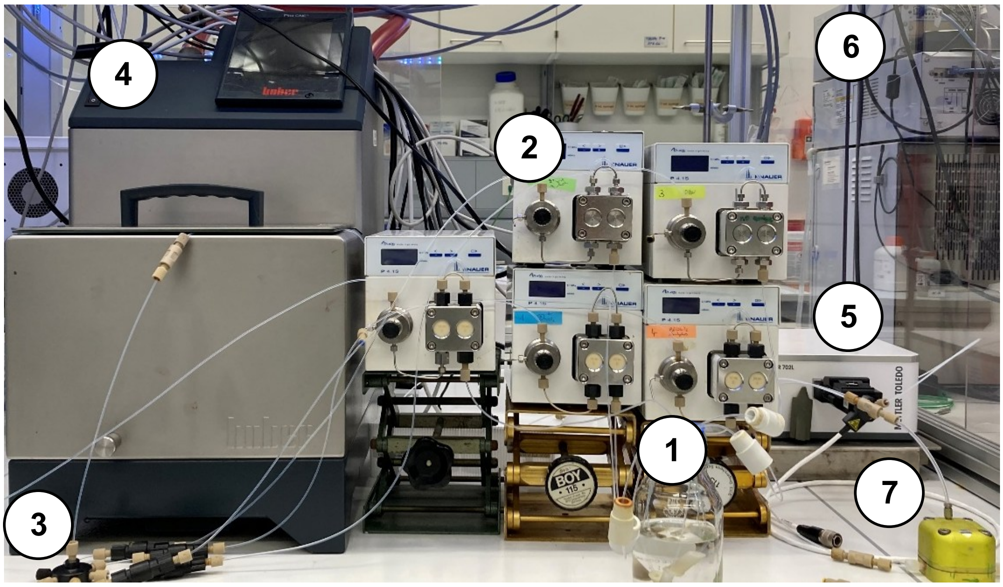

Figure S1. Schematic picture of the flow setup in the lab. (1) feeds, (2) HPLC pumps, (3) 7-port mixer, (4) heated thermostat with coil reactor insides, (5) FTIR, (6) UHPLC, (7) BPR.

2.1. Case Study 1: Buchwald Hartwig Reaction

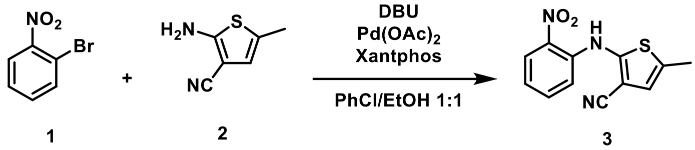

Scheme S1. Buchwald Hartwig reaction.

Stock solutions were prepared according to the following procedure:

<u>700 mM 2-bromonitrobenzene (**1**) stock solution:</u> A 100 mL volumetric flask was charged with 14.14 g of **1** and filled with chlorobenzene/ethanol 1:1.

<u>840 mM 2-amino-5-methyl-3-thiophenecarbonitrile (**2**) stock solution:</u> A 100 mL volumetric flask was charged with 11.60 g of **2** and filled with chlorobenzene/ethanol 1:1.

<u>980 mM DBU stock solution:</u> A 100 mL volumetric flask was charged with 14.92 g of **3** and filled with chlorobenzene/ethanol 1:1.

<u>27.5 mM Palladium(II)acetate, 41.25 mM Xantphos stock solution:</u> A 50 mL volumetric flask was charged with 309 mg $Pd(OAc)_2$ and 1.19 g Xantphos, set under inert conditions and filled with chlorobenzene.

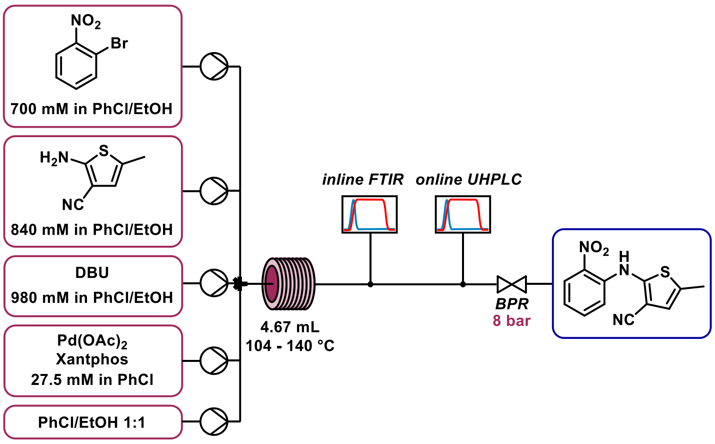

Figure S2. Flow setup Buchwald Hartwig reaction.

2.2. Case Study 2: Rhenium catalyzed thioanisole oxidation

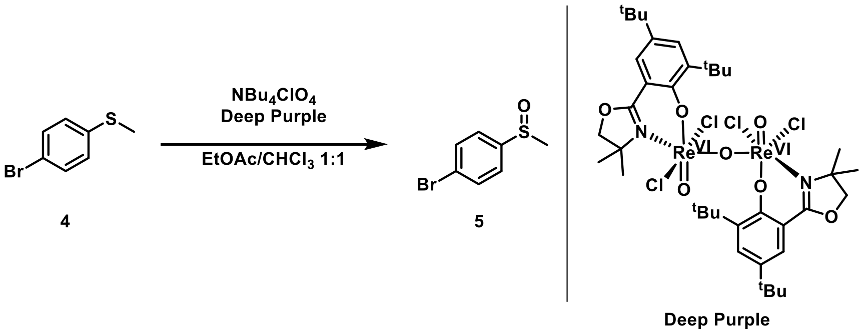

Scheme S2. Rhenium catalyzed thioanisole oxidation.

Stock solutions were prepared according to the following procedure:

<u>300 mM 4-bromothioanisole (**4**) stock solution:</u> A 100 mL volumetric flask was charged with 6.09 g of **4** and filled with ethyl acetate/chloroform 1:1.

<u>150 mM tetrabutylammonium perchlorate stock solution:</u> A 100 mL volumetric flask was charged with 5.12 g of tetrabutylammonium perchlorate and filled with ethyl acetate/chloroform 1:1.

5 mM Deep Purple (dinuclear µ-oxo bridged rhenium(VI) complex) (**DP**) stock solution: A 50 mL volumetric flask was charged with 291.8 mg of **DP** and filled with ethyl acetate/chloroform 1:1.

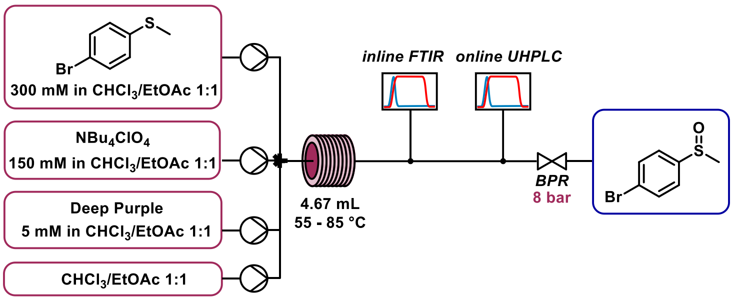

Figure S3. Flow setup rhenium catalyzed thioether oxidation reaction.

2.3. Case Study 3: Copper catalyzed meta-selective coupling

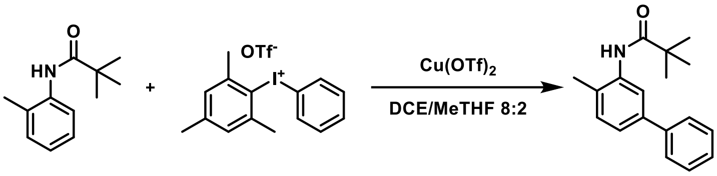

6 7 8

Scheme S3. Copper catalyzed meta-selective coupling reaction.

Stock solutions were prepared according to the following procedure:

300 mM *N*-(*o*-tolyl)pivalamide (**6**) stock solution: A 100 mL volumetric flask was charged with 5.72 g of **6** and filled with dichloroethane/2-methyltetrahydrofuran 8:2.

420 mM mesityl(phenyl)iodonium triflate (**7**) stock solution: A 100 mL volumetric flask was charged with 19.82 g of **7** and filled with dichloroethane/2-methyltetrahydrofuran 8:2

60 mM copper(II)triflate: A 100 mL volumetric flask was charged with 2.16 g of $Cu(OTf)_2$ and filled with dichloroethane/2-methyltetrahydrofuran 8:2

The precise concentration of **6** and **7** in the respective stock solutions was confirmed *via* UHPLC measurement directly before flow experiments were conducted.

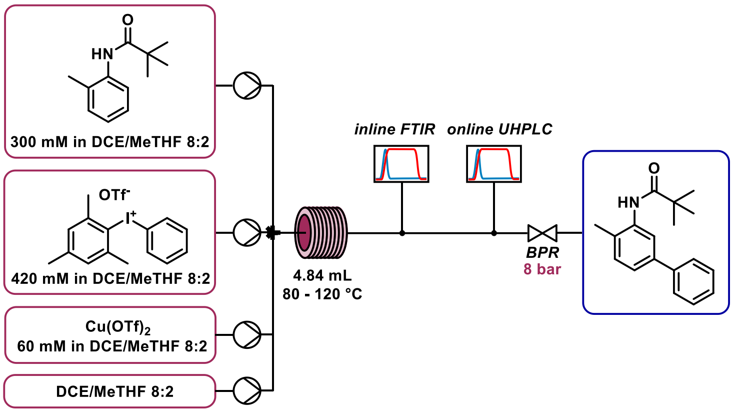

Figure S4. Flow setup copper catalyzed meta-selective coupling reaction.

3. Process Analytical Technology

3.1. Online UHPLC

The UHPLC-DAD (Shimadzu, Nexera X2) consists of a degassing unit (DGU-403ASR), two solvent delivery units (LC-30AD), a thermostated column oven (CTO-20AC), a diode array detector (SPD-M30A) and a control unit (CBM-20A). Analysis was carried out using a reversed-phase column (Phenomenex Luna Omega C18 (50 x 2.1 mm, particle size 1.6µm, pore size 100 Å)) at 45 °C using a total flow rate of 1 mL/min. The sample was introduced by an internal injection valve (10 nL, 20000 psi, Cheminert Nanovolume, Part# C84U-6674-.01EUH), which was triggered by the CBM-20A control unit.

Solvent A: H_2O + MeCN 9 + 1 (v/v) + 0.1% HCOOH
Solvent B: MeCN + 0.1% HCOOH

Case Study 1: Buchwald Hartwig Reaction

Method
0.00-0.10 min: 30% solvent B
0.10-0.80 min: ramp to 50% solvent B
0.80-1.20 min: 50% solvent B
1.20-1.70 min: ramp to 100% solvent B
1.70-2.21 min: 100% solvent B
2.21-3.00 min: 30% solvent B

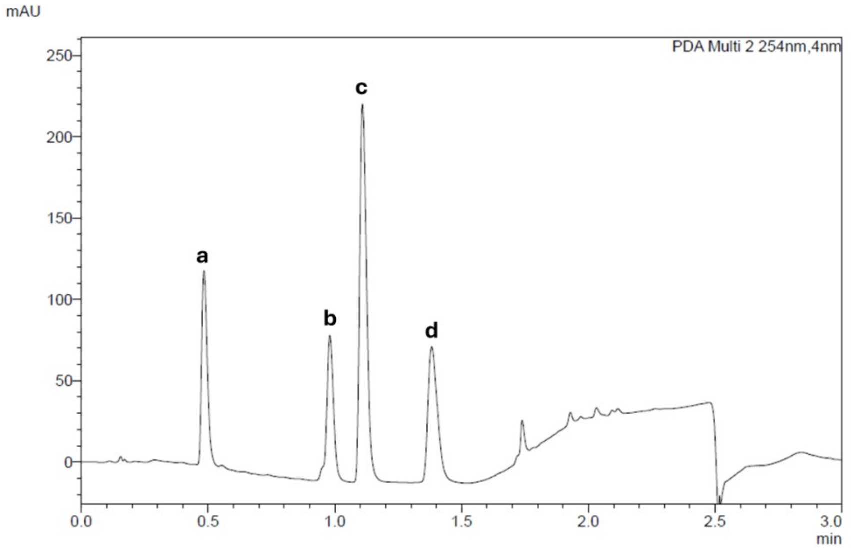

Figure S5. Example chromatogram of the reaction mixture of dynamic experiments of the Buchwald Hartwig reaction. (a) 2-amino-5-methyl-3-thiophenecarbonitrile (**2**), (b) 2-bromonitrobenzene (**1**), (c) phenylchloride (solvent), (d) Buchwald Hartwig product (**3**).

Calibration

Calibration solutions of 2-bromonitrobenzene (**1**), 2-amino-5-methyl-3-thiophenecarbonitrile (**2**) and calibration solutions of product (**3**) and of nitrobenzene (5 – 15 mM) were prepared in 1 - 5 mL volumetric flasks in chlorobenzene/ethanol 1:1 in concentrations ranging from 20 – 200 mM. Each calibration solution was injected *via* the 6-port valve and analyzed three times. All compounds were calibrated at 254 nm using a Peaxact (S-PACT) integration model (Figure S6).

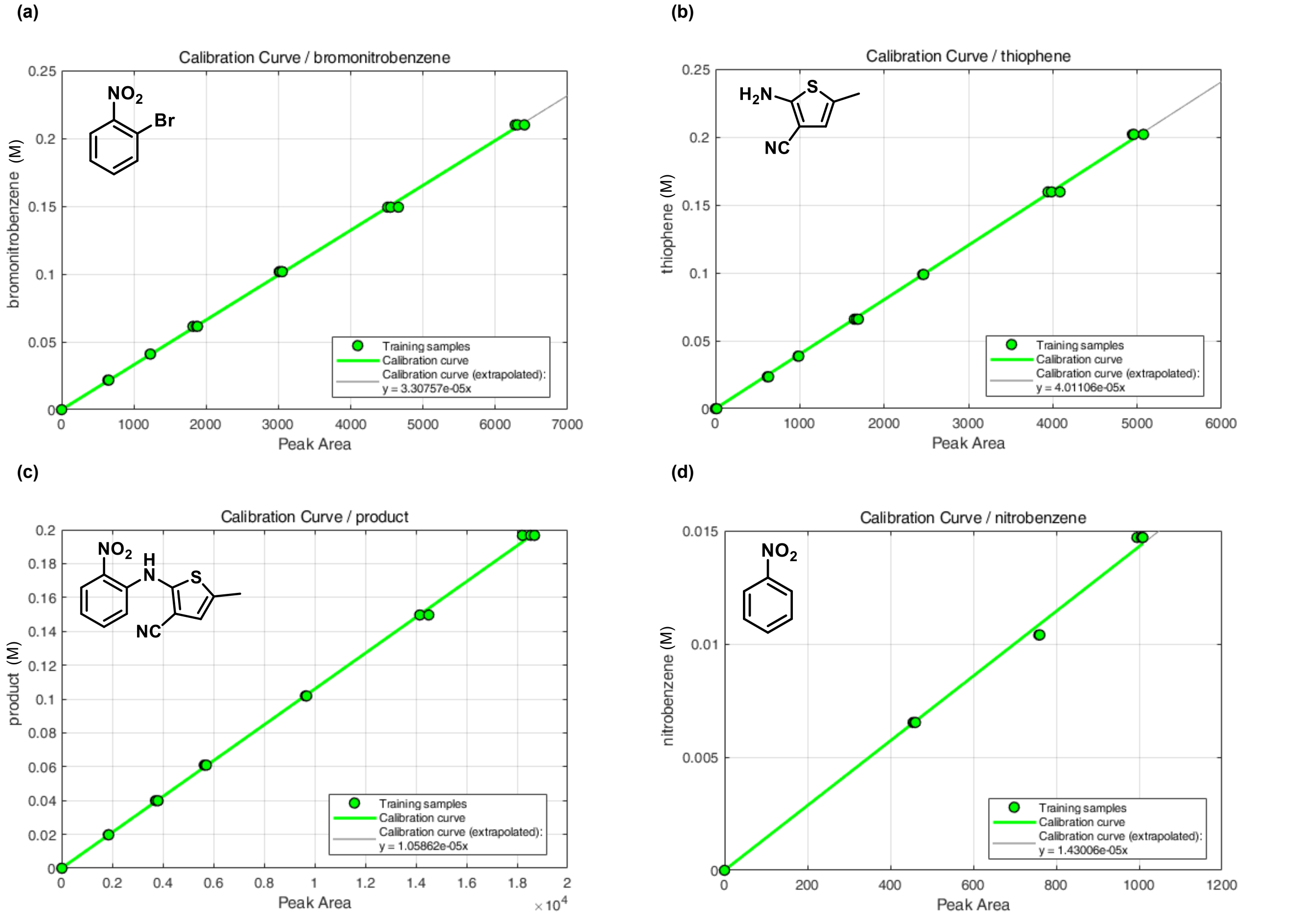

Figure S6. Calibration curves applying the integration model from Peaxact for (a) 2-bromonitrobenzene (**1**) (R^2 = 0.995), (b) 2-amino-5-methyl-3-thiophenecarbonitrile (**2**) (R^2 = 0.973), (c) Buchwald Hartwig product (**3**) (R^2 = 0.993), (d) nitrobenzene (R^2 = 0.996).

1-bromo-4-methoxy-2-nitrobenzene (**1a**) and 1-bromo-2-nitro-4-(trifluormethyl)benzene (**1b**) were used as additional substrates for the Buchwald Hartwig reaction. **1a** and **1b** together with the corresponding coupling products **3a** and **3b** (synthesized in the lab) were calibrated as well and a Peaxact integration model was built for quantification. **1a** and **3a** were quantified at 300 nm due to an overlay of **1a** with the solvent peak. For **1b** and **3b** the model was built from the peak areas at 254 nm. Calibration curves are shown in Figure S7 for **1a** and **3a** and in Figure S8 for **1b** and **3b**.

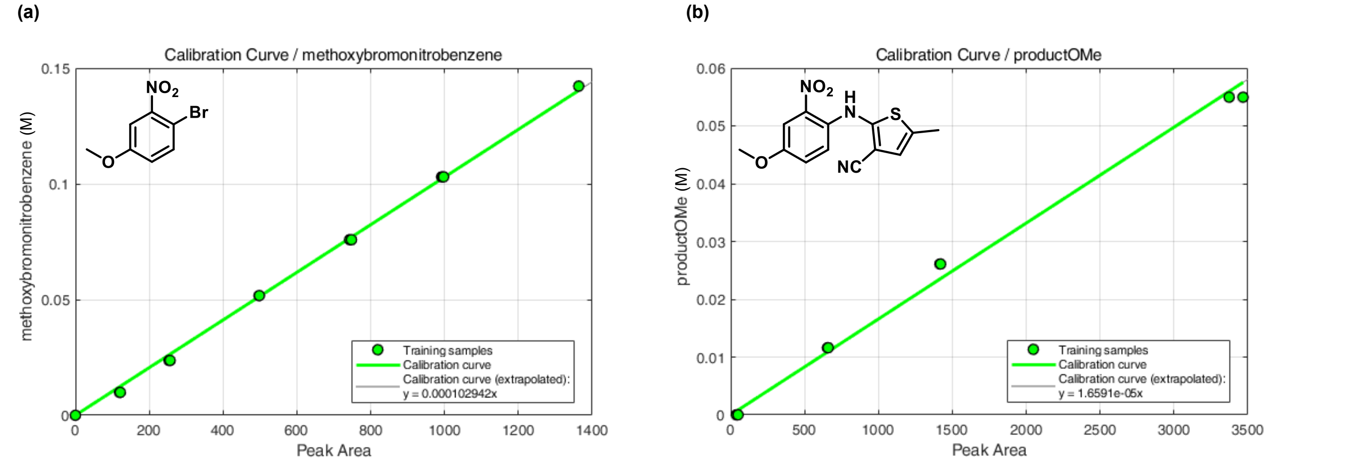

Figure S7. Calibration curves applying the integration model from Peaxact for (a) 1-bromo-4-methoxy-2-nitrobenzene (**1a**) (R^2 = 0.999), (b) methoxy substituted product (**3a**) (R^2 = 0.993).

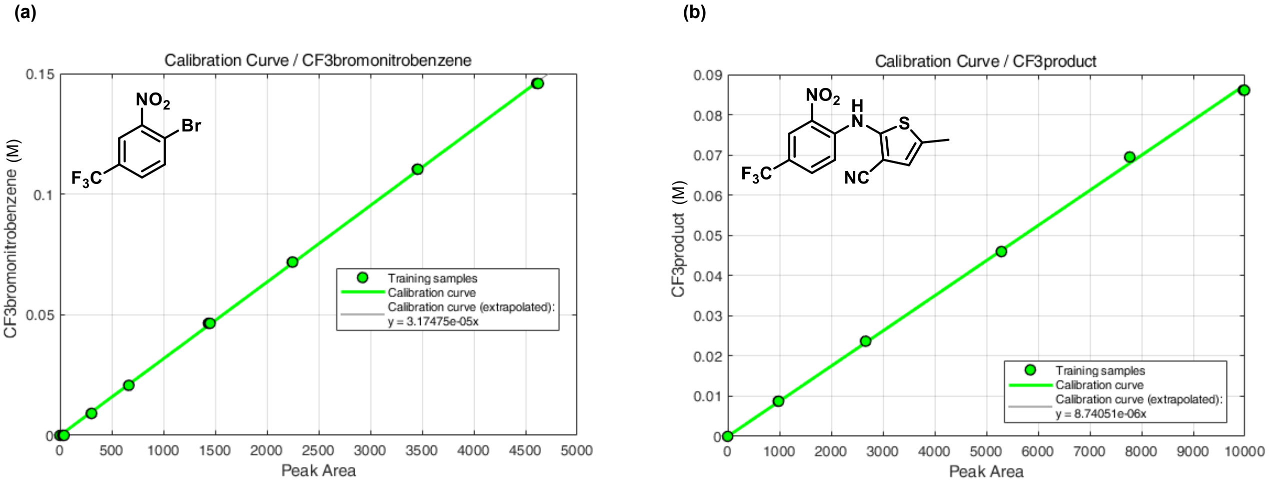

Figure S8. Calibration curves applying the integration model from Peaxact for (a) 1-bromo-2-nitro-4-(trifluormethyl)benzene (**1b**) (R^2 = 0.999), (b) trifluormethyl substituted product (**3b**) (R^2 = 0.999).

Case Study 2: Rhenium catalyzed thioanisole oxidation

Method

0.00-0.10 min: 20% solvent B
0.10-0.75 min: ramp to 30% solvent B
0.75-1.50 min: ramp to 100% solvent B
1.50-2.00 min: 100% solvent B
2.01-2.50 min: 20% solvent B

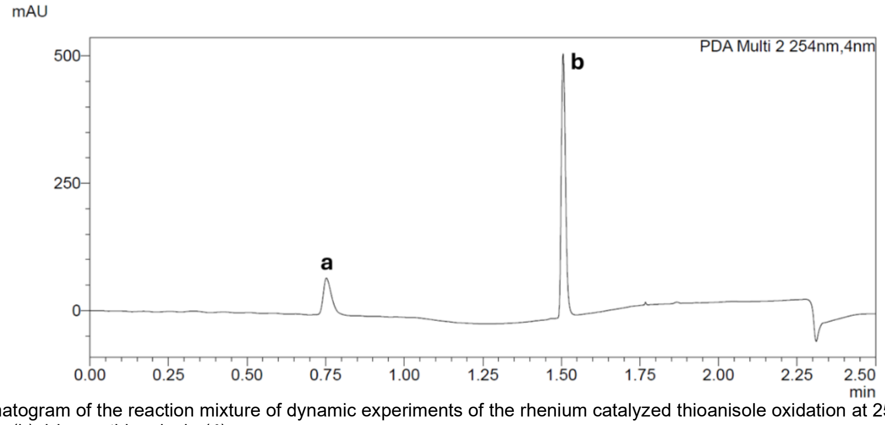

Figure S9. Example chromatogram of the reaction mixture of dynamic experiments of the rhenium catalyzed thioanisole oxidation at 254 nm. (a) 1-bromo-4-(methylsulfinyl)benzene (**5**), (b) 4-bromothioanisole (**4**).

Calibration

Calibration solutions of 4-bromothioanisole (**4**) and 1-bromo-4-(methylsulfinyl)benzene (**5**) were prepared in 2 - 20 mL volumetric flasks in chloroform/ethylacetate 1:1 in concentrations ranging from 5 - 100 mM. Each calibration solution was injected *via* the 6-port valve and analyzed three times. All compounds were calibrated using the peak area at 254 nm with a Peaxact integration model (Figure S10).

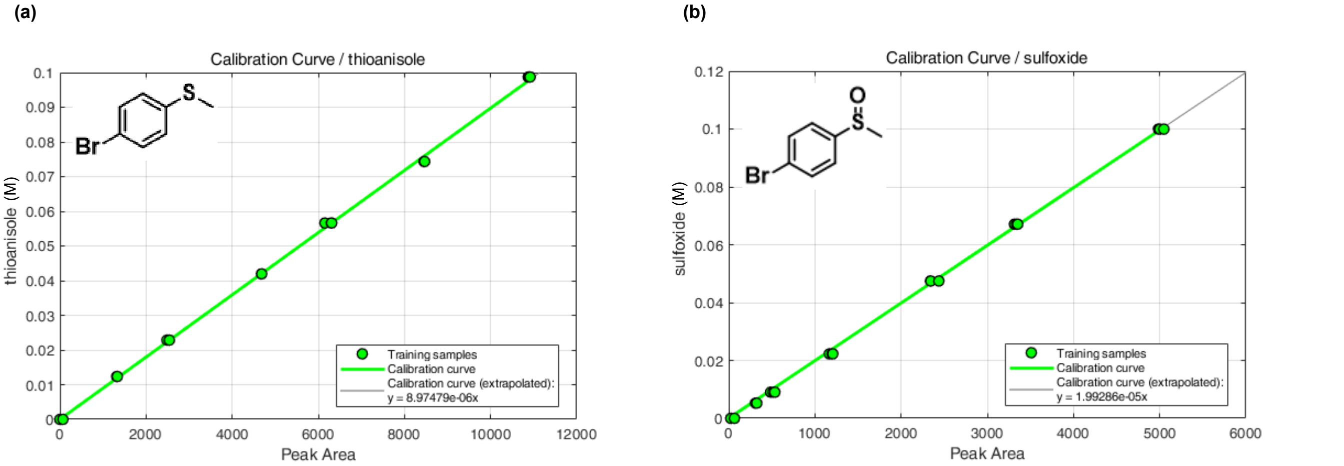

Figure S10. Calibration curves applying the integration model from Peaxact for (a) 4-bromothioanisole (**4**) (R^2 = 0.999), (b) 1-bromo-4-(methylsulfinyl)benzene (**5**) (R^2 = 0.999).

Additionally, thioanisole (**4a**) was used as substrate for the rhenium catalyzed thioether oxidation. **4a** and the sulfoxide **5a** were calibrated as well and a Peaxact integration model from peak areas at 254 nm was built (Figure S11).

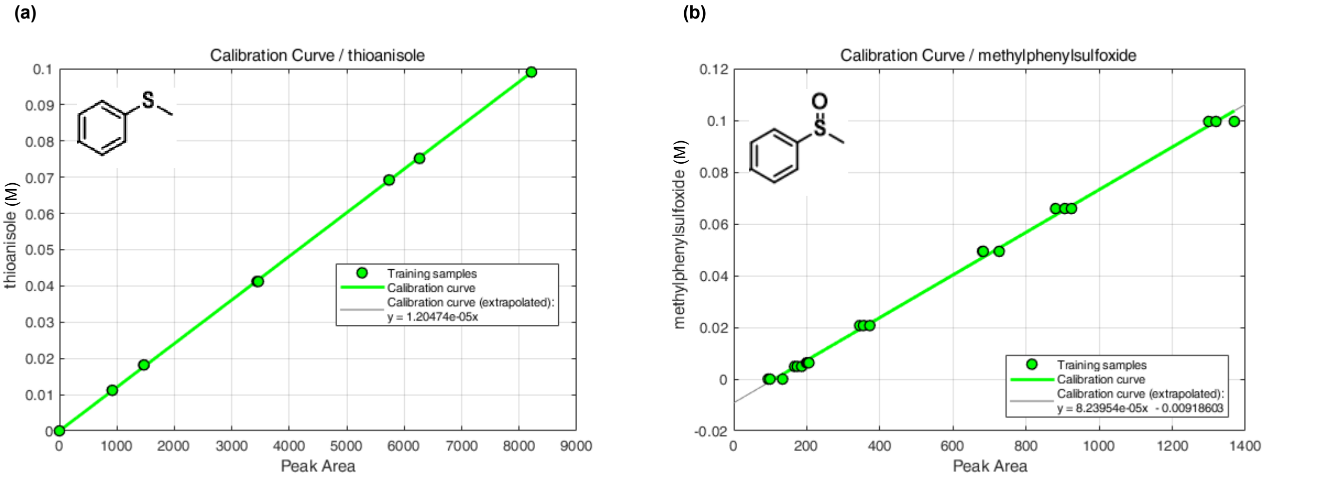

Figure S11. Calibration curves applying the integration model from Peaxact for (a) thioanisole (**4a**) (R^2 = 0.999), (b) (methylsulfinyl)benzene (**5a**) (R^2 = 0.998).

Case Study 3: Copper catalyzed meta-arylation

Method

0.00-0.10 min: 30% solvent B
0.10-1.50 min: ramp to 100% solvent B
1.50-1.70 min: 100% solvent B
1.71-2.40 min: 30% solvent B

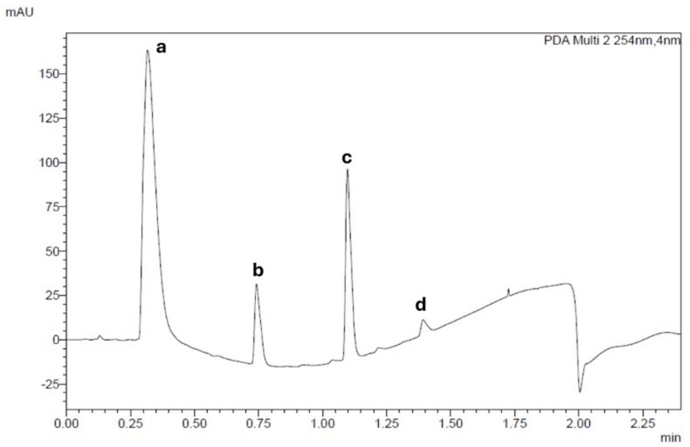

Figure S12. Example chromatogram of the reaction mixture of dynamic experiments of the copper catalyzed meta-selective coupling reaction at 254 nm. (a) mesityl(phenyl)iodonium triflate (**7**), (b) N-(o-tolyl)pivalamide (**6**), (c) product (**8**), (d) decomposition product of **7**.

Calibration

Calibration solutions of *N*-(*o*-tolyl)pivalamide (**6**) and mesityl(phenyl)iodonium triflate (**7**) and product (**8**) were prepared in 1 - 5 mL volumetric flasks in 1,2-dichloroethane/2-methyltetrahydrofuran 8:2 in concentrations ranging from 10 – 250 mM. Each calibration solution was injected *via* the 6-port valve and analyzed three times. All compounds were calibrated using the peak area at 254 nm with a Peaxact integration model (Figure S13).

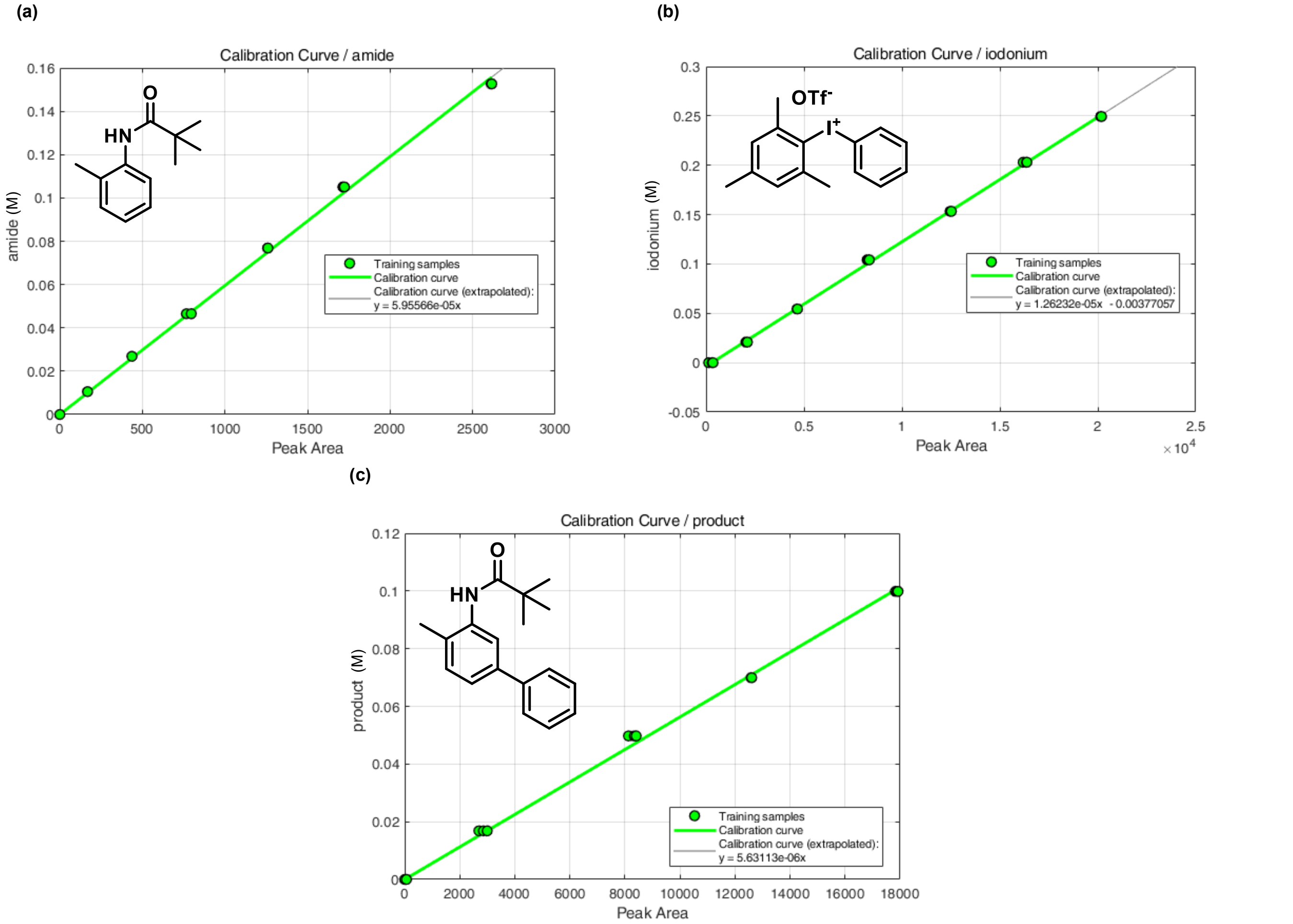

Figure S13. Calibration curves applying the integration model from Peaxact for (a) *N*-(*o*-tolyl)pivalamide (**6**) (R^2 = 0.998), (b) mesityl(phenyl)iodonium triflate (**7**) (R^2 = 0.999)., (c) product (**8**) (R^2 = 0.998).

3.1. Inline FTIR

Inline FTIR spectra were recorded on a Mettler Toledo ReactIR 702L equipped with a flow cell (Mettler Toledo, DS Micro Flow Cell Diamond). The acquisition time for each data point was 15 s. Spectra were recorded between 4000 and 600 cm^{-1} using the maximum resolution of 4 cm^{-1}. For the 1st case study the peak at 1532 cm^{-1}, for the 2nd case study the peak at 1092 cm^{-1}, and for the 3rd case study the peak at 1450 cm^{-1} was used to monitor the reaction progress without quantification.

4. Residence Time Distribution

The flow setup (Figure S14) was used to determine the residence time distribution at 120°C and three different flow rates (1 mL/min, 0.5 mL/min, 0.33 mL/min). The residence times were investigated by changing the concentration of 2-bromonitrobenzene using a step up (0 mol/L to 0.7 mol/L) followed by a step down (0.7 mol/L to 0 mol/L) function. Concentration values were obtained using inline FTIR (measurement interval 5 s) using a peak integration model from 1504 – 1560 cm^{-1}.

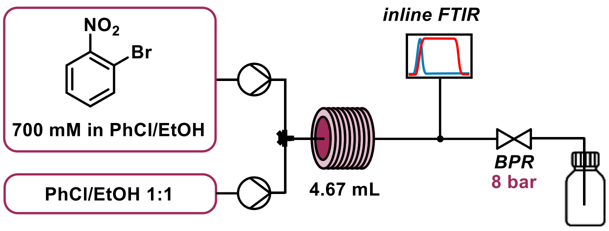

Figure S14. Flow setup for residence time distribution experiments.

Residence time, Bodenstein number and axial dispersion number were calculated as described in Levenspiel[1] and given in Table S1. The mean residence time t_{res} was calculated according to Equation 1, where t_i is the time from the start of the step input, Δc_i the concentration difference between two adjacent measurement points and c_{max} the maximum concentration within an experiment.

$$t_{res} \cong \frac{\sum_i t_i \cdot \Delta c_i}{c_{max}} \quad with \ \Delta c_i = c_i - c_{i-1} \quad (1)$$

The variance σ² (Equation 2) represents the spread of the distribution when it passes the reactor outlet.

$$\sigma^2 \cong \frac{\sum_i t_i^2 \cdot \Delta c_i}{c_{max}} - t_{res}^2 \quad (2)$$

The variance was used to obtain the Bodenstein number Bo (Equation 3). A Bodenstein number higher than 100 indicates plug flow behavior, while a value below 100 indicates more CSTR-like behavior.

$$Bo = \frac{2 \cdot {t_{res}}^2}{\sigma^2} \quad (3)$$

The axial dispersion number D_{ax} was calculated according to Equation 4 where u is the flow velocity and L the length of the reactor.

$$D_{ax} = \frac{u \cdot L}{Bo} \quad (4)$$

Table S1. Calculated residence time, Bodenstein number and axial dispersion number at a reactor temperature of 100 °C - 140 °C.

Temperature (°C)	Flow rate (mL/min)	tres (s)	Bo (-)	Dax (m^2/s)
100	1.00	291	283	1.1E-3
100	0.50	576	579	2.7E-4
100	0.33	872	904	1.1E-4
110	1.00	288	164	1.9E-3
110	0.50	575	467	3.3E-4
110	0.33	876	852	1.2E-4
120	1.00	289	241	1.3E-3
120	0.50	570	502	3.1E-4
120	0.33	868	880	1.2E-4
130	1.00	285	179	1.7E-3
130	0.50	574	462	3.3E-4
130	0.33	866	707	1.4E-4
140	1.00	284	110	2.8E-3
140	0.50	571	429	3.6E-4
140	0.33	872	685	1.5E-4

The Bodenstein number is higher than 100 in all experiments, meaning that plug flow behavior can be assumed and the impact of axial dispersion is negligible, similarly temperature dependence of the real residence time is also negligible. This behavior matches the literature found for similar continuous flow reactors as well.[2][3] Radial temperature gradients within the reactor are not expected to occur due to the small internal diameter of the reactor tubing (1/8") and full submersion in a thermostated oil bath. The path length from the tube wall to the center is only ~0.16 cm and the thermal reservoir of the oil bath is several orders of magnitude larger than the tube's overall volume.

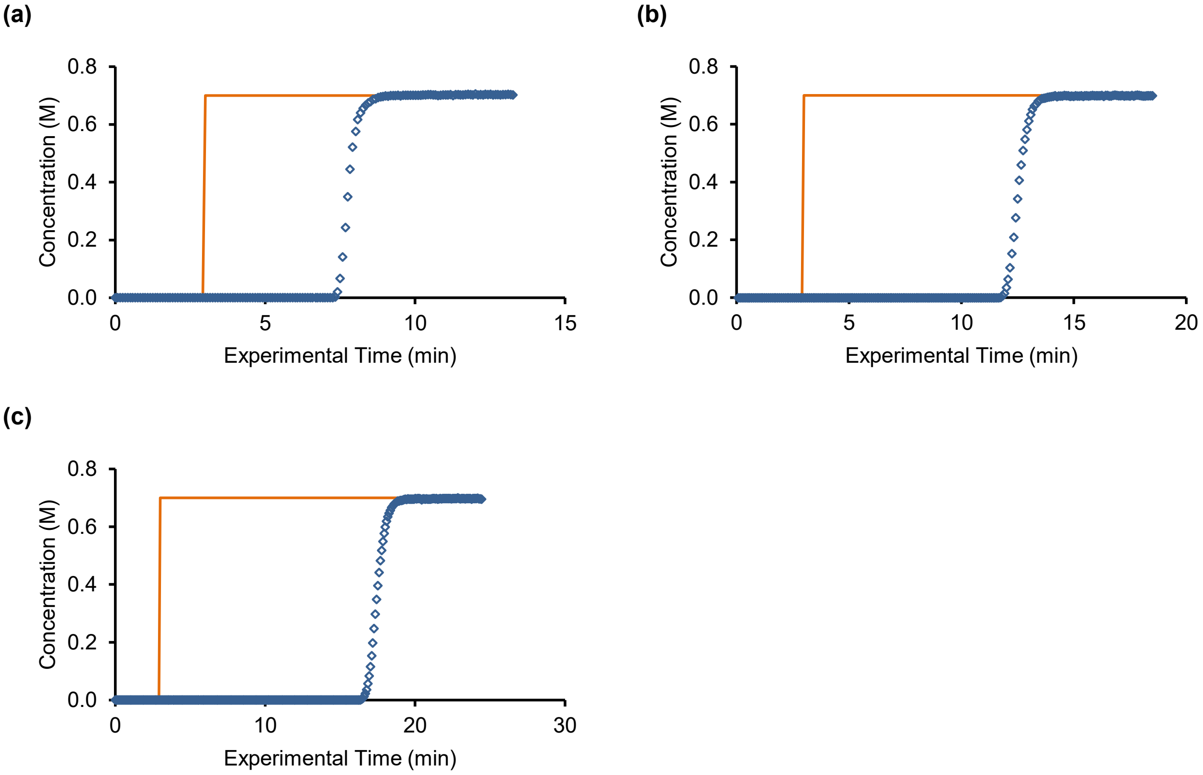

Figure S15. Residence time distribution experiments at 100°C. Visualization of input concentration (orange) and output concentration at the FTIR (blue) for (a) 1 mL/min, (b) 0.5 mL/min, (c) 0.33 mL/min.

5. Automation and Data Handling

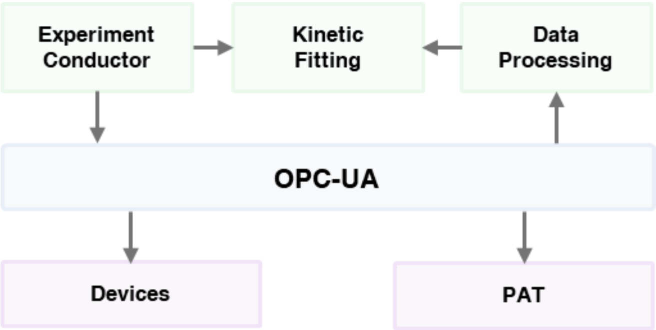

Figure S16. Device communication *via* OPC-UA server.

5.1. Device communication

Device parameters (flow rates, temperature setpoints) were controlled using the HiTech Zang LabManager box *via* an RS232 connection. The lab manager includes OPCUA functionality, allowing for the external manipulation of device parameters. A custom python software was developed to interface with this OPCUA server (Figure S16). To allow for a highly flexible and modular architecture, the device parameters have been implemented using a YAML serialization approach (Figure S17). Devices are read and connected automatically based on a library of device parameters. To process analytical results in real time, Peaxact Process Link was used to provide an OPCUA server automatically processing the developed analytical models. Similar to the experimental equipment, the device parameters of the analytical equipment have also been implemented using a YAML serialization approach. This is crucial, as it makes the developed software system hardware agnostic. To add new devices from the interface layer, device types only need to be implemented once and can be referenced easily afterwards.

```yaml
! devices.yaml
devices:
  - id: "Pump1"
    type: "Pump"
    opcua:
      # Server Location
      server: "opc.tcp://192.168.0.248:4840"

      # read-only
      current_pressure: "ns=1;s=SYNTHESISCONTROL_3:OPC_UA_SERVER.P_HPLC1.Value"
      # Control nodes (writable)
      flowrate: "ns=1;s=SYNTHESISCONTROL_3:OPC_UA_SERVER.HPLC_1_SETP.Value"
      pump_ON: "ns=1;s=SYNTHESISCONTROL_3:OPC_UA_SERVER.HPLC_1_ON.Value"
```

Figure S17: Device configuration in .yaml file.

5.2. Execution of experiments

Experimental procedures were implemented in the python control software, allowing for a wide variety of experiments to be performed. Parametrization was handled using a YAML serialization approach (Figure S18), offering an easily human-readable way to define and execute new experiments from a list of predefined procedures. This approach was taken to optimize the implementation of repeated procedures and increase the level of abstraction.

```yaml
flow_ramp1:
  description: "flow ramp experiment"
  procedure: "flow_ramp"
  parameters:
    reactor_volume: 4.23 #ml
    concentration_A: 0.3 #target concentration in mol/L
    equivalents_B: 1.2
    equivalents_C: 0.05
    temperature: 130.0 #°C
    start_tres: 2 #minutes
    experiment_duration: 60 #minutes
    slope_flowrate:  -0.0005 # ml/minute/second
```

Figure S18 Experiment configuration using .yaml format.

5.3. Data Processing

For each UHPLC measurement a .csv file was exported containing the absorbance values at a certain wavelength over the residence time. These UHPLC chromatogram data files were read by Peaxact ProcessLink and treated by a PEAXACT integration model. Concentration values were calculated from the calibration curves. The self-made python software was used to read the concentration values for all calibrated compounds from Peaxact ProcessLink and append those to a datafile. After each ramp experiment the dataset was written in the Dynochem model files. Therefore in a ‘scenarios’ sheet a line containing initial concentration values was appended and in the ‘data’ sheet a table with the residence time and concentrations of all calibrated species was added.

Data processing was performed using a Peaxact Integration model. UHPLC measurements were exported as single wavelength chromatograms in the .csv file format, which was then processed using the Peaxact software and read by the orchestration software. The associated OPCUA node of each datapoint is checked every second and upon detecting a change in the obtained result, the new datapoint is added to a data table containing all the raw data of each experiment. Each new data point is also processed automatically, obtaining the correct residence times and structure for use with Dynochem. Upon conclusion of the experiment, the processed data is automatically written to the appropriate Dynochem model files.

5.4. Automatic Kinetic Fitting

Once a new experiment is added to the Dynochem model files, RunScript Automation was used to automatically fit all of the parameters considered. After completion, the kinetic parameters are updated in the model file and the model selection criterion and sum of square values for each model were saved to track model performance over time and determine the most accurate model.

6. Automated Dynamic Experiments

The process setup is described in Section 2. All dynamic experiments were performed at constant temperatures and constant concentrations of starting materials while linearly reducing the flow rate with a slope of -0.05091 mL min^{-2}, starting at an initial flow rate of 2.354 mL min^{-1}, resulting in an initial residence time of 2 min for the 1st case study (Figure S19). For the 2nd case study and the 3rd case study the slope was set to -0.0289 mL min^{-2} with an initial flow rate of 1.33 mL min^{-1}, resulting in an initial residence time of 3.5 min. To equilibrate the reactor, the initial flow rate was held for 1.5 times the initial residence time. The total experimental duration after the equilibration was set to 60 min. After 80 % of the experimental time had passed, the total flow rate was pumped with solvent only to stay within the operating range of all pumps (Figure S19) and to pre-flush the reactor. The residence time during the ramps was calculated according to Equation 5 where v_0 is the initial flow rate in mL min^{-1}, α_v is the slope for the change of the flow rate in mL min^{-2}, V is the total volume in mL (reactor volume + dead volume to analytics for calculation of $t_{in,i}$; dead volume to analytics for calculation of $t_{L,i}$) and $t_{m,i}$ is the time passed from starting the flow rate in min.[4]

$$t_{L,i\ or\ In,i} = \frac{v_0 - \sqrt{v_0^2 - 2\alpha_v \cdot \left(v_0 \cdot t_{m,i} - 0.5\alpha_v t_{m,i}^2 - V\right)}}{\alpha_v} \quad (5)$$

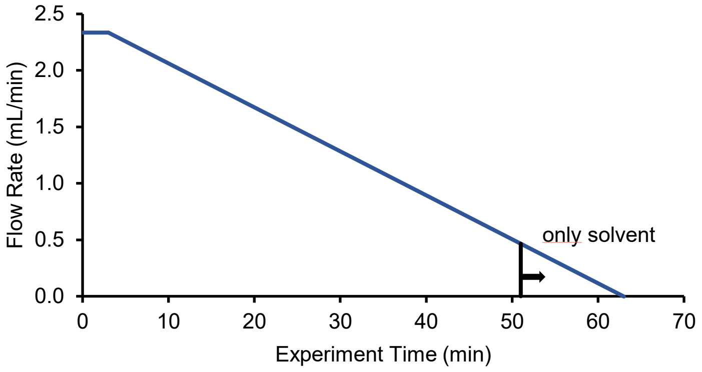

Figure S19. Experimental design of the flow ramps for the Buchwald Hartwig reaction.

6.1. Case Study 1: Buchwald Hartwig Reaction

Dynamic experiments according to Table S2 were performed for kinetic modeling across a residence time range from 2 minutes to 12 minutes. The experimental design alongside the UHPLC results are shown in **Figure S20**. The same set of experiments was performed for ArBr **1a** and ArBr **1b** as well.

Table S2. Experimental design for case study 1.

	Conc. 1 (mM)	Equiv. 2	Equiv. DBU	$Pd(OAc)_2$ loading (mol%)	Temperature (°C)
1	150	1.05	1.5	3.5	130
2	100	1.5	1.05	5.0	135
3	175	1.4	1.2	2.5	140
4	130	1.3	1.1	4.0	104
5	160	1.2	1.2	3.0	125
6	110	1.3	1.6	4.5	140
7	140	1.2	1.2	0.0	130

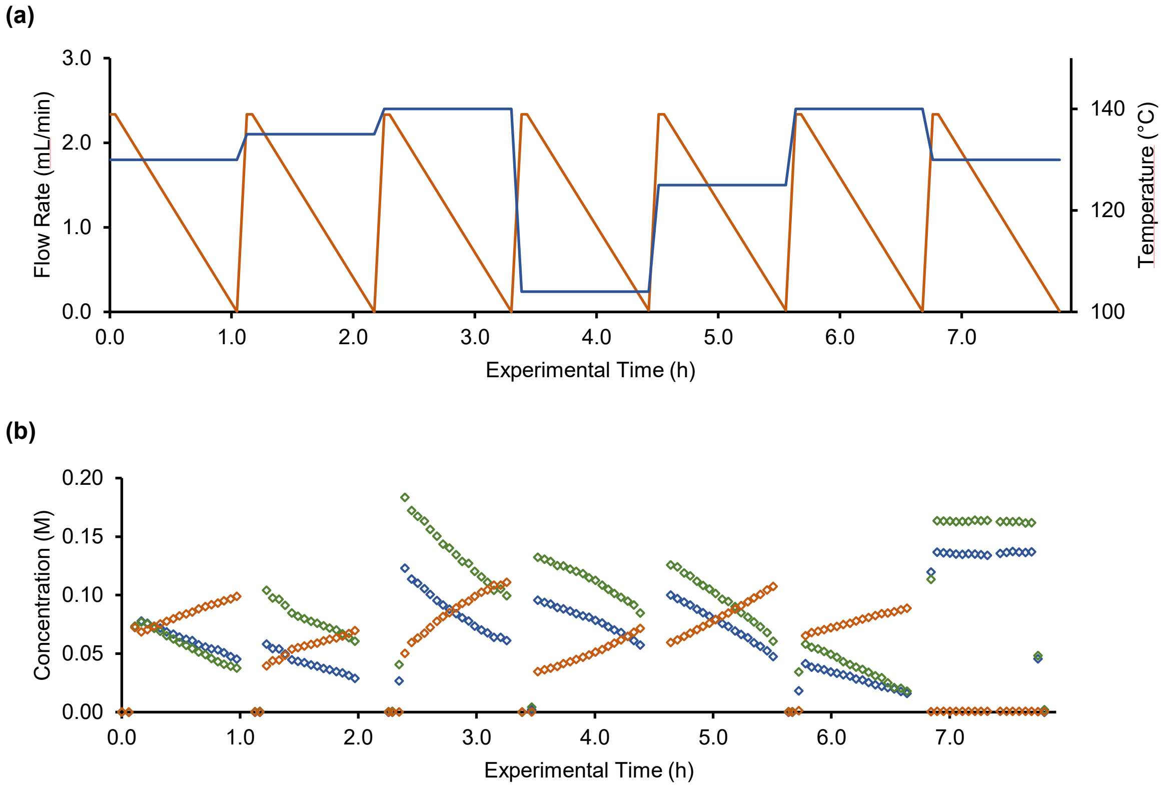

Figure S20. Experiments performed for kinetic fitting of the Buchwald Hartwig reaction. (a) total flow rate (orange) and temperature (blue), (b) UHPLC data for **1** (blue), **2** (green) and **3** (orange).

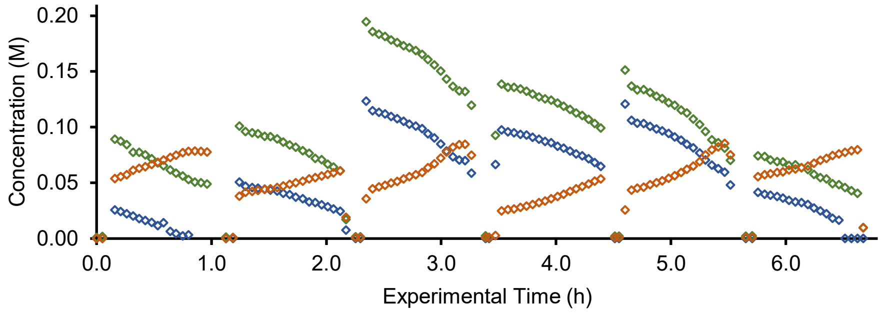

Figure S21. Experiments performed for kinetic fitting of the Buchwald Hartwig reaction with the methoxy substituted starting material. UHPLC data for **1a** (blue), **2** (green) and **3a** (orange).

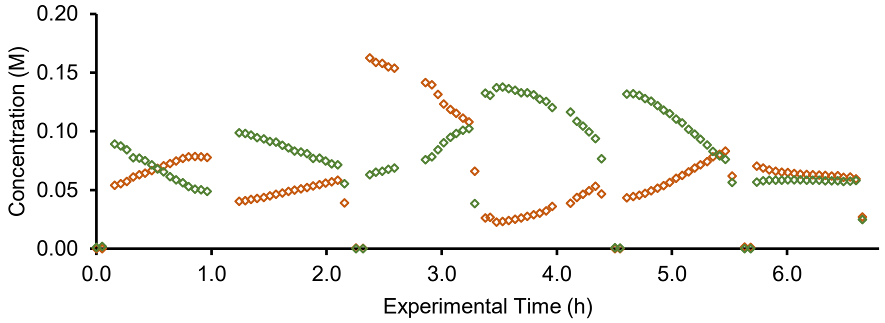

Figure S22. Experiments performed for kinetic fitting of the Buchwald Hartwig reaction with the CF_3 substituted starting material. UHPLC data for **2** (green) and **3b** (orange).

6.1. Case Study 2: Rhenium catalyzed thioanisole oxidation

Dynamic experiments according to Table S3 were performed for kinetic modeling of the rhenium catalyzed thioanisole oxidation across a residence time range from 3.5 minutes to 15 minutes. The experimental design together with the UHPLC results are shown in **Figure S23**. The same set of experiments was performed for thioanisole **4a** as well.

Table S3. Experimental design for case study 2.

	Conc. 4 (mM)	Equiv. NBu_4ClO_4	Re catalyst loading (mol%)	Temperature (°C)
1	120	0.25	0.5	85
2	110	0.55	0.75	75
3	90	0.65	1.0	65
4	105	0.70	0.6	55
5	95	0.45	0.7	70
6	130	0.30	0.4	80
7	100	0.40	0.0	85

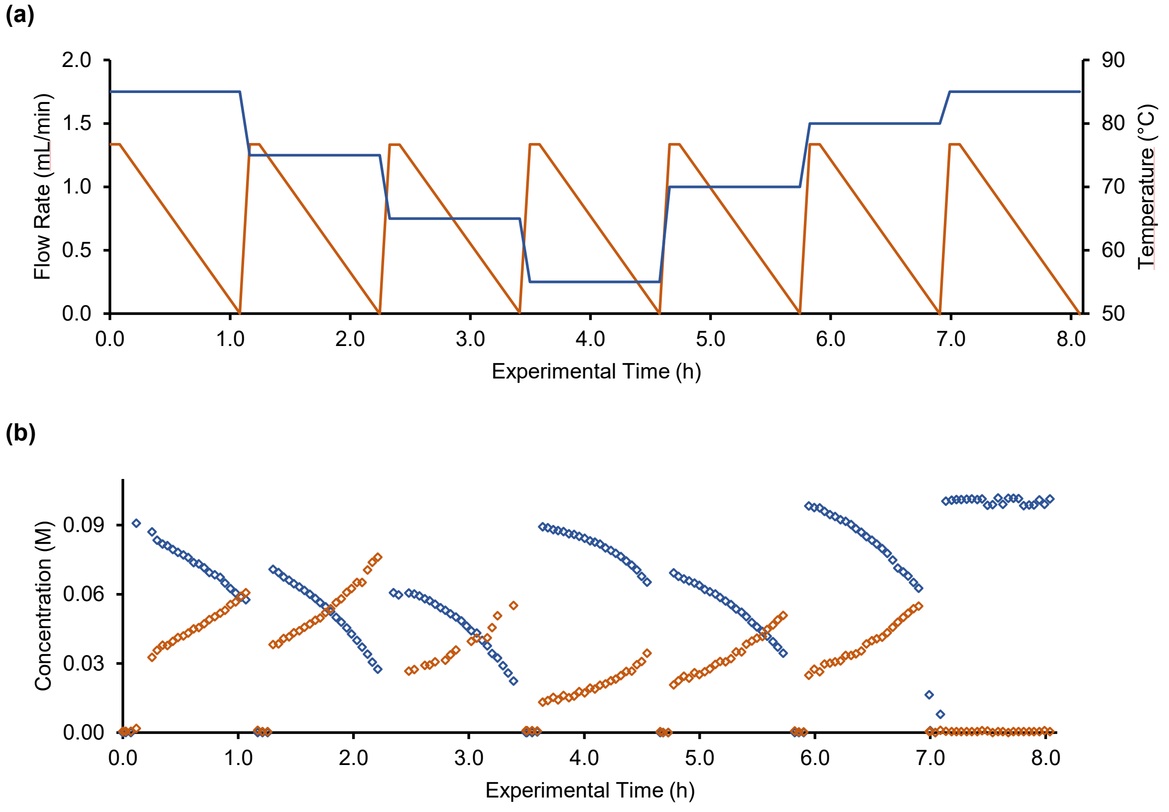

Figure S23. Experiments performed for kinetic fitting of rhenium catalyzed thioanisole oxidation. (a) total flow rate (orange) and temperature (blue), (b) UHPLC data for **4** (blue) and **5** (orange) Outliers for product (**5**) concentration from experiment 3 were removed.

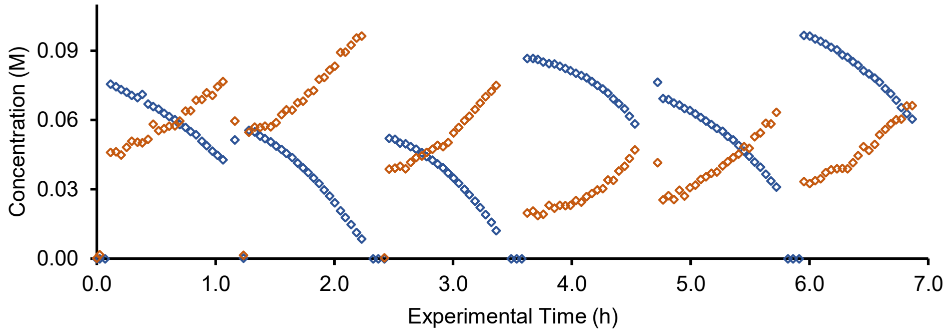

Figure S24. Experiments performed for kinetic fitting of rhenium catalyzed thioanisole oxidation. UHPLC data for **4a** (blue) and **5b** (orange).

6.2. Case Study 3: Copper catalyzed meta-selective coupling reaction

Dynamic experiments according to Table S4 were performed for kinetic modeling of the copper catalyzed meta-selective coupling reaction across a residence time range from 3.5 minutes to 15 minutes. The experimental design together with the UHPLC results are shown in **Figure S25**.

Table S4. Experimental design for case study 3.

	Conc. 6 (mM)	Equiv. 7	$Cu(OTf)_2$ loading (mol%)	Temperature (°C)
1	65	1.9	0	110
2	65	2.3	25	100
3	85	1.8	15	90
4	70	2.1	22	120
5	60	1.5	17.5	80
6	80	1.7	12	105
7	65	2.2	21	95

(a)

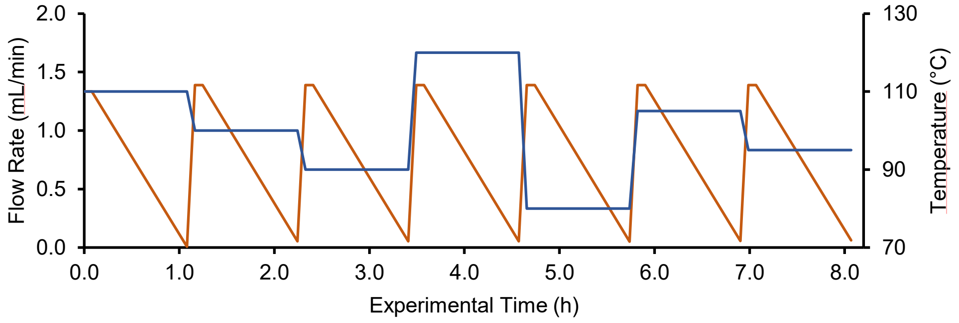

(b)

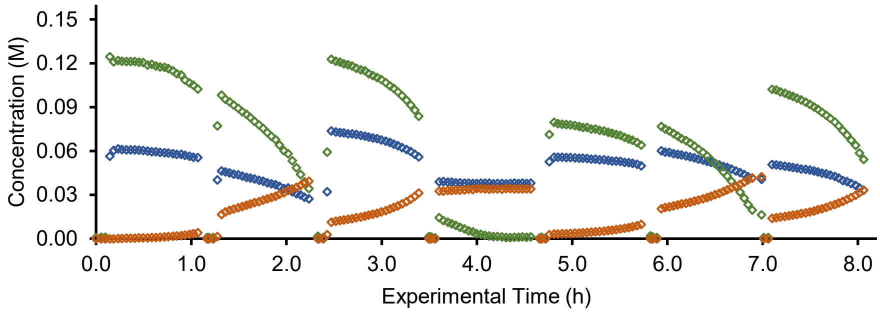

Figure S25. Experiments performed for kinetic fitting of copper catalyzed meta-selective coupling reaction. (a) total flow rate (orange) and temperature (blue), (b) UHPLC data for **6** (blue), **7** (green) and **8** (orange).

7. Automated Kinetic Fitting

For each case study a set of models was prepared based on literature and reasonable expected off-cycles. After a dynamic ramp experiment concluded, the dataset containing the UHPLC concentration data of all calibrated compounds was automatically associated with the calculated residence times for each point, converting the flow rates into reaction times and automatically added to the Dynochem file of each respective candidate model. To execute a fitting process Dynochem requires an entry in the ‘scenarios’ data sheet, which contains the initial parameters and an entry in the ‘data’ data sheet with all residence time dependent concentrations. All reactions were fitted as irreversible reactions. In cases where reversibility was considered, it was fitted by including the inverse reaction. Fitting results are given as k_{ref} (rate constant at a reference temperature (T_{ref})) and an activation energy (E_a) in J mol^{-1} (Equation 6). k is the rate constant (depending on the reaction order in s^{-1} or L mol^{-1} s^{-1}) at temperature T in K and R the ideal gas constant in J mol^{-1} K^{-1}.

$$k = k_{ref} \cdot e^{-\frac{E_a}{R} \cdot \left(\frac{1}{T} - \frac{1}{T_{ref}}\right)} \quad (6)$$

After the data was added to the model files, the models were automatically fitted using the RunScript Automation module provided by Scale-up systems. After the conclusion of each experiment, all models were fitted sequentially prior to the start of the next flow ramp experiment. Upon completion of the experimental run, the model with the best model selection criterion and sum of squares values was selected. This model was then used for further *in silico* reaction optimization.

The “model selection criterion” is an information theoretical metric provided by Dynochem to compare different models fit to the same data. A higher model selection criterion suggests a better model. More information on the model selection criterion can be found in the Dynochem documentation.

7.1. Case Study 1: Buchwald Hartwig Reaction

For case study 1 all possible combinations of the main catalytic cycle and the possible off-cycles as catalyst deactivation, dehalogenation (was divided into three partial reactions) and thiophene decomposition (Scheme S4) were fitted.[5][6]

Fitting results for all rate constants and activation energies at a reference temperature of 130 °C are given for the best fit model 1 in Table S5. This best fit model includes the main catalytic cycle and all possible off-cycle reactions. Parity plots for compounds **1**, **2** and **3** are given for all models in Figure S26 to Figure S33. A comparison of all fitted models is given in Table S6.

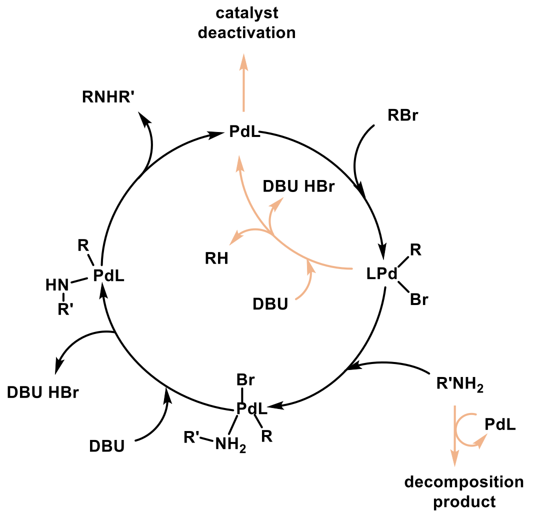

Scheme S4. Catalytic cycle including side reactions used for kinetic modeling of the Buchwald Hartwig reaction. All possible combinations of the main catalytic cycle and possible off-cycles of catalyst deactivation, dehalogenation and thiophene decomposition were fitted.

The best fit model (model 1) included the main catalytic cycle and all possible off-cycle reactions (catalyst deactivation, dehalogenation of the aryl bromide and thiophene decomposition). The fitted rate constants and activation energies are given in Table S5 and the according parity plots are shown in Figure S26.

Table S5. Model 1 Buchwald Hartwig reaction: Reaction network and fitted parameters for kinetic modeling in Dynochem.

1	PdL + **1b** → PdInt1	k = 80.7 ± 3.5 L $mol^{-1}s^{-1}$	E_a = 142 ± 3 kJ mol^{-1}
2	PdInt1 + **2** → PdInt2	k = 10.0 ± 1.3 L $mol^{-1}s^{-1}$	E_a = 103 ± 12 kJ mol^{-1}
3	PdInt2 + DBU → PdInt3 + DBU·HBr	k = 1.31 ± 0.09 L $mol^{-1}s^{-1}$	E_a = 20.1 ± 6.4 kJ mol^{-1}
4	PdInt3 → **3b** + PdL	k = 0.308 ± 0.035 s^{-1}	E_a = 27.2 kJ mol^{-1} (low sensitivity)
5	PdL → PdLinactive	k = 0.348 s^{-1} (low sensitivity)	E_a = 117 kJ mol^{-1} (low sensitivity)
6	PdInt1 + DBU → PdIntSide	k = 1.80 L $mol^{-1}s^{-1}$ (low sensitivity)	E_a = 163 ± 13 kJ mol^{-1}
7	PdIntSide → PdDBUBr + nitrobenzene	k = 93.1 s^{-1} (low sensitivity)	E_a = 51.0 kJ mol^{-1} (low sensitivity)
8	PdDBUBr → PdL + DBUBr	k = 54.6 s^{-1} (low sensitivity)	E_a = 67.9 kJ mol^{-1} (low sensitivity)
9	**2** + PdL → decomposition product + PdL	k = 15.2 ± 0.4 L $mol^{-1}s^{-1}$	E_a = 124 ± 4 kJ mol^{-1}

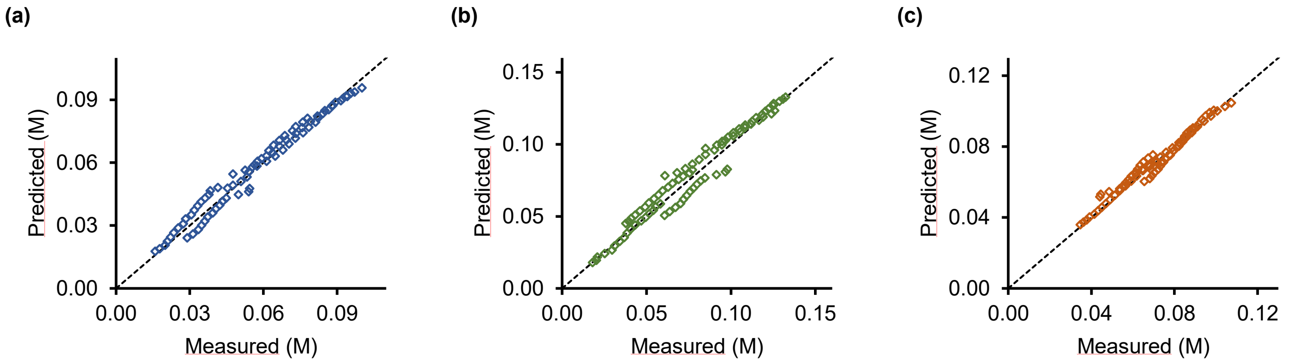

Figure S26. Parity Plots for model 1 for the Buchwald Hartwig reaction. (a) **1**, (b) **2**, (c) **3**.

Model 2 includes only the main catalytic cycle.

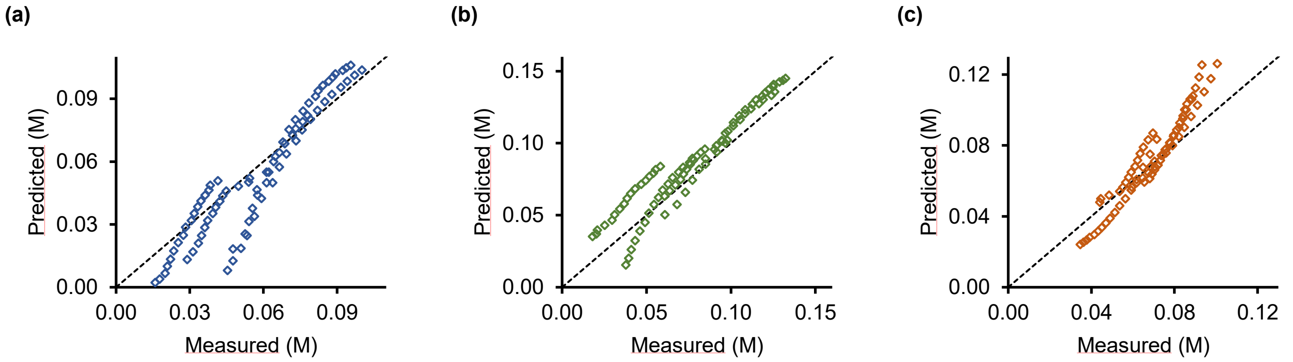

Figure S27. Parity Plots for model 2 for the Buchwald Hartwig reaction. (a) **1**, (b) **2**, (c) **3**.

Model 3 includes the main catalytic cycle and catalyst deactivation.

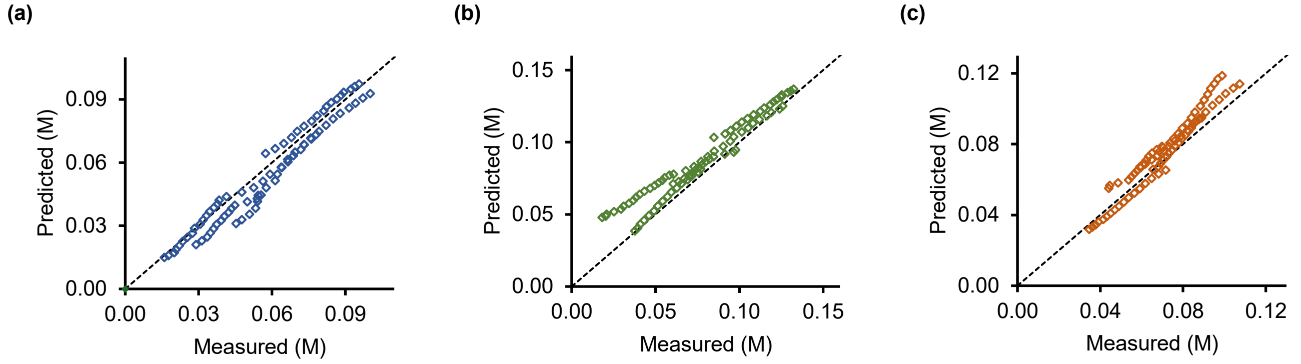

Figure S28. Parity Plots for model 3 for the Buchwald Hartwig reaction. (a) **1**, (b) **2**, (c) **3**.

Model 4 includes the main catalytic cycle and dehalogenation of the aryl bromide.

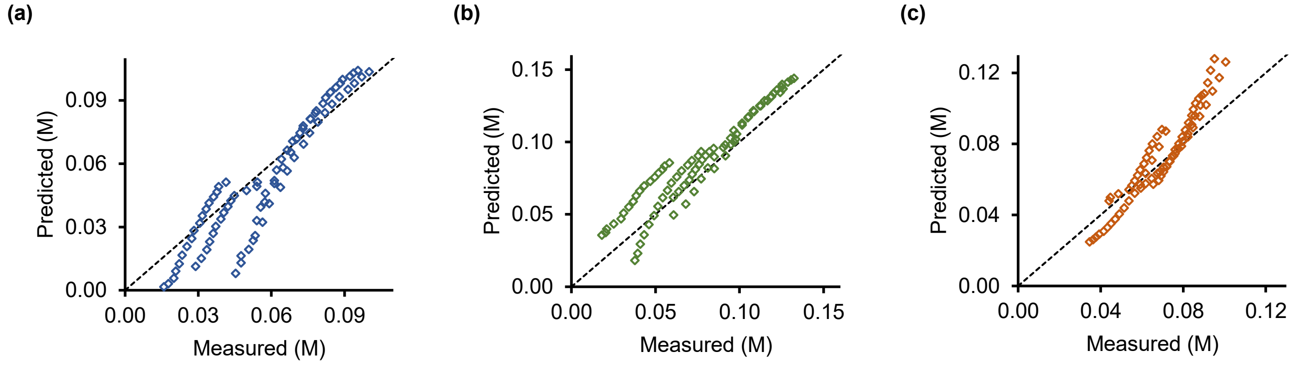

Figure S29. Parity Plots for model 4 for the Buchwald Hartwig reaction. (a) **1**, (b) **2**, (c) **3**.

Model 5 includes the main catalytic cycle and palladium catalyzed thiophene decomposition.

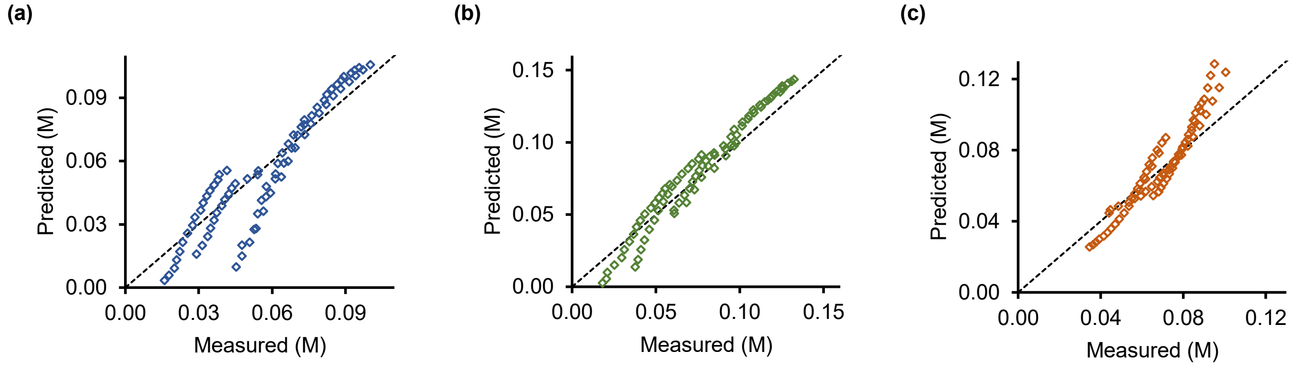

Figure S30. Parity Plots for model 4 for the Buchwald Hartwig reaction. (a) **1**, (b) **2**, (c) **3**.

Model 6 includes the main catalytic cycle, catalyst deactivation and dehalogenation of the aryl bromide.

(a) **(b)** **(c)**

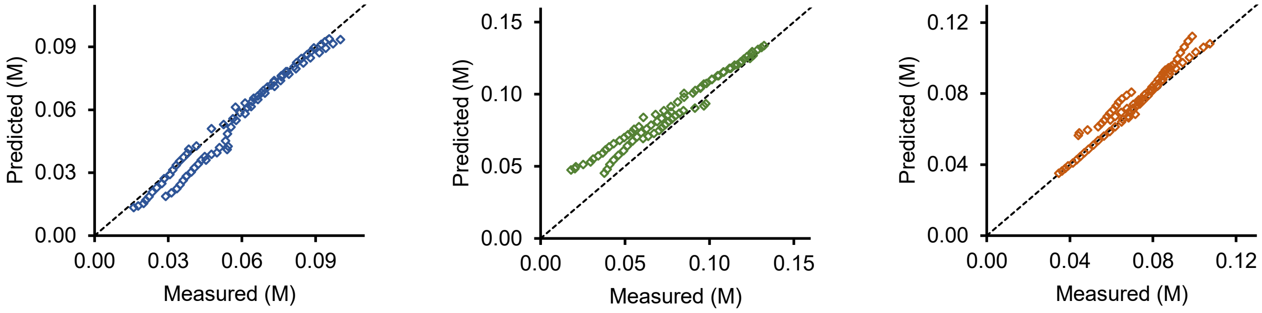

Figure S31. Parity Plots for model 6 for the Buchwald Hartwig reaction. (a) **1**, (b) **2**, (c) **3**.

Model 7 includes the main catalytic cycle, catalyst deactivation and palladium catalyzed thiophene decomposition.

(a) **(b)** **(c)**

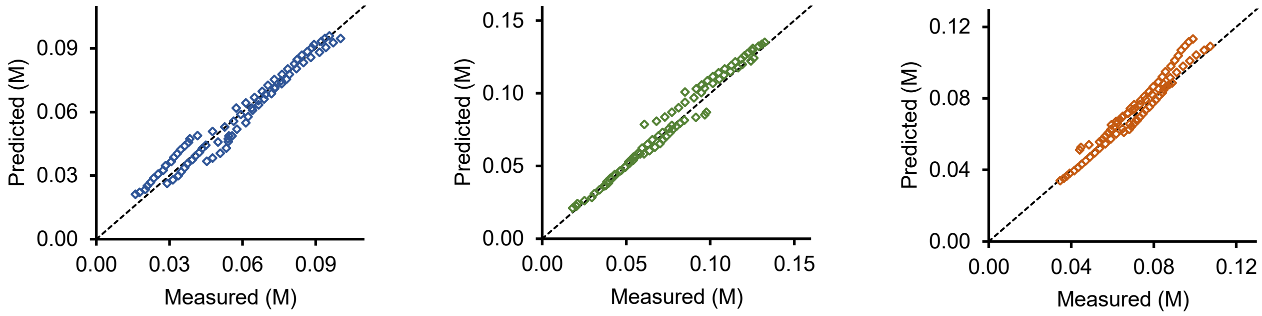

Figure S32. Parity Plots for model 7 for the Buchwald Hartwig reaction. (a) **1**, (b) **2**, (c) **3**.

Model 8 includes the main catalytic cycle, dehalogenation of the aryl bromide and palladium catalyzed thiophene decomposition.

(a) **(b)**

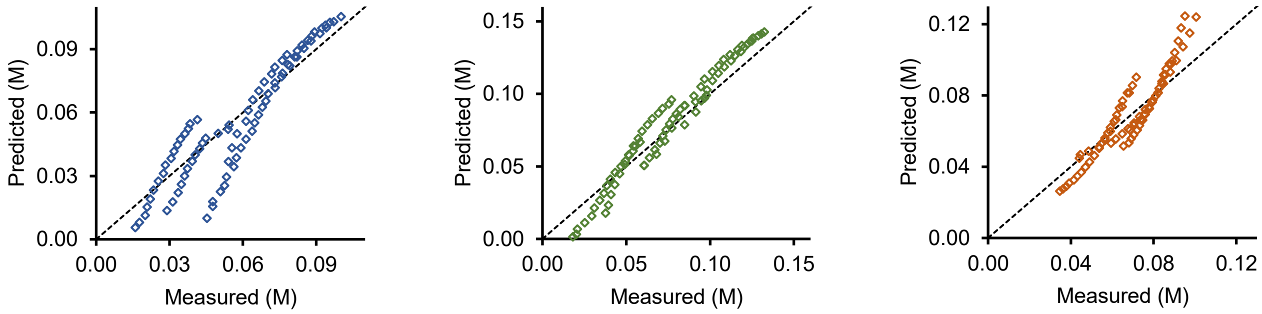

(c)

Figure S33. Parity Plots for model 8 for the Buchwald Hartwig reaction. (a) **1**, (b) **2**, (c) **3**.

Table S6. Model Comparison Buchwald Hartwig reaction. For Dynochem model selection criterion, AIC/n and BIC/n higher is better, for MAPD, SSQ and RMSE of validations, lower is better. model selection criterion, AIC and BIC are dimensionless. AIC and BIC were normalized using the number of datapoints used in the model fitting process, n = 324.

	model selection criterion	Akaike information criterion (AIC)	Bayesian information criterion (BIC)	mean absolute percentage error	SSQ	RMSE of product 3 for validation experiments
Model 1	4.30	8.74	8.82	9.96 %	4.62 mM²	11.1 mM
Model 2	2.76	6.23	6.25	39.14 %	66.6 mM²	15.9 mM
Model 3	3.43	6.30	3.33	35.53 %	63.9 mM²	14.2 mM
Model 4	2.46	7.65	7.69	21.23 %	12.0 mM²	14.8 mM
Model 5	3.07	6.29	6.32	36.62 %	64.3 mM²	12.8 mM
Model 6	3.04	8.05	8.12	15.80 %	8.09 mM²	15.5 mM
Model 7	2.40	6.38	6.42	30.04 %	60.6 mM²	10.6 mM
Model 8	2.87	8.01	8.07	17.04 %	8.33 mM²	11.0 mM

As seen in **Table S6**, Model 1 performs best on all metrics investigated, with the highest model selection criterion, AIC and BIC and the lowest values on the error metrics. The difference between the models is quite significant, even between the best (model 1) and the second-best (model 6) models when considering ΔAIC/n (0.69). As these numbers are normalized by the number of datapoints (n = 324), the threshold for ΔAIC/n is 0.031 to obtain strong evidence that one model significantly outperforms the other.
Corrected AIC was also investigated, but in this case, due to the large sample size it collapsed to AIC.

For aryl bromides **1a** and **1b** the fitting results for model 1, including all possible off-cycle reactions, are given in Table S7 and Table S8. The parity plots are shown in Figure S34 and Figure S35. For the reaction with the trifluoromethyl substituted starting material the kinetic model was fitted using concentrations of **2** and **3b** as significant decomposition of **1b** was observed.

A kinetic comparison of the substrates **1**, **1a** and **1b** shows significant differences in the rate constants. For aryl bromide **1** and methoxy substituted aryl bromide **1a** oxidative addition is slower than reductive elimination, whereas for the CF_3-substituted aryl bromide **1b** this is opposite and reductive elimination is slower than oxidative addition. This behavior aligns with the overall reactivity trend that the strongly electron-withdrawing CF_3 group activates the aryl bromide and increases the rate of oxidative addition. In contrast, the electron-donating methoxy group slows down the overall reaction.[7]

Table S7. Model 4 Buchwald Hartwig reaction with methoxy substituted starting material **1a**: Reaction network and fitted parameters for kinetic modeling in Dynochem.

1	PdL + **1a** → PdInt1	k = 3.46 ± 0.74 L $mol^{-1}s^{-1}$	E_a = 48.9 ± 3.7 kJ mol^{-1}
2	PdInt1 + **2** → PdInt2	k = 1560 ± 150 L $mol^{-1}s^{-1}$	E_a = 27.3 kJ mol^{-1} (low sensitivity)
3	PdInt2 + DBU → PdInt3 + DBU·HBr	k = 0.627 ± 0.045 L $mol^{-1}s^{-1}$	E_a = 18.1 ± 5.7 kJ mol^{-1}
4	PdInt3 → **3a** + PdL	k = 366 s^{-1} (low sensitivity)	E_a = 48.4 kJ mol^{-1} (low sensitivity)
5	PdL → PdLinactive	k = 0.0111 ± 0.0025 s^{-1}	E_a = 0 kJ mol^{-1} (at min)
6	PdInt1 + DBU → PdIntSide	k = 267 L $mol^{-1}s^{-1}$ (low sensitivity)	E_a = 70.5 ± 17 kJ mol^{-1}
7	PdIntSide → PdDBUBr + nitrobenzene	k = 569 s^{-1} (low sensitivity)	E_a = 73.5 kJ mol^{-1} (low sensitivity)
8	PdDBUBr → PdL + DBUBr	k = 552 s^{-1} (low sensitivity)	E_a = 73.8 kJ mol^{-1} (low sensitivity)
9	**2** + PdL → decomposition product + PdL	k = 0.604 ± 0.131 L $mol^{-1}s^{-1}$	E_a = 48.3 ± 11 kJ mol^{-1}

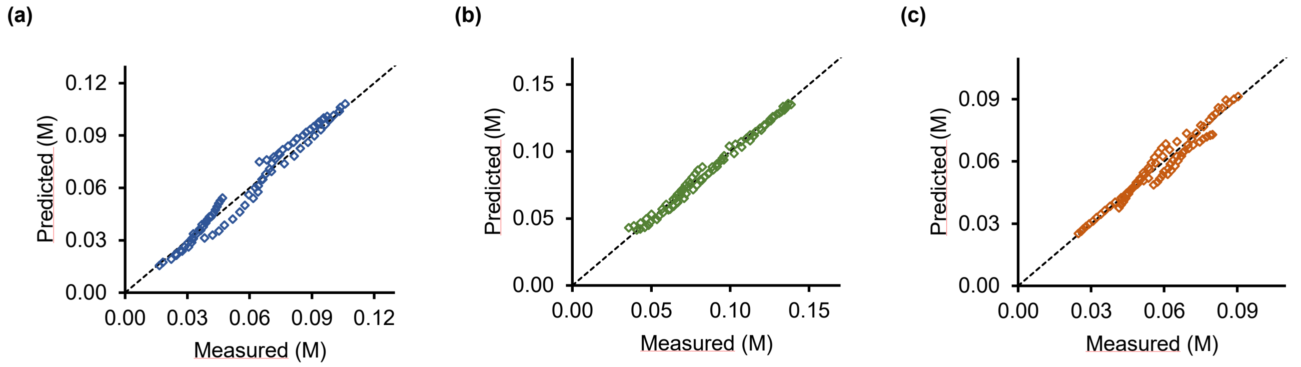

Figure S34. Parity Plots for model 4 for the Buchwald Hartwig reaction with methoxy substituted starting material **1a**. (a) **1a**, (b) **2**, (c) **3a**.

Table S8. Model 4 Buchwald Hartwig reaction with trifluoromethyl substituted starting material **1b**: Reaction network and fitted parameters for kinetic modeling in Dynochem.

1	PdL + **1b** → PdInt1	k = 80.7 ± 3.5 L $mol^{-1}s^{-1}$	E_a = 142 ± 3 kJ mol^{-1}
2	PdInt1 + **2** → PdInt2	k = 10.0 ± 1.3 L $mol^{-1}s^{-1}$	E_a = 103 ± 12 kJ mol^{-1}
3	PdInt2 + DBU → PdInt3 + DBU·HBr	k = 1.31 ± 0.09 L $mol^{-1}s^{-1}$	E_a = 20.1 ± 6.4 kJ mol^{-1}
4	PdInt3 → **3b** + PdL	k = 0.308 ± 0.035 s^{-1}	E_a = 27.2 kJ mol^{-1} (low sensitivity)
5	PdL → PdLinactive	k = 0.348 s^{-1} (low sensitivity)	E_a = 117 kJ mol^{-1} (low sensitivity)
6	PdInt1 + DBU → PdIntSide	k = 1.80 L $mol^{-1}s^{-1}$ (low sensitivity)	E_a = 163 ± 13 kJ mol^{-1}
7	PdIntSide → PdDBUBr + nitrobenzene	k = 93.1 s^{-1} (low sensitivity)	E_a = 51.0 kJ mol^{-1} (low sensitivity)
8	PdDBUBr → PdL + DBUBr	k = 54.6 s^{-1} (low sensitivity)	E_a = 67.9 kJ mol^{-1} (low sensitivity)
9	**2** + PdL → decomposition product + PdL	k = 15.2 ± 0.4 L $mol^{-1}s^{-1}$	E_a = 124 ± 4 kJ mol^{-1}

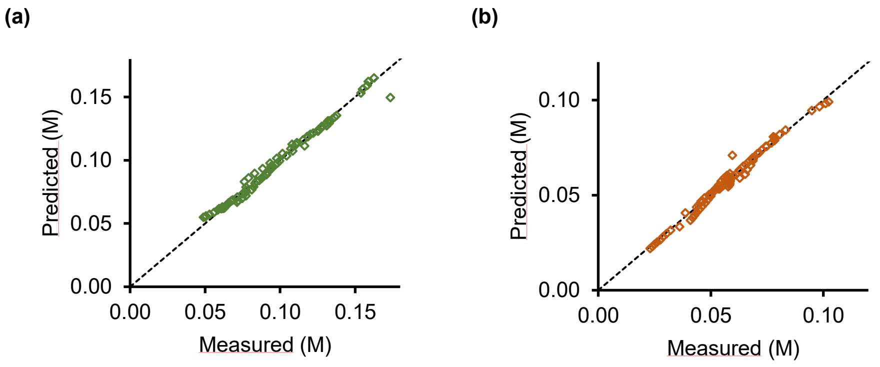

Figure S35. Parity Plots for model 4 for the Buchwald Hartwig reaction with trifluoromethyl substituted starting material **1b**. (a) **2**, (b) **3b**.

7.2. Case Study 2: Rhenium catalyzed thioanisole oxidation

Five different candidate models were proposed for the rhenium catalyzed thioanisole oxidation based on previous kinetic investigations including the main reaction pathway and all possible variations of inclusion of reversible/irreversible coordination of chloride as catalyst deactivation and activation of an inactive Re(VII) species (Scheme S5).
Kinetic modeling shows that inclusion of catalyst inhibition through reversible chloride coordination significantly improves the model fit. The inclusion of the activation of an inactive rhenium(VII) species in model 3 results in a very low reaction rate and does not improve the model quality . Therefore model 2 and 3 result in similar reaction rates for all steps with a similar model selection criterion of 3.64 (Table S10). Model 2 was used for further *in silico* optimization as further complication as in model 3 does not improve the model. Rate constants (fitted at a reference temperature of 85 °C) and activation energies for model 2 are shown in Table S9. The parity plots for **4** and **5** are shown in Figure S36 to Figure S40.

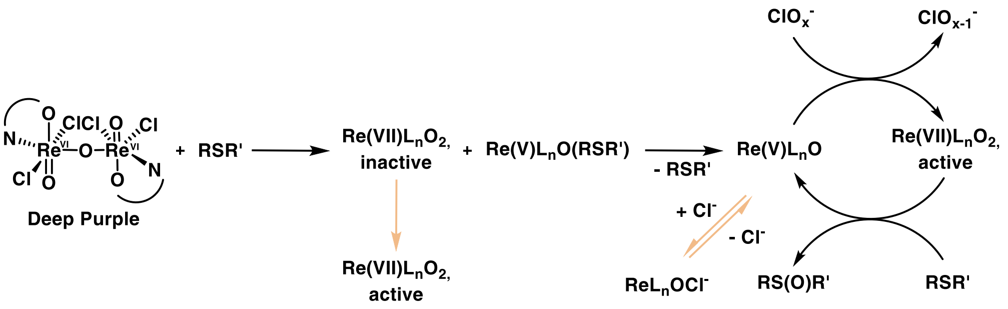

Scheme S5. Reaction scheme used for kinetic modeling of the rhenium catalyzed thioanisole oxidation.

Model 1 includes only the main reaction pathway. The parity plots are shown in Figure S36.

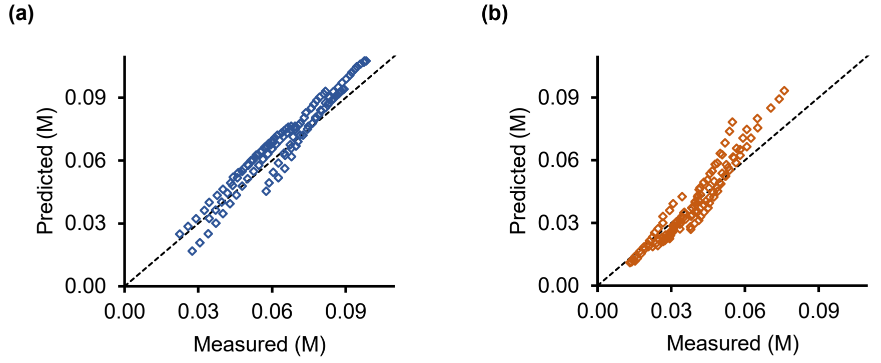

Figure S36. Parity Plots for model 1 for the rhenium catalyzed thioanisole oxidation. (a) 4-bromothioanisole (**4**), (b) product (**5**).

Model 2 includes the main reaction pathway and a reversible catalyst inhibition *via* chloride coordination. The parity plots are shown in Figure S37 and the rate constants and activation energies in Table S9.

Table S9. Model 2: Rhenium catalyzed thioanisole oxidation: Reaction network and fitted parameters for kinetic modeling in Dynochem.

1	DP + **4** → Re(V)bromothioanisole + Re(VII)inactive	k = 1000 L mol^{-1}s^{-1} (low sensitivity)	E_a = 19.1 kJ mol^{-1} (low sensitivity)
2	Re(V)bromothioanisole → Re(V) + **4**	k = 2633 s^{-1} (low sensitivity)	E_a = 19.1 kJ mol^{-1} (low sensitivity)
3	Re(V) + perchlorate → Re(VII) + chlorate	k = 28.0 ± 7.2 L mol^{-1}s^{-1}	E_a = 68.5 ± 1.9 kJ mol^{-1}
4	Re(V) + chlorate → Re(VII) + chlorite	k = 153 L mol^{-1}s^{-1} (low sensitivity)	E_a = 60.6 kJ mol^{-1}
5	Re(V) + chlorite → Re(VII) + hypochlorite	k = 3863 L mol^{-1}s^{-1} (low sensitivity)	E_a = 0 kJ mol^{-1} (low sensitivity)
6	Re(V) + hypochlorite → Re(VII) + chloride	k = 5124 L mol^{-1}s^{-1} (low sensitivity)	E_a = 0 kJ mol^{-1} (low sensitivity)
7	Re(VII) + **4** → **5** + Re(V)	k = 402 L mol^{-1}s^{-1} (low sensitivity)	E_a = 169 ± 4 kJ mol^{-1} (low sensitivity)
8	Re(V) + chloride → Re(V)Cl	k = 3006 L mol^{-1}s^{-1} (low sensitivity)	E_a = 15.3 kJ mol^{-1} (low sensitivity)
9	Re(V)Cl → Re(V) + chloride	k = 1.15 ± 0.32 L s^{-1}	E_a = 12.5 kJ mol^{-1} (low sensitivity)

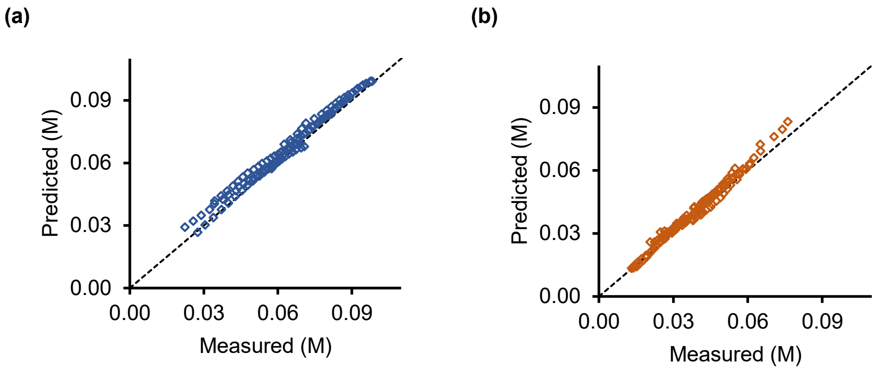

Figure S37. Parity Plots for model 2 for the rhenium catalyzed thioanisole oxidation. (a) 4-bromothioanisole (**4**), (b) product (**5**).

Model 3 includes the main reaction pathway, a reversible catalyst inhibition *via* chloride coordination and activation of a Re(VII) species. The parity plots are shown in Figure S38.

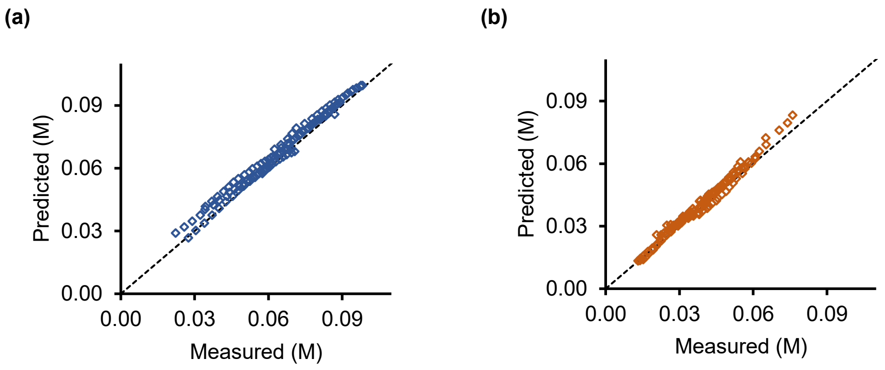

Figure S38. Parity Plots for model 3 for the rhenium catalyzed thioanisole oxidation. (a) 4-bromothioanisole (**4**), (b) product (**5**).

Model 4 includes the main reaction pathway, an irreversible catalyst inhibition *via* chloride coordination and activation of a Re(VII) species. The parity plots are shown in Figure S39.

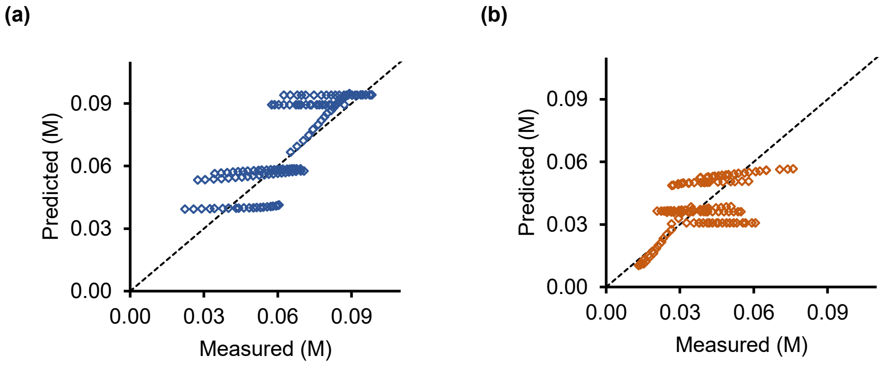

Figure S39. Parity Plots for model 4 for the rhenium catalyzed thioanisole oxidation. (a) 4-bromothioanisole (**4**), (b) product (**5**).

Model 5 includes the main reaction pathway and an irreversible catalyst inhibition *via* chloride coordination. The parity plots are shown in Figure S40.

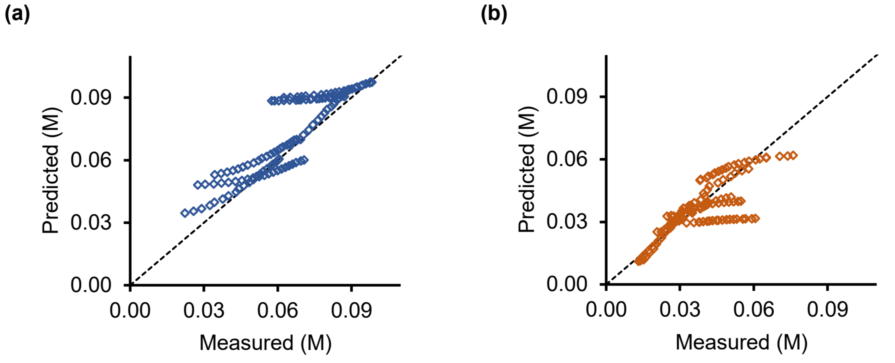

Figure S40. Parity Plots for model 5 for the rhenium catalyzed thioanisole oxidation. (a) 4-bromothioanisole (**4**), (b) product (**5**).

Table S10. Model Comparison: Rhenium catalyzed thioanisole oxidation.

	Model Selection Criterion	SSQ	RMSE of product 5 for validation experiments
Model 1	2.19	1.92 mM²	12.6
Model 2	3.64	0.37 mM²	7.45
Model 3	3.65	0.45 mM²	7.16
Model 4	1.05	6.95 mM²	24.1
Model 5	1.51	4.09 mM²	24.7

The rhenium catalyzed thioanisole oxidation was performed with unsubstituted thioanisole (**4a**) as well, resulting in a slower overall reaction rate than for 4-bromothioanisole (**4**), primarily resulting from a slower rate constant for the oxygen atom transfer from Re(VII) to the thioether forming sulfoxide **5a**. The fitted rate constants and activation energies for model 2 are given in Table S11 and the parity plots for **4a** and **5a** are shown in Figure S41. Experiments 2-6 were used for kinetic fitting.

Table S11. Model 2: Rhenium catalyzed thioanisole oxidation for unsubstituted thioanisole **4a**: Reaction network and fitted parameters for kinetic modeling in Dynochem.

1	DP + **4a** → Re(V)bromothioanisole + Re(VII)inactive	k = 1000 L $mol^{-1}s^{-1}$ (low sensitivity)	E_a = 21.6 kJ mol^{-1} (low sensitivity)
2	Re(V)bromothioanisole → Re(V) + **4a**	k = 2633 s^{-1} (low sensitivity)	E_a = 29.0 kJ mol^{-1} (low sensitivity)
3	Re(V) + perchlorate → Re(VII) + chlorate	k = 40.0 L $mol^{-1}s^{-1}$ (low sensitivity)	E_a = 26.9 kJ mol^{-1} (low sensitivity)
4	Re(V) + chlorate → Re(VII) + chlorite	k = 124 L $mol^{-1}s^{-1}$ (low sensitivity)	E_a = 30.0 kJ mol^{-1} (low sensitivity)
5	Re(V) + chlorite → Re(VII) + hypochlorite	k = 3859 L $mol^{-1}s^{-1}$ (low sensitivity)	E_a = 17.6 kJ mol^{-1} (low sensitivity)
6	Re(V) + hypochlorite → Re(VII) + chloride	k = 5120 L $mol^{-1}s^{-1}$ (low sensitivity)	E_a = 17.0 kJ mol^{-1} (low sensitivity)
7	Re(VII) + **4a** → **5a** + Re(V)	k = 20.4 L $mol^{-1}s^{-1}$ (low sensitivity)	E_a = 85.6 kJ mol^{-1} (low sensitivity)
8	Re(V) + chloride → Re(V)Cl	k = 3006 L $mol^{-1}s^{-1}$ (low sensitivity)	E_a = 16.1 kJ mol^{-1} (low sensitivity)
9	Re(V)Cl → Re(V) + chloride	k = 2.07 s^{-1} (low sensitivity)	E_a = 20.5 kJ mol^{-1} (low sensitivity)

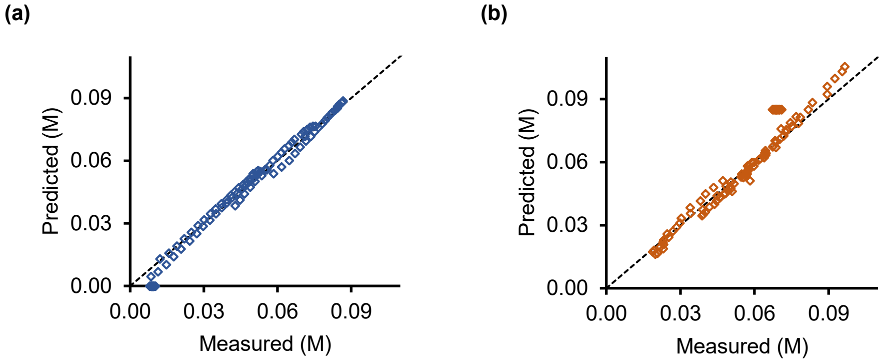

Figure S41. Parity Plots for model 2 for the rhenium catalyzed thioanisole oxidation using unsubstituted thioanisole **4a** as substrate. (a) 4-bromothioanisole (**4a**), (b) product (**5a**).

7.3. Case Study 3: Copper catalyzed meta-selective coupling reaction

For kinetic modeling of the copper catalyzed meta-selective coupling reaction 16 different candidate models were fitted. As shown in Scheme S6 the models are constructed from one of two possible catalytic cycles[8],[9] alongside the decomposition of **7** under copper catalyzed and thermal conditions as well as product formation under thermal conditions.

All rate constants are given at a reference temperature of 100 °C. The fitted rate constants and activation energies for model 1 are given in Table S12. Parity plots are shown in Figure S42 to Figure S57. The model comparison (Table S13) indicates that model 1 and model 16 are the best fitting models with a model selection criterion of 3.80. This means that the inclusion of both a copper-catalyzed decomposition pathway and a thermal product formation gives the best model fit. Separation of amide coordination followed by deprotonation does not further improve the model quality compared to fitting it as a single reaction step as the comparison of models 1 and 16 indicates. Therefore model 1 was used for further *in silico* optimization.

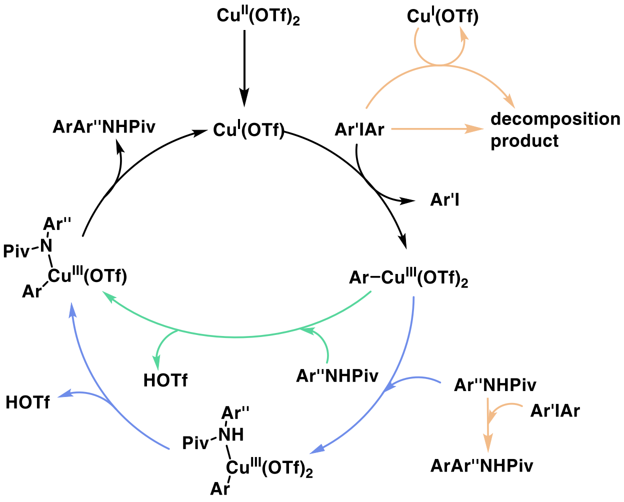

Scheme S6. Catalytic cycle used for kinetic modeling of the copper catalyzed meta-selective coupling reaction. Model 1: violet dot, model 2: green dot, model 3: blue dot, model 4: red dot.

Model 1 was selected as the best fit model and includes the catalytic cycle 1 and the three off-cycles, uncatalyzed product formation, copper catalyzed diaryliodonium triflate (**7**) decomposition and uncatalyzed decomposition of **7**. Table S12 includes all fitted rate constants and activation energies and the parity plots are shown in Figure S42.

Table S12. Model 1: Copper catalyzed meta-selective coupling reaction: Reaction network and fitted parameters for kinetic modeling in Dynochem.

1	Cu(II)(OTf)$_2$ → Cu(I)OTf + OTf$^-$	k = 296 s^{-1} (low sensitivity)	E$_a$ = 0 kJ mol^{-1} (low sensitivity)
2	Cu(I)(OTf) + **7** → ArCu(III)(OTf)$_2$ + MesI	k = 0.0477 ± 0.0005 L mol^{-1}s^{-1}	E$_a$ = 80.1 ± 0.9 kJ mol^{-1} (low sensitivity)
3	ArCu(III)(OTf)$_2$ + **6** → ArCu(III)OTf(amide) + TfOH	k = 159 L mol^{-1}s^{-1} (low sensitivity)	E$_a$ = 2.1 kJ mol^{-1} (low sensitivity)
4	ArCu(III)OTf(amide) → **8** + Cu(I)OTf	k = 199 s^{-1} (low sensitivity)	E$_a$ = 13.2 kJ mol^{-1} (low sensitivity)
5	**7** → decomposition product	k = 7.2E-6 s^{-1} (low sensitivity)	E$_a$ = 323 ± 23 kJ mol^{-1}
6	**7** + Cu(I)OTf → decomposition product + Cu(I)OTf	k = 0.100 ± 0.002 L mol^{-1}s^{-1}	E$_a$ = 98.3 ± 4.0 kJ mol^{-1}
7	**6** + **7** → **8** + MesI + PhOTf	k = 0.00045 ± 0.00002 L mol^{-1}s^{-1}	E$_a$ = 0 kJ mol^{-1} (low sensitivity)

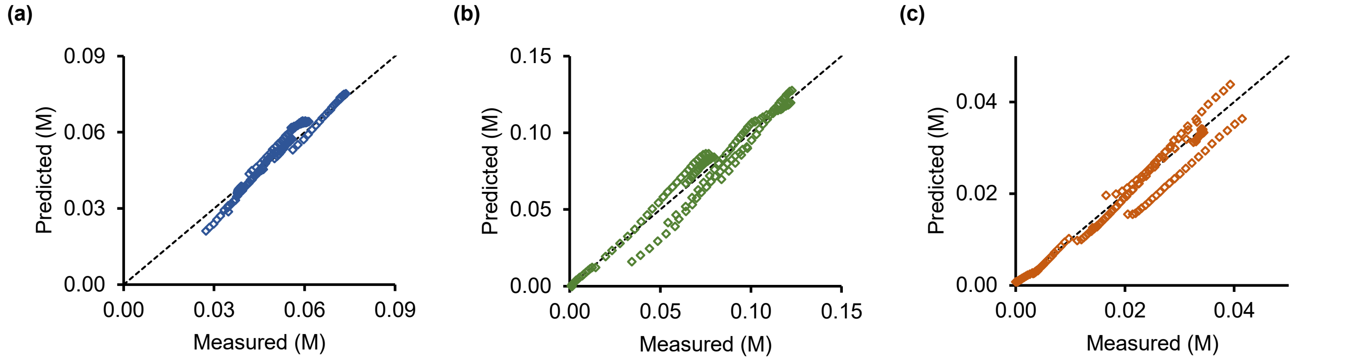

Figure S42. Parity Plots for model 1 for the copper catalyzed meta-selective coupling reaction. (a) *N*-(*o*-tolyl)pivalamide (**6**), (b) mesityl(phenyl)iodonium triflate (**7**), (c) product (**8**).

Model 2 includes catalytic cycle 1 without any off-cycles. Parity plots are shown in Figure S43.

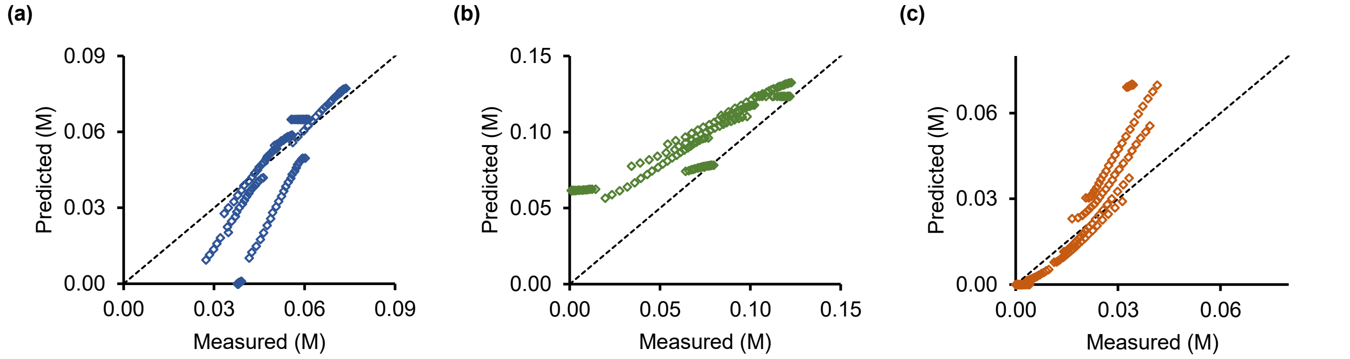

Figure S43. Parity Plots for model 2 for the copper catalyzed meta-selective coupling reaction. (a) *N*-(*o*-tolyl)pivalamide (**6**), (b) mesityl(phenyl)iodonium triflate (**7**), (c) product (**8**).

Model 3 includes catalytic cycle 1 and the uncatalyzed decomposition of **7**. Parity plots are shown in Figure S44.

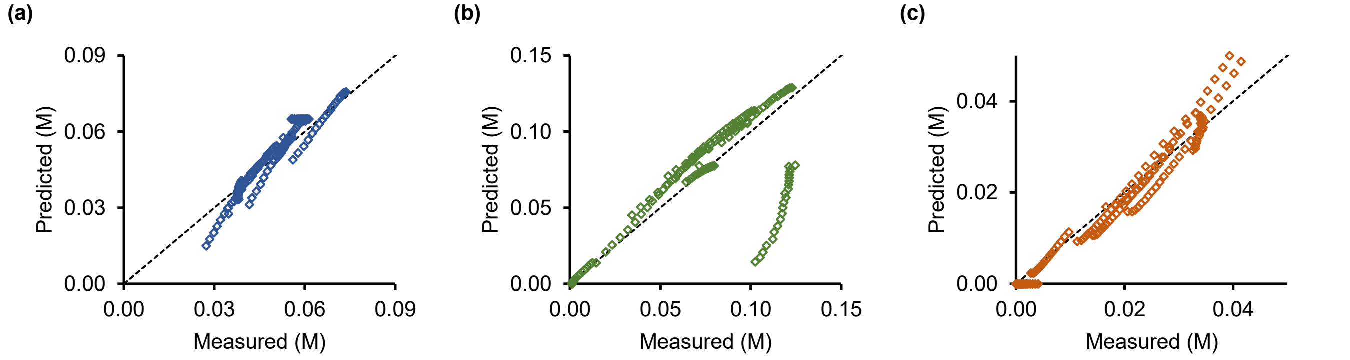

Figure S44. Parity Plots for model 3 for the copper catalyzed meta-selective coupling reaction. (a) *N*-(*o*-tolyl)pivalamide (**6**), (b) mesityl(phenyl)iodonium triflate (**7**), (c) product (**8**).

Model 4 includes catalytic cycle 1 and the copper catalyzed decomposition of **7**. Parity plots are shown in Figure S45.

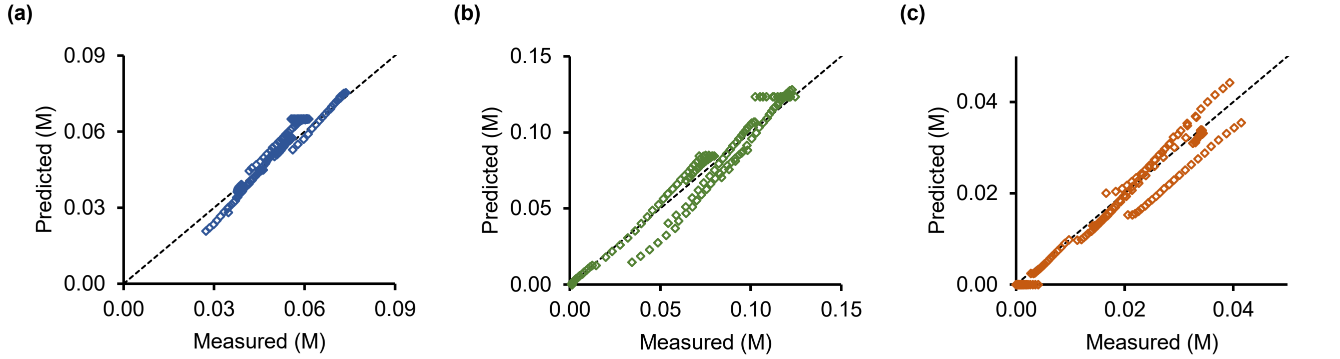

Figure S45. Parity Plots for model 4 for the copper catalyzed meta-selective coupling reaction. (a) *N*-(*o*-tolyl)pivalamide (**6**), (b) mesityl(phenyl)iodonium triflate (**7**), (c) product (**8**).

Model 5 includes catalytic cycle 1 and the uncatalyzed product formation. Parity plots are shown in Figure S46.

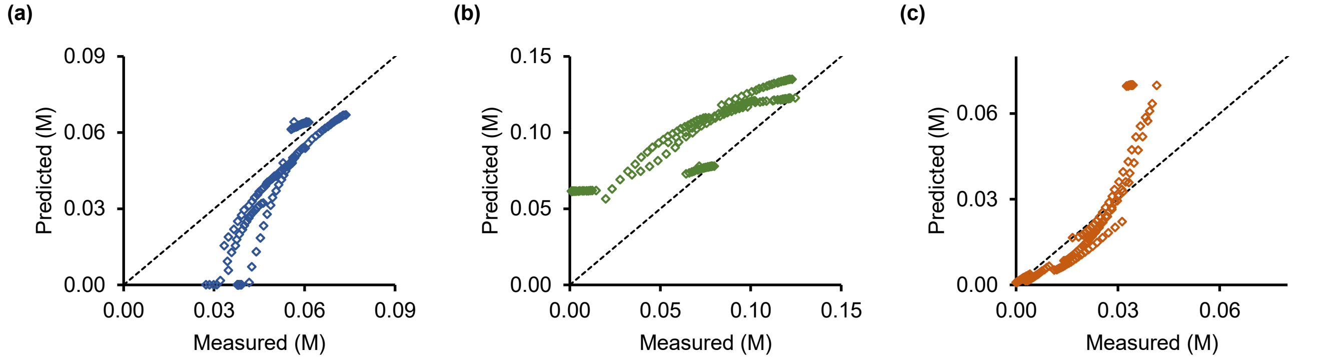

Figure S46. Parity Plots for model 5 for the copper catalyzed meta-selective coupling reaction. (a) *N*-(*o*-tolyl)pivalamide (**6**), (b) mesityl(phenyl)iodonium triflate (**7**), (c) product (**8**).

Model 6 includes catalytic cycle 1, the copper catalyzed decomposition of **7** and the uncatalyzed decomposition of **7**. Parity plots are shown in Figure S47.

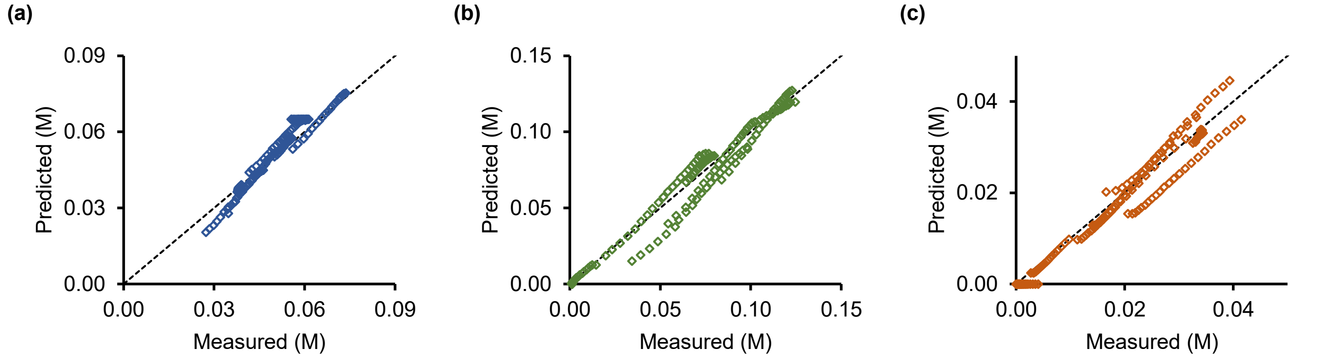

Figure S47. Parity Plots for model 6 for the copper catalyzed meta-selective coupling reaction. (a) *N*-(*o*-tolyl)pivalamide (**6**), (b) mesityl(phenyl)iodonium triflate (**7**), (c) product (**8**).

Model 7 includes catalytic cycle 1, the uncatalyzed decomposition of **7** and the uncatalyzed product formation. Parity plots are shown in Figure S48.

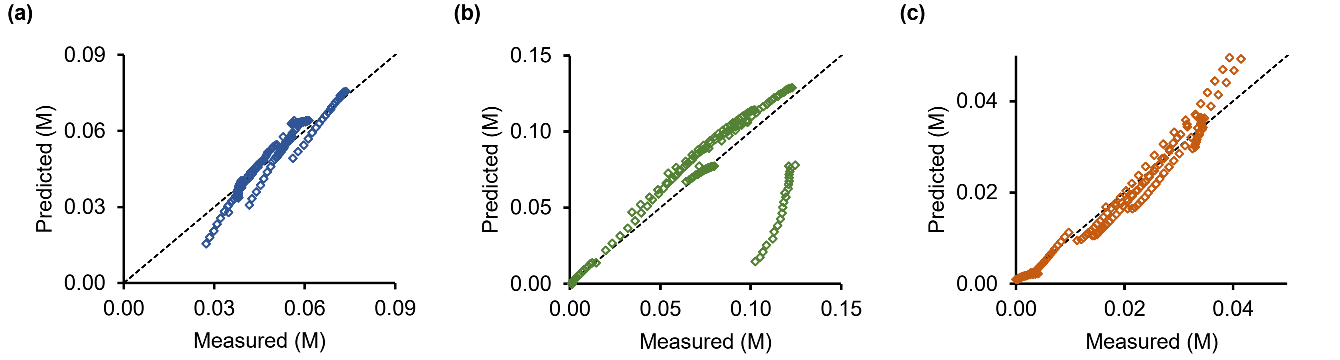

Figure S48. Parity Plots for model 7 for the copper catalyzed meta-selective coupling reaction. (a) *N*-(*o*-tolyl)pivalamide (**6**), (b) mesityl(phenyl)iodonium triflate (**7**), (c) product (**8**).

Model 8 includes catalytic cycle 1, the copper catalyzed decomposition of **7** and the uncatalyzed product formation. Parity plots are shown in Figure S49.

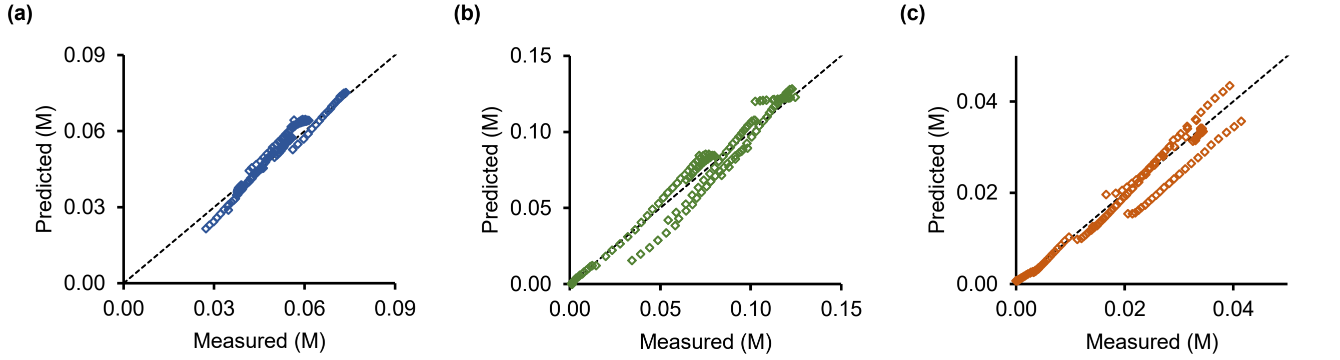

Figure S49. Parity Plots for model 8 for the copper catalyzed meta-selective coupling reaction. (a) *N*-(*o*-tolyl)pivalamide (**6**), (b) mesityl(phenyl)iodonium triflate (**7**), (c) product (**8**).

Model 9 includes catalytic cycle 2 without any off-cycles. Parity plots are shown in Figure S50.

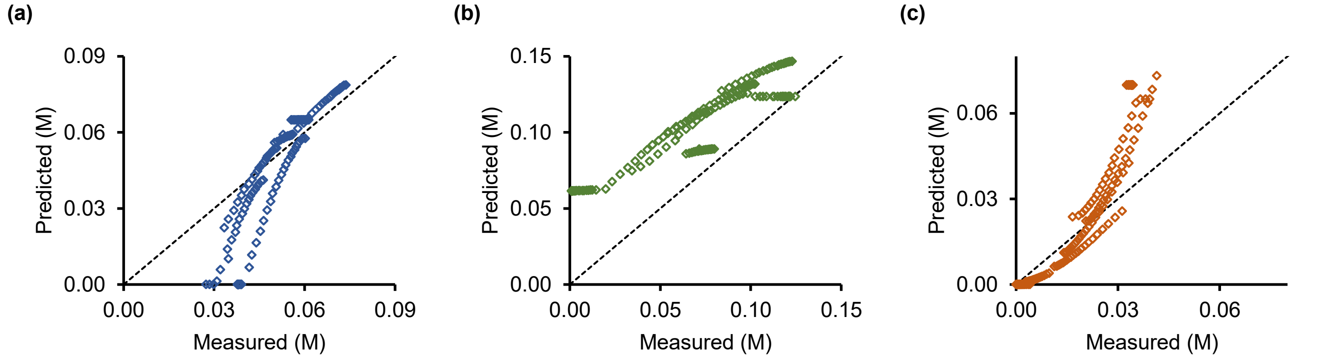

Figure S50. Parity Plots for model 9 for the copper catalyzed meta-selective coupling reaction. (a) *N*-(*o*-tolyl)pivalamide (**6**), (b) mesityl(phenyl)iodonium triflate (**7**), (c) product (**8**).

Model 10 includes catalytic cycle 2 and the uncatalyzed decomposition of **7**. Parity plots are shown in Figure S51.

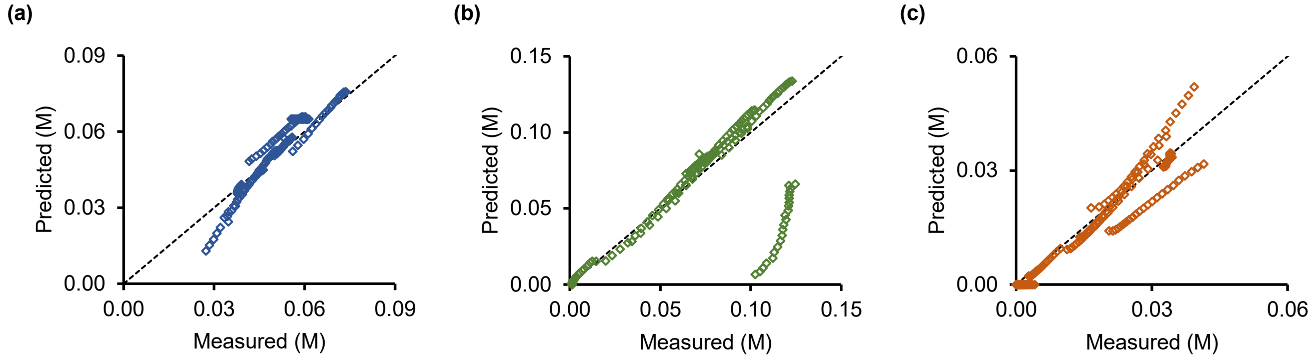

Figure S51. Parity Plots for model 10 for the copper catalyzed meta-selective coupling reaction. (a) *N*-(*o*-tolyl)pivalamide (**6**), (b) mesityl(phenyl)iodonium triflate (**7**), (c) product (**8**).

Model 11 includes catalytic cycle 2 and the copper catalyzed decomposition of **7**. Parity plots are shown in Figure S52.

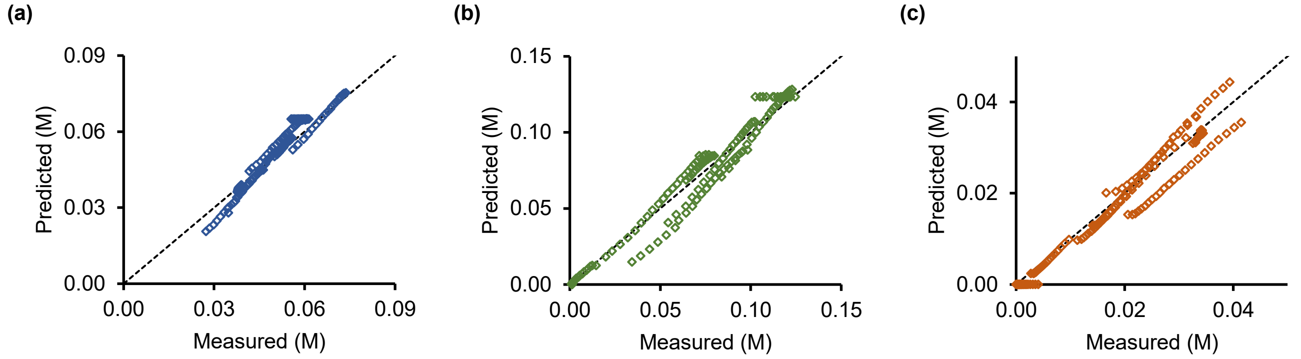

Figure S52. Parity Plots for model 11 for the copper catalyzed meta-selective coupling reaction. (a) *N*-(*o*-tolyl)pivalamide (**6**), (b) mesityl(phenyl)iodonium triflate (**7**), (c) product (**8**).

Model 12 includes catalytic cycle 2 and the uncatalyzed product formation. Parity plots are shown in Figure S53.

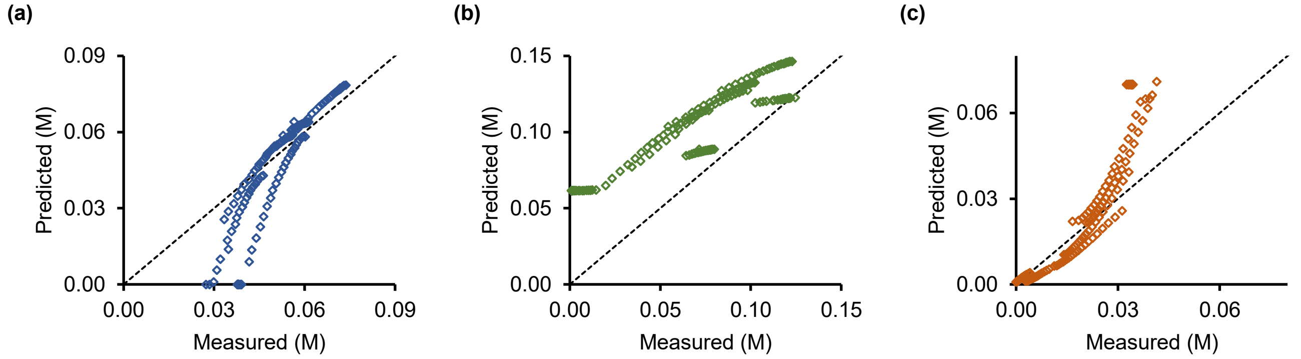

Figure S53. Parity Plots for model 12 for the copper catalyzed meta-selective coupling reaction. (a) *N*-(*o*-tolyl)pivalamide (**6**), (b) mesityl(phenyl)iodonium triflate (**7**), (c) product (**8**).

Model 13 includes catalytic cycle 2, the uncatalyzed decomposition of **7** and the copper catalyzed decomposition of **7**. Parity plots are shown in Figure S54.

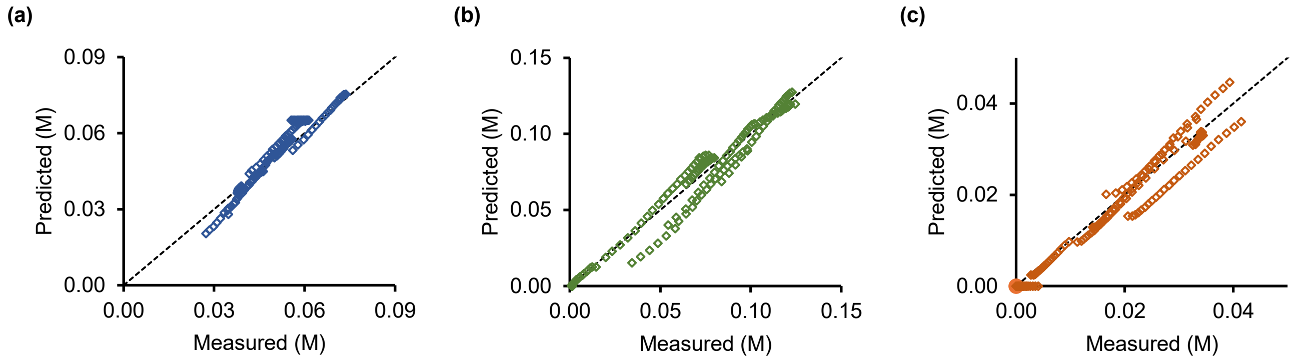

Figure S54. Parity Plots for model 13 for the copper catalyzed meta-selective coupling reaction. (a) *N*-(*o*-tolyl)pivalamide (**6**), (b) mesityl(phenyl)iodonium triflate (**7**), (c) product (**8**).

Model 14 includes catalytic cycle 2, the uncatalyzed decomposition of **7** and the uncatalyzed product formation. Parity plots are shown in Figure S55.

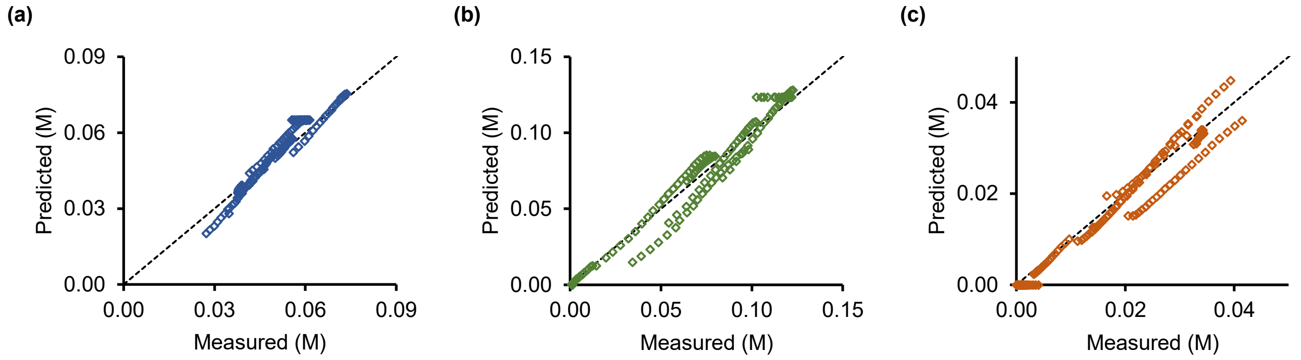

Figure S55. Parity Plots for model 14 for the copper catalyzed meta-selective coupling reaction. (a) *N*-(*o*-tolyl)pivalamide (**6**), (b) mesityl(phenyl)iodonium triflate (**7**), (c) product (**8**).

Model 15 includes catalytic cycle 2, the copper catalyzed decomposition of **7** and the uncatalyzed product formation. Parity plots are shown in Figure S56.

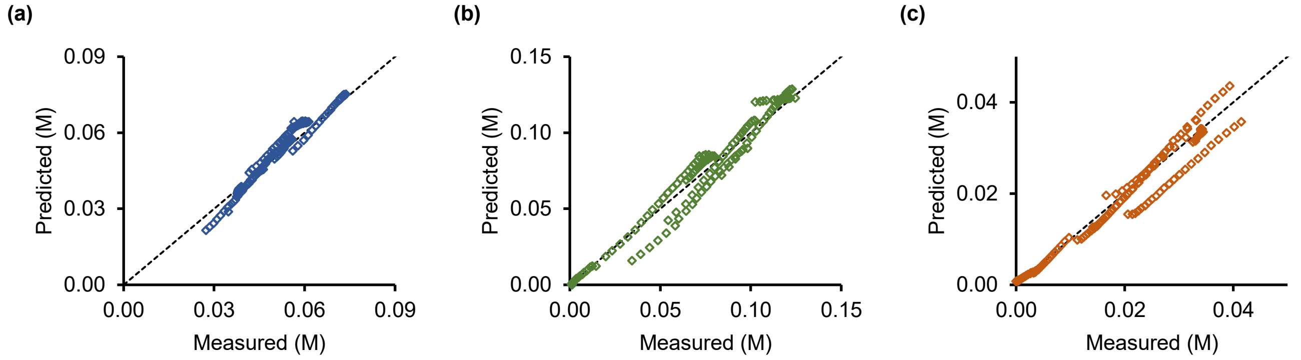

Figure S56. Parity Plots for model 15 for the copper catalyzed meta-selective coupling reaction. (a) *N*-(*o*-tolyl)pivalamide (**6**), (b) mesityl(phenyl)iodonium triflate (**7**), (c) product (**8**).

Model 16 includes catalytic cycle 2, the uncatalyzed decomposition of **7**, the copper catalyzed decomposition of **7** and the uncatalyzed product formation. Parity plots are shown in Figure S57.

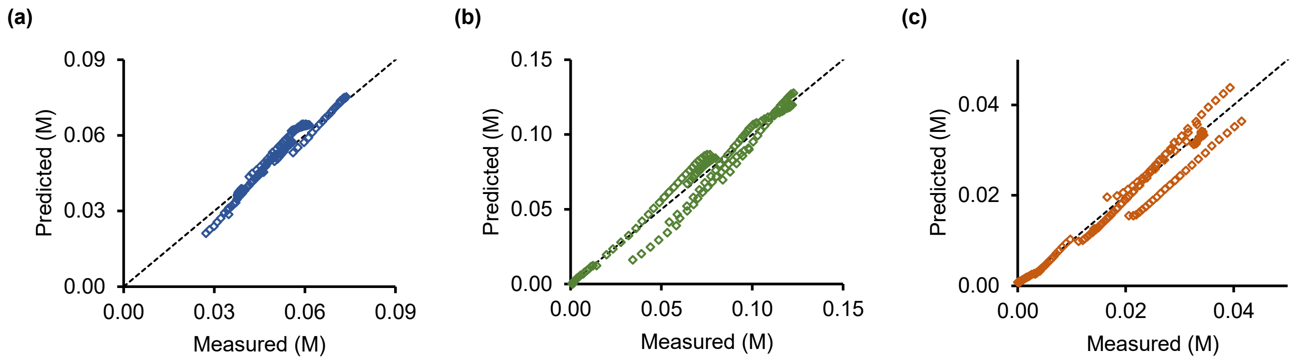

Figure S57. Parity Plots for model 16 for the copper catalyzed meta-selective coupling reaction. (a) *N*-(*o*-tolyl)pivalamide (**6**), (b) mesityl(phenyl)iodonium triflate (**7**), (c) product (**8**).

Table S13. Model Comparison: Copper catalyzed meta-selective coupling reaction.

	Model Selection Criterion	SSQ	RMSE of product 8 for validation experiments
Model 1	3.80	2.78 mM²	2.22
Model 2	0.76	436 mM²	27.7
Model 3	1.40	15.0 mM²	13.6
Model 4	3.49	7.19 mM²	2.43
Model 5	0.62	438 mM²	28.6
Model 6	3.62	7.05 mM²	2.22
Model 7	1.39	11.5 mM²	13.5
Model 8	3.65	2.86 mM²	2.51
Model 9	0.46	449 mM²	29.8
Model 10	1.14	16.6 mM²	6.29
Model 11	3.53	6.42 mM²	2.42
Model 12	0.45	405 mM²	28.5
Model 13	3.66	6.29 mM²	2.21
Model 14	3.54	7.17 mM²	2.28
Model 15	3.70	2.45 mM²	2.48
Model 16	3.80	2.78 mM²	2.22

8. *In Silico* Optimization

The selected model for each case study was used for multi-objective optimization. To carry out self-optimization a Bayesian optimization approach was utilized. The optimization algorithm was initialized using a random sample (20 queries) from the kinetic model. To perform multi-objective optimization the qEHVI algorithm (as implemented in BOTorch) was selected as a greedy multi-objective optimization algorithm. All data was normalized prior to passing it to the optimization algorithm. The code for this implementation is provided alongside the Supporting Information.

8.1. Case Study 1: Buchwald Hartwig Reaction

The objectives for this optimization were to maximize yield and productivity, while minimizing catalyst loading using parameters from model 4 (Table S5).
The boundaries of the variables were defined with:

- temperature: 80 – 140°C
- concentration 2-bromonitrobenzene (**1**): 80 – 160 mM
- equivalents 2-amino-5-methyl-3-thiophenecarbonitrile (**2**): 1.0 – 2.0
- equivalents DBU: 1.0 – 2.0
- catalyst loading: 3.0 – 7.0 mol%
- residence time: 2 – 15 min

All points of the optimization are shown in Table S14.

Table S14. Results multi-objective optimization of the Buchwald Hartwig reaction. Entries highlighted in green were performed experimentally for model validation.

T (°C)	c(bromo) (mM)	equiv. (thiophene)	equiv. DBU	Pd loading (mol%)	t_{res} (s)	yield (%)	Pd result	Productivity	Pareto Optimal
140.0	160.0	1.73	1.79	0.053	120	76.2	-0.053	3.66	TRUE
140.0	160.0	1.79	1.87	0.060	176	88.0	-0.060	2.90	TRUE
140.0	160.0	1.78	1.82	0.044	120	70.3	-0.044	3.38	TRUE
140.0	160.0	1.89	1.97	0.051	298	90.8	-0.051	1.76	TRUE
140.0	160.0	1.86	1.92	0.044	209	82.3	-0.044	2.27	TRUE
140.0	160.0	1.72	1.76	0.038	120	64.6	-0.038	3.10	TRUE
140.0	160.0	1.74	1.78	0.035	212	74.8	-0.035	2.04	TRUE
140.0	160.0	1.80	1.82	0.067	281	94.5	-0.067	1.94	TRUE
140.0	160.0	1.41	1.92	0.059	120	77.7	-0.059	3.73	TRUE
140.0	160.0	1.97	1.84	0.037	332	84.4	-0.037	1.46	TRUE
140.0	160.0	2.00	1.00	0.030	120	57.3	-0.030	2.75	TRUE
140.0	160.0	2.00	1.00	0.070	120	86.4	-0.070	4.15	TRUE
140.0	160.0	1.46	1.41	0.030	247	71.9	-0.030	1.68	TRUE
140.0	160.0	1.48	1.44	0.030	169	63.8	-0.030	2.18	TRUE
140.0	160.0	1.69	1.66	0.068	274	94.6	-0.068	2.00	TRUE
140.0	160.0	1.63	1.37	0.030	291	75.5	-0.030	1.49	TRUE
140.0	149.3	1.56	1.36	0.030	209	66.5	-0.030	1.72	TRUE
140.0	160.0	2.00	1.00	0.030	900	84.3	-0.030	0.54	TRUE
84.9	115.4	1.95	1.72	0.034	606	31.2	-0.034	0.21	FALSE

82.7	134.7	1.72	1.71	0.050	708	47.0	-0.050	0.32	FALSE
99.8	127.9	1.85	1.06	0.038	151	29.5	-0.038	0.90	FALSE
82.2	93.2	1.96	1.85	0.045	405	27.6	-0.045	0.23	FALSE
131.4	104.7	1.13	1.76	0.036	270	49.8	-0.036	0.70	FALSE
121.0	128.2	1.10	1.16	0.034	591	60.1	-0.034	0.47	FALSE
121.5	122.1	1.32	1.32	0.057	503	77.6	-0.057	0.68	FALSE
114.3	143.0	1.53	1.10	0.043	763	71.8	-0.043	0.48	FALSE
109.1	146.6	1.41	1.43	0.053	756	74.6	-0.053	0.52	FALSE
83.9	98.3	1.60	1.05	0.066	407	40.5	-0.066	0.35	FALSE
102.8	141.4	1.57	1.96	0.056	357	62.2	-0.056	0.89	FALSE
94.1	125.3	1.90	1.30	0.070	779	68.3	-0.070	0.40	FALSE
107.2	117.9	1.43	1.24	0.044	127	31.5	-0.044	1.06	FALSE
92.8	99.1	1.50	1.60	0.035	185	19.9	-0.035	0.39	FALSE
105.2	86.9	1.40	1.56	0.035	639	38.0	-0.035	0.19	FALSE
110.3	99.9	1.67	1.63	0.059	867	67.4	-0.059	0.28	FALSE
84.0	125.1	1.09	1.63	0.070	230	36.5	-0.070	0.71	FALSE
94.6	109.9	1.99	1.79	0.056	213	38.3	-0.056	0.71	FALSE
89.7	94.8	1.18	1.90	0.040	129	14.3	-0.040	0.38	FALSE
80.0	141.3	1.86	1.30	0.052	136	22.8	-0.052	0.85	FALSE
121.3	131.7	1.41	1.31	0.052	660	78.4	-0.052	0.56	FALSE
119.9	128.9	1.44	1.33	0.046	229	59.4	-0.046	1.20	FALSE
123.1	138.1	1.48	1.47	0.053	343	76.0	-0.053	1.11	FALSE
120.3	101.7	1.21	1.62	0.039	360	50.3	-0.039	0.51	FALSE
126.4	136.4	1.49	1.47	0.045	204	62.7	-0.045	1.52	FALSE
129.9	141.0	1.51	1.53	0.037	181	56.7	-0.037	1.60	FALSE
128.5	144.4	1.50	1.55	0.041	352	72.1	-0.041	1.07	FALSE
130.8	142.0	1.53	1.55	0.040	120	50.9	-0.040	2.17	FALSE
140.0	160.0	1.27	2.00	0.070	120	80.6	-0.070	3.87	FALSE
140.0	160.0	2.00	1.48	0.030	120	57.1	-0.030	2.74	FALSE
140.0	160.0	1.40	1.87	0.070	120	82.4	-0.070	3.96	FALSE
140.0	160.0	1.53	1.83	0.070	120	83.6	-0.070	4.01	FALSE
140.0	160.0	1.46	1.00	0.030	120	55.2	-0.030	2.65	FALSE
140.0	80.0	2.00	1.00	0.070	900	88.4	-0.070	0.28	FALSE
140.0	160.0	1.78	1.91	0.070	120	85.0	-0.070	4.08	FALSE

140.0	160.0	1.00	2.00	0.030	900	78.4	-0.030	0.50	FALSE
140.0	160.0	1.00	2.00	0.030	120	50.2	-0.030	2.41	FALSE
140.0	160.0	1.12	1.00	0.030	120	52.5	-0.030	2.52	FALSE
140.0	80.0	2.00	1.00	0.070	120	62.8	-0.070	1.51	FALSE
140.0	160.0	1.00	1.00	0.070	120	75.6	-0.070	3.63	FALSE
140.0	132.7	2.00	1.00	0.030	120	50.6	-0.030	2.01	FALSE
140.0	139.2	2.00	2.00	0.030	120	51.9	-0.030	2.17	FALSE

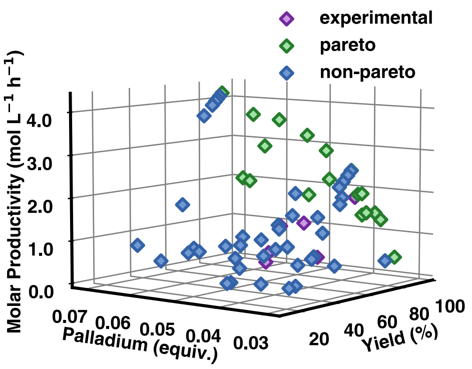

Figure S58. Optimization results case study 1, Pareto points (orange), non-pareto points (green) and experimentally performed points (blue).

8.2. Case Study 2: Rhenium catalyzed thioanisole oxidation

The objectives for this optimization were to maximize the yield, while minimizing perchlorate equivalents and catalyst loading using parameters from model 2 (Table S9).
The boundaries of the variables were defined with:

- temperature: 50 – 90°C
- concentration 4-bromothioanisole (**4**): 80 – 150 mM
- equivalents NBu_4ClO_4: 0.2 – 1.0
- catalyst loading: 0.4 – 1.0 mol%
- residence time: 2 – 15 min

All points of the optimization are shown in Table S15.

Table S15. Results multi-objective optimization for the rhenium catalyzed thioether oxidation. Entries highlighted in green were performed experimentally for model validation.

T (°C)	c(thioanisole) (mM)	equiv. (NBu_4ClO_4)	DP loading (mol%)	t_{res} (s)	yield (%)	DP result	Productivity	Pareto Optimal
84.3	106.2	0.31	0.93	190	39.4	-0.31	0.79	TRUE
90.0	128.1	0.36	0.70	349	56.9	-0.36	0.75	TRUE
89.0	125.6	0.69	0.73	532	97.7	-0.69	0.83	TRUE
90.0	119.8	0.45	0.80	610	86.1	-0.45	0.61	TRUE
90.0	134.8	0.50	0.85	175	55.8	-0.50	1.56	TRUE
90.0	140.4	0.66	0.89	137	59.8	-0.66	2.22	TRUE
90.0	141.0	0.67	0.93	393	96.8	-0.67	1.25	TRUE
90.0	147.0	0.37	1.00	272	61.7	-0.37	1.21	TRUE
90.0	135.7	0.74	0.95	551	100.0	-0.74	0.89	TRUE
90.0	146.0	0.61	0.98	251	78.1	-0.61	1.63	TRUE
90.0	146.7	0.45	0.97	120	48.2	-0.45	2.12	TRUE
90.0	147.6	0.24	1.00	120	34.4	-0.24	1.52	TRUE
90.0	150.0	0.72	1.00	120	62.8	-0.72	2.82	TRUE
90.0	142.1	0.33	0.97	482	70.2	-0.33	0.75	TRUE
90.0	150.0	0.77	1.00	222	85.3	-0.77	2.07	TRUE
90.0	150.0	0.83	0.97	396	100.0	-0.83	1.36	TRUE
90.0	139.2	0.54	0.94	452	91.5	-0.54	1.02	TRUE
90.0	150.0	0.56	1.00	120	55.3	-0.56	2.49	TRUE
90.0	132.9	0.20	1.00	352	46.7	-0.20	0.63	TRUE
90.0	129.4	0.78	0.88	498	100.0	-0.78	0.94	TRUE
90.0	150.0	0.85	1.00	120	68.6	-0.85	3.09	TRUE
90.0	150.0	0.82	1.00	174	79.3	-0.82	2.48	TRUE
90.0	150.0	0.41	0.95	446	77.6	-0.41	0.94	TRUE
90.0	150.0	0.20	0.73	575	48.9	-0.20	0.46	TRUE

90.0	150.0	0.34	1.00	120	42.0	-0.34	1.89	TRUE
90.0	150.0	0.54	1.00	203	67.4	-0.54	1.79	TRUE
82.6	150.0	0.20	1.00	120	26.1	-0.20	1.17	TRUE
90.0	148.7	0.80	0.96	501	100.0	-0.80	1.07	TRUE
90.0	150.0	0.20	1.00	900	62.2	-0.20	0.37	TRUE
90.0	150.0	0.64	1.00	120	59.1	-0.64	2.66	TRUE
68.9	128.7	0.73	0.62	772	60.5	-0.73	0.36	FALSE
72.4	148.9	0.97	0.79	464	70.8	-0.97	0.82	FALSE
51.3	106.5	0.22	0.52	876	14.1	-0.22	0.06	FALSE
65.8	106.5	0.85	0.48	387	35.2	-0.85	0.35	FALSE
62.7	103.1	0.65	0.55	286	24.4	-0.65	0.32	FALSE
76.8	89.6	0.79	0.68	850	87.5	-0.79	0.33	FALSE
74.7	140.8	0.52	0.42	157	23.6	-0.52	0.76	FALSE
63.0	140.1	0.71	0.74	345	35.7	-0.71	0.52	FALSE
79.5	111.4	0.79	0.42	629	66.8	-0.79	0.43	FALSE
62.5	119.7	0.86	0.41	735	39.6	-0.86	0.23	FALSE
52.7	125.2	0.91	0.84	151	12.3	-0.91	0.37	FALSE
57.4	81.8	0.28	0.53	389	13.1	-0.28	0.10	FALSE
51.7	80.1	0.87	0.43	475	11.6	-0.87	0.07	FALSE
89.8	130.1	0.95	0.45	288	72.3	-0.95	1.18	FALSE
69.8	123.1	0.67	0.92	477	58.5	-0.67	0.54	FALSE
75.4	127.8	0.56	0.82	674	70.0	-0.56	0.48	FALSE
70.4	143.5	0.28	0.94	428	36.7	-0.28	0.44	FALSE
70.7	122.6	0.84	0.58	631	60.5	-0.84	0.42	FALSE
68.4	104.4	0.78	0.83	235	39.9	-0.78	0.64	FALSE
90.0	131.2	0.60	0.60	265	62.9	-0.60	1.12	FALSE
90.0	133.8	0.76	0.68	237	72.1	-0.76	1.46	FALSE
90.0	117.0	0.22	0.89	680	58.6	-0.22	0.36	FALSE
90.0	150.0	0.20	0.64	900	54.1	-0.20	0.32	FALSE
90.0	80.0	0.20	0.54	900	50.3	-0.20	0.16	FALSE
81.2	107.5	0.20	0.67	618	39.5	-0.20	0.25	FALSE
90.0	120.7	0.79	0.75	706	100.0	-0.79	0.62	FALSE
90.0	80.0	0.20	1.00	900	61.5	-0.20	0.20	FALSE
90.0	80.0	0.20	0.90	535	50.7	-0.20	0.27	FALSE

69.6	80.0	0.20	1.00	900	39.9	-0.20	0.13	FALSE
81.0	150.0	0.20	0.86	900	50.3	-0.20	0.30	FALSE

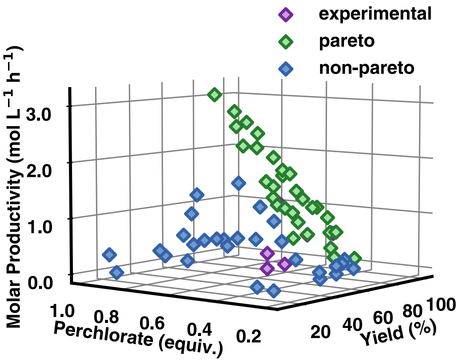

Figure S59. Optimization results case study 2, Pareto points (orange), non-pareto points (green) and experimentally performed points (blue).

8.3. Case Study 3: Copper Catalyzed meta-selective Coupling

The objectives for this optimization were to maximize yield, while minimizing equivalents of mesityl(phenyl)iodonium triflate (**7**) and catalyst loading.
The boundaries of the variables were defined with:

- temperature: 60 – 140°C
- concentration *N*-(*o*-tolyl)pivalamide (**6**): 50 – 85 mM
- equivalents mesityl(phenyl)iodonium triflate (**7**): 1.0 – 3.0
- catalyst loading: 1 – 60 mol%
- residence time: 2 – 15 min

All points of the optimization are shown in Table S16.

Table S16. Results multi-objective optimization for the copper catalyzed meta-selective coupling reaction. Entries highlighted in green were performed experimentally for model validation.

T (°C)	c(amide) (mM)	equiv. (diarl)	Cu loading (mol%)	t_{res} (s)	yield (%)	diarl result	Cu result	Pareto Optimal
104.3	82.4	1.54	44.9	410	45.7	-1.54	-0.45	TRUE
121.4	75.4	1.51	2.9	550	16.2	-1.51	-0.03	TRUE
103.9	70.9	1.30	41.6	607	39.5	-1.30	-0.42	TRUE
104.4	59.4	2.85	58.3	774	87.5	-2.85	-0.58	TRUE
106.4	79.6	1.00	2.0	851	10.5	-1.00	-0.02	TRUE
106.0	72.2	2.02	29.7	605	58.6	-2.02	-0.30	TRUE
106.1	74.8	1.91	12.2	648	44.7	-1.91	-0.12	TRUE
103.8	72.1	1.74	1.0	696	8.6	-1.74	-0.01	TRUE
97.9	85.0	1.00	27.9	900	31.4	-1.00	-0.28	TRUE
107.4	81.9	1.68	21.0	730	48.5	-1.68	-0.21	TRUE
105.8	73.8	2.29	36.7	707	68.8	-2.29	-0.37	TRUE
112.8	85.0	1.22	8.6	900	30.9	-1.22	-0.09	TRUE
107.0	78.8	2.21	19.0	732	62.5	-2.21	-0.19	TRUE
122.7	85.0	1.00	1.0	900	5.9	-1.00	-0.01	TRUE
111.7	85.0	1.90	1.0	900	16.9	-1.90	-0.01	TRUE
110.5	85.0	1.00	17.3	900	28.3	-1.00	-0.17	TRUE
106.0	78.4	2.49	31.9	685	74.4	-2.49	-0.32	TRUE
111.2	81.9	1.42	16.3	818	39.3	-1.42	-0.16	TRUE
97.3	85.0	1.00	54.5	900	33.4	-1.00	-0.54	TRUE
111.3	85.0	1.66	5.1	781	33.8	-1.66	-0.05	TRUE
108.8	85.0	1.87	15.8	667	51.3	-1.87	-0.16	TRUE
112.5	85.0	1.14	1.0	683	8.4	-1.14	-0.01	TRUE
107.1	85.0	1.82	27.1	802	54.1	-1.82	-0.27	TRUE
104.8	83.8	2.66	25.5	769	79.8	-2.66	-0.25	TRUE

107.4	85.0	2.40	8.2	628	50.4	-2.40	-0.08	TRUE
105.3	85.0	2.45	16.7	731	68.2	-2.45	-0.17	TRUE
101.1	85.0	2.11	25.4	895	65.3	-2.11	-0.25	TRUE
109.4	85.0	1.00	6.1	900	22.9	-1.00	-0.06	TRUE
101.6	85.0	1.47	23.9	900	45.2	-1.47	-0.24	TRUE
113.2	66.4	1.60	45.4	258	42.6	-1.60	-0.45	FALSE
112.6	69.8	1.01	43.9	570	28.2	-1.01	-0.44	FALSE
91.8	81.4	2.63	59.9	258	57.6	-2.63	-0.60	FALSE
132.5	60.8	2.34	51.2	374	34.2	-2.34	-0.51	FALSE
133.0	53.6	1.55	13.4	873	9.6	-1.55	-0.13	FALSE
137.8	74.6	1.69	15.0	418	7.7	-1.69	-0.15	FALSE
77.0	55.9	1.40	41.6	569	15.6	-1.40	-0.42	FALSE
115.7	73.8	1.10	36.4	685	29.1	-1.10	-0.36	FALSE
81.6	63.7	1.82	40.0	584	28.8	-1.82	-0.40	FALSE
82.9	65.0	1.57	15.4	439	10.5	-1.57	-0.15	FALSE
130.6	56.4	1.72	53.9	503	28.4	-1.72	-0.54	FALSE
130.3	52.2	2.19	1.8	774	3.9	-2.19	-0.02	FALSE
136.7	77.1	2.60	18.3	696	16.5	-2.60	-0.18	FALSE
135.0	64.8	1.13	23.6	264	9.2	-1.13	-0.24	FALSE
129.3	69.4	2.85	46.5	195	51.4	-2.85	-0.46	FALSE
126.0	69.7	1.28	20.2	652	22.4	-1.28	-0.20	FALSE
60.0	85.0	1.00	1.0	900	3.5	-1.00	-0.01	FALSE
60.0	85.0	1.00	1.0	120	0.5	-1.00	-0.01	FALSE
91.1	85.0	1.00	13.6	532	14.0	-1.00	-0.14	FALSE
110.3	85.0	1.00	1.0	120	1.4	-1.00	-0.01	FALSE
60.0	50.0	1.00	1.0	900	2.1	-1.00	-0.01	FALSE
115.3	50.0	1.00	1.0	120	1.0	-1.00	-0.01	FALSE
60.0	50.0	1.00	1.0	120	0.3	-1.00	-0.01	FALSE
104.5	50.0	1.00	34.1	900	29.8	-1.00	-0.34	FALSE
103.0	50.0	1.00	60.0	900	31.2	-1.00	-0.60	FALSE
109.1	85.0	2.13	1.0	511	11.0	-2.13	-0.01	FALSE
60.0	67.7	1.00	1.0	900	2.8	-1.00	-0.01	FALSE
114.1	50.0	1.00	1.0	900	5.9	-1.00	-0.01	FALSE
102.5	50.0	1.00	43.5	120	12.0	-1.00	-0.44	FALSE

119.7	59.7	1.00	1.0	531	4.5	-1.00	-0.01	FALSE
60.0	85.0	1.00	51.9	120	1.6	-1.00	-0.52	FALSE

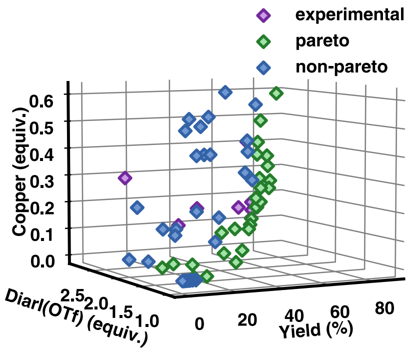

Figure S60. Optimization results case study 2, Pareto points (orange), non-pareto points (green) and experimentally performed points (blue).

9. Model Validation

Steady state experiments within the experimental space as well as Pareto points of previous *in silico* optimization (Section 0) were performed for model validation. Each steady state was held for at least three constant UHPLC measurements. The experimental results were compared with simulated results using the previously fitted models.

9.1. Case Study 1: Buchwald Hartwig Reaction

Steady state experiments from Table S17 show very good alignment of the model predicted values with the experimental data with a RMSE of 11.1 mM for the product concentration. These results are visualized in the parity plots (Figure S61), where **2** shows the largest deviations from the model prediction. This error can result from the additional decomposition pathway fitted for **2**.

Table S17. Parameters selected for steady state experiments for the Buchwald Hartwig reaction. Measured and predicted product (**3**) concentration together with a deviation are given.

	t_{res} (s)	T (°C)	c(1) (mM)	equiv. 2	equiv. DBU	Pd loading (mol%)	Predicted 3 (mM)	Measured 3 (mM)	Deviation (mM)
1	404	120	102	1.24	1.54	5.0	64.1	68.5	4.5
2	166	135	116	1.5	1.1	4.0	66.1	57.6	-8.5
3	300	125	120	1.1	1.6	3.5	61.6	61.1	-0.5
4	360	105	110	1.4	1.2	4.5	52.9	53.2	0.2
5	342	123	138	1.48	1.47	5.2	104.0	121.6	17.6
6*	247	140	160	1.46	1.41	3.0	115.0	98.1	-16.9
7*	291	140	160	1.63	1.37	3.0	120.7	103.8	-16.9
8*	208	140	149	1.56	1.36	3.0	99.0	89.2	-9.7
9*	200**	140	151**	1.74	1.78	3.47	110.2	116.6	6.4

*Pareto point

**residence time and concentration reduced from optimized pareto point

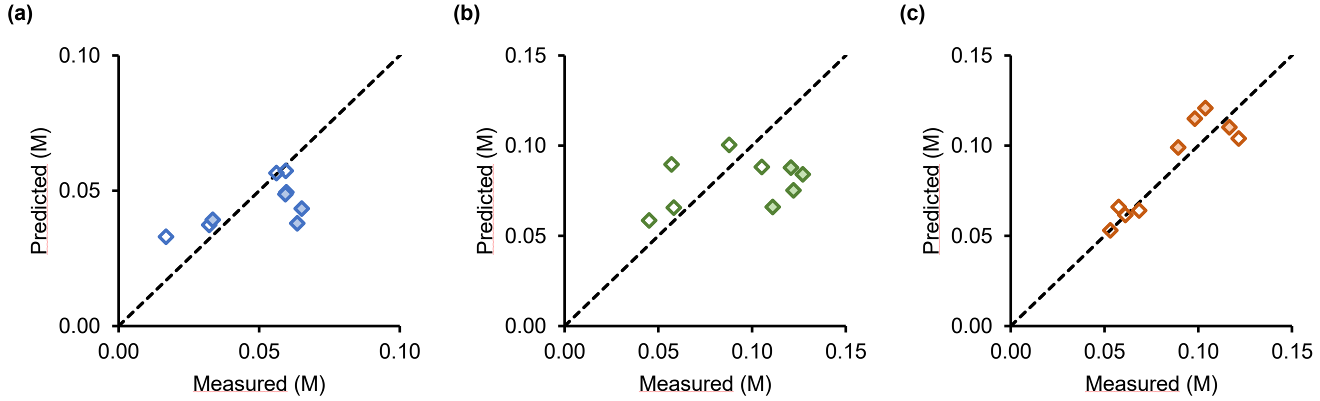

Figure S61. Parity Plots for validation experiments of the Buchwald Hartwig reaction. Filled data points represent Pareto points. (a) 2-bromonitrobenzene (**1**), (b) 2-amino-5-methyl-3-thiophenecarbonitrile (**2**), (c) product (**3**).

9.1. Case Study 2: Rhenium catalyzed thioanisole oxidation

Steady state experiments given in Table S18 show excellent alignment of the model predicted values with the experimental data with a RMSE of 7.2 mM for the product concentration. These results are visualized in the parity plots (Figure S62).

Table S18. Parameters selected for steady state experiments for the rhenium catalyzed thioanisole oxidation. Measured and predicted product (**5**) concentration together with a deviation are given.

	t_{res} (s)	T (°C)	c(4) (mM)	equiv. NBu_4ClO_4	DP loading (mol%)	Predicted 5 (mM)	Measured 5 (mM)	Deviation (mM)
1	500	75	100	0.50	0.7	52.1	48.0	-4.1
2	350	80	110	0.30	0.5	36.4	34.6	-1.8
3	700	60	120	0.40	0.9	44.6	45.5	0.8
4	203	90	100	0.54	1.0	66.5	58.2	-8.3
5*	349	90	128	0.36	0.69	72.3	66.4	-6.0
6*	610	90	119	0.45	0.8	102.6	86.3	-16.2
7*	575	90	150	0.20	0.72	73.2	76.5	3.3
8*	456**	90	134**	0.33	0.97	92.2	88.9	-3.3

*Pareto point

**residence time and concentration reduced from optimized pareto point

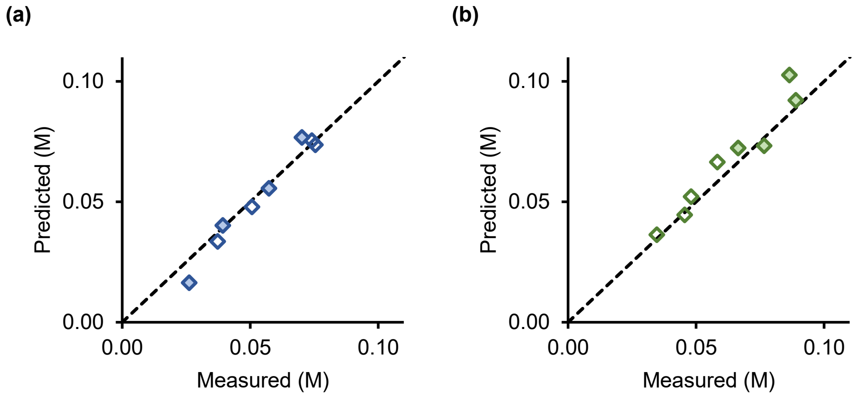

Figure S62. Parity Plots for validation experiments of the Buchwald Hartwig reaction. Filled data points represent Pareto points. (a) **4**, (b) **5**.

9.1. Case Study 3: Copper Catalyzed meta-selective Coupling

Steady state experiments from Table S19 show excellent alignment of the model predicted values with the experimental data with a RMSE of 2.2 mM for the product concentration. These results are visualized in the parity plots (Figure S63).

Table S19. Parameters selected for steady state experiments for the copper catalyzed meta-selective coupling reaction. Measured and predicted product (**8**) concentration together with a deviation are given.

	t_{res} (s)	T (°C)	c(6) (mM)	equiv. (7)	Cu loading (mol%)	Predicted 8 (mM)	Measured 8 (mM)	Deviation (mM)
1	600	105	85	1.5	21	35.8	39.7	3.9
2	360	90	65	1.7	16	9.5	11.4	1.9
3	249	100	80	2.0	20	23.8	25.9	2.1
4	252	113	65	1.6	45	27.5	24.1	-3.4
5	430	70	45	2.5	30	4.0	4.9	0.8
6*	732	107	79	2.2	19	49.1	50.6	1.5
7*	671**	105	85	2.45	17	50.6	50.7	0.1
8*	900	101	85	1.47	24	35.0	33.2	-1.8
9*	667	108	85	1.87	16	43.4	45.3	1.8

*Pareto point

**residence time and concentration reduced from optimized pareto point

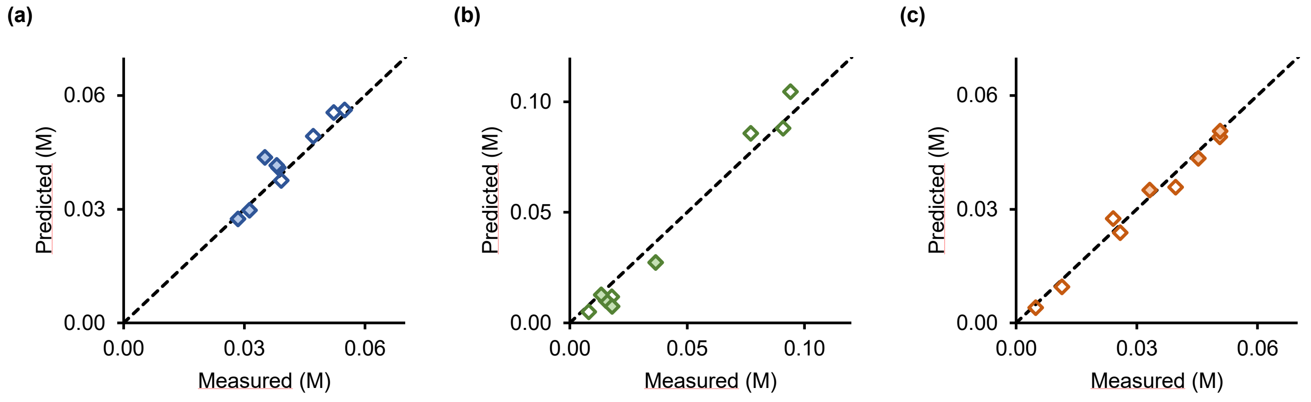

Figure S63. Parity Plots for validation experiments of the Cu catalyzed meta-selective coupling reaction. Filled data points represent Pareto points. (a) **6**, (b) **7**, (c) **8**.

10. Product Synthesis

10.1. Case Study 1: Synthesis of Buchwald Hartwig Products

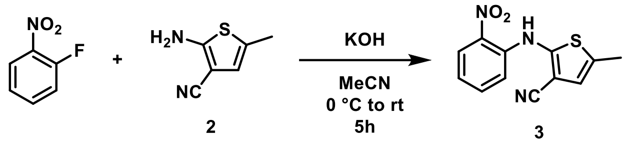

Scheme S7. Synthesis of Buchwald Hartwig product (**3**) for calibration.

A 50 mL round bottom flask was charged with acetonitrile (20 mL). 2-amino-5-methyl-3-thiophenecarbonitrile (**2**) (1.00 g, 7.24 mmol, 1.0 equiv.) and 2-fluoronitrobenzene (1.22 g, 8.68 mmol, 1.2 equiv.) were added. The reaction mixture was cooled to 0 °C and a suspension of potassium hydroxide (1.01 g, 18.0 mmol, 2.5 equiv.) in acetonitrile (5 mL) was added. The reaction mixture was warmed to room temperature. After 5 h it was filtered and the solvent was removed under reduced pressure. The product was recrystallized from methanol, crashed out with water and isolated *via* filtration. After drying in the vacuum oven for 24 h at 40 °C 0.68 g (36 %) of **3** were isolated as orange solid.
^{1}H NMR (300 MHz, Chloroform-*d*) δ 9.61 (s, 1H), 8.25 (dd, *J* = 8.5, 1.6 Hz, 1H), 7.52 (ddd, *J* = 8.7, 7.0, 1.6 Hz, 1H), 7.19 (dd, *J* = 8.6, 1.3 Hz, 1H), 6.96 (ddd, *J* = 8.5, 7.1, 1.3 Hz, 1H), 6.78 (t, *J* = 1.2 Hz, 1H), 2.63 – 2.28 (m, 6H).
^{13}C NMR (75 MHz, $CDCl_3$) δ 149.06, 141.40, 136.35, 136.31, 134.26, 126.80, 124.08, 120.03, 116.24, 113.82, 104.84, 77.58, 77.16, 76.74, 15.76.

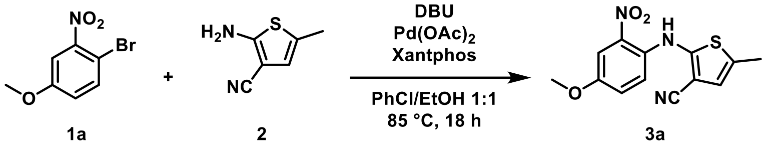

Scheme S8. Synthesis of methoxy substituted Buchwald Hartwig product (**3a**) for calibration.

A 50 mL round bottom flask was charged with $Pd(OAc)_2$ (56 mg, 0.25 mmol, 0.05 equiv.), Xantphos (217 mg, 0.375 mmol, 0.075 equiv.) and set under argon atmosphere. A mixture of phenylchloride and ethanol 1:1 (10 mL) was added as a solvent. A solution of 1-bromo-4-methoxy-2-nitrobenzene (**1a**) (1.16 g, 5.0 mmol, 1.0 equiv.) and 2-amino-5-methyl-3-thiophenecarbonitrile (**2**) (760 mg, 5.5 mmol, 1.1 equiv.) in pheylchloride/ethanol (10 mL) and DBU (0.91 g, 6.0 mmol, 1.2 equiv.) was added and the reaction mixture was degassed and heated to 85 °C for 18 h. The solvent was removed under reduced pressure. The reaction mixture was resuspended in dichloromethane and filtered over a silica plug. The product was recrystallized from methanol, crashed out with water and isolated *via* filtration. After drying under reduced pressure, 780 mg (54 %) of **3a** were isolated as dark red solid.
^{1}H NMR (300 MHz, Chloroform-*d*) δ 9.46 (s, 1H), 7.66 (d, *J* = 2.8 Hz, 1H), 7.22 (d, *J* = 9.1 Hz, 1H), 7.16 (dd, *J* = 9.3, 2.8 Hz, 1H), 6.72 (d, *J* = 1.2 Hz, 1H), 3.83 (s, 3H), 2.44 (d, *J* = 1.0 Hz, 3H).
^{13}C NMR (75 MHz, $CDCl_3$) δ 153.01, 150.34, 135.44, 134.71, 134.32, 125.63, 123.76, 117.96, 114.04, 108.06, 102.79, 56.07, 15.63.

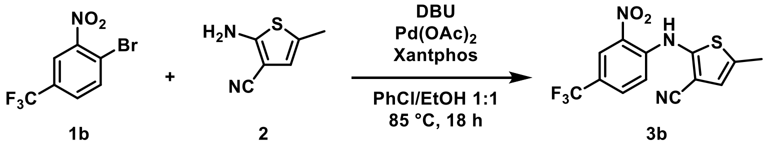

Scheme S9. Synthesis of trifluoromethyl substituted Buchwald Hartwig product (**3b**) for calibration.

A 50 mL round bottom flask was charged with $Pd(OAc)_2$ (56 mg, 0.25 mmol, 0.05 equiv.), Xantphos (217 mg, 0.375 mmol, 0.075 equiv.) and set under argon atmosphere. A mixture of phenylchloride and ethanol 1:1 (10 mL) was added as a solvent. A solution of 1-bromo-2-nitro-4-(trifluoromethyl)benzene (**1b**) (1.35 g, 5.0 mmol, 1.0 equiv.) and 2-amino-5-methyl-3-thiophenecarbonitrile (**2**) (760 mg, 5.5 mmol, 1.1 equiv.) in pheylchloride/ethanol (10 mL) and DBU (0.91 g, 6.0 mmol, 1.2 equiv.) was added and the reaction mixture was degassed and heated to 85 °C for 18 h. The solvent was removed under reduced pressure. The reaction mixture was resuspended in dichloromethane and filtered over a silica plug. The product was recrystallized from methanol, crashed out with water and isolated *via* filtration. After drying under reduced pressure, 402 mg (25%) of **3b** were isolated as yellow solid.
^{1}H NMR (300 MHz, Chloroform-*d*) δ 9.68 (s, 1H), 8.55 (s, 1H), 7.70 (dd, *J* = 8.9, 2.1 Hz, 1H), 7.19 (d, *J* = 8.9 Hz, 1H), 6.85 (d, *J* = 1.2 Hz, 1H), 2.51 (s, 3H).
^{13}C NMR (75 MHz, $CDCl_3$) δ 160.69, 146.86, 144.05, 138.53, 133.19, 132.58, 132.54, 124.80, 124.61, 116.82, 113.29, 107.17, 15.87.
^{19}F NMR (282 MHz, $CDCl_3$) δ -62.28.

10.2. Case Study 3: Synthesis of Starting Materials and Product of Copper Catalyzed meta-selective Coupling

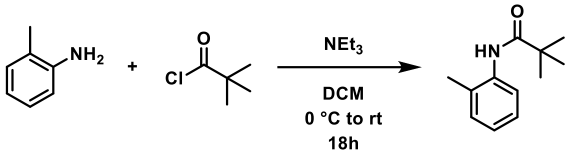

6

Scheme S10. Synthesis of *N*-(*o*-tolyl)pivalamide (**6**).

A 250 mL round bottom flask was charged with dichloromethane (120 mL), o-toluidine (7.50 g, 7.5 mL, 70 mmol, 1.0 equiv.) and triethylamine (10.6 g, 14.6 mL, 105 mmol, 1.5 equiv.). The reaction mixture was cooled to 0 °C and trimethylacetylchloride (10.1 g, 10.3 mL, 84 mmol, 1.2 equiv.) was added slowly. The reaction mixture was warmed to room temperature and stirring was continued for 18 h. The reaction mixture was extracted with H_2O (100 mL), then HCl (1 mol/L, 100 mL), then H_2O (100 mL), dried over Na_2SO_4, filtered and the solvent was removed under reduced pressure to obtain 11.5 g (92%) of **6** as off-white solid.[10]

^{1}H NMR (300 MHz, Chloroform-*d*) δ 7.80 (d, *J* = 8.1 Hz, 1H), 7.30 (s, 1H), 7.23 – 7.12 (m, 2H), 7.11 – 7.01 (m, 1H), 2.23 (s, 3H), 1.33 (s, 9H).

^{13}C NMR (75 MHz, $CDCl_3$) δ 176.54, 135.93, 130.39, 129.09, 126.77, 124.98, 123.10, 39.71, 27.74, 17.66.

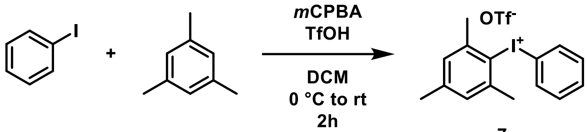

7

Scheme S11. Synthesis of mesityl(phenyl)iodonium triflate (**7**).

A 250 mL round bottom flask was charged with dichloromethane (120 mL), mCPBA (7.36 g, 27.8 mmol, 1.11 equiv.), iodobenzene (5.10 g, 4.0 mL, 25 mmol, 1.0 equiv.), mesitylene (3.33 g, 3.8 mL, 27.8 mmol, 1.11 equiv.) and cooled to 0 °C. Trifluoromethanesulfonic acid (4.58 g, 2.7 mL, 30.5 mmol, 1.22 equiv.) was added slowly. The reaction mixture was warmed to room temperature over 2 h and the solvent of the light brown reaction mixture was removed under reduced pressure. The reaction mixture was suspended in diethyl ether (100 mL) and the solvent was evaporated again. The reaction mixture was suspended in diethyl ether (100 mL), sonicated and cooled to 0 °C. The product was isolated *via* filtration and dried under reduced pressure to obtain 8.05 g (68%) of **7** as colorless solid.

^{1}H NMR (300 MHz, Chloroform-*d*) δ 7.57 – 7.48 (m, 1H), 7.69 (dd, *J* = 8.5, 1.1 Hz, 1H), 7.45 – 7.36 (m, 1H), 7.10 (s, 1H), 2.62 (s, 3H), 2.34 (s, 1H).

^{13}C NMR (75 MHz, $CDCl_3$) δ 144.57, 142.62, 133.11, 132.37, 130.48, 122.51, 120.47, 118.27, 111.85, 27.22, 21.23.

^{19}F NMR (282 MHz, $CDCl_3$) δ -78.33.

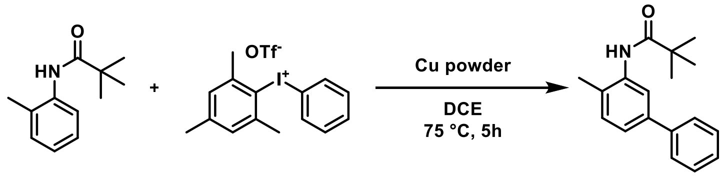

6 7 8

Scheme S12. Synthesis of copper catalyzed coupling product (**8**).

A 50 mL round bottom flask was charged with *N*-(*o*-tolyl)pivalamide (**6**) (143 mg, 0.75 mmol, 1.0 equiv.), mesityl(phenyl)iodonium triflate (**7**) (708 mg, 1.5 mmol, 2.0 equiv.) and copper powder (285 mg, 4.5 mmol, 6.0 equiv.) and flushed with argon. Dichloroethane (15 mL) was added as solvent. The reaction mixture was heated to 75 °C and stirring was continued for 5 h. The reaction mixture was filter *via* a syringe filter, diluted with dichloromethane (20 mL) and washed with saturated $NaHCO_3$ solution (2 x 20 mL) and H_2O (20 mL). The aqueous phases were backwashed with DCM (10 mL) and the combined organic phases were dried over Na_2SO_4, filtered and the solvent was evaporated under reduced pressure. The product was further purified by column chromatography (DCM/cyclohexane 70:30) to isolate 136 mg (68 %) of **8** as colourless solid.[11]

^{1}H NMR (300 MHz, Chloroform-*d*) δ 8.24 (d, *J* = 1.7 Hz, 1H), 7.64 (dd, *J* = 8.3, 1.3 Hz, 2H), 7.48 – 7.40 (m, 2H), 7.38 – 7.31 (m, 3H), 7.29 (s, 1H), 2.33 (s, 3H), 1.40 (s, 9H).

^{13}C NMR (75 MHz, $CDCl_3$) δ 176.68, 140.80, 140.12, 136.36, 130.87, 128.77, 127.53, 127.31, 127.24, 123.50, 121.46, 39.97, 27.88, 17.46.

11. NMR Spectra

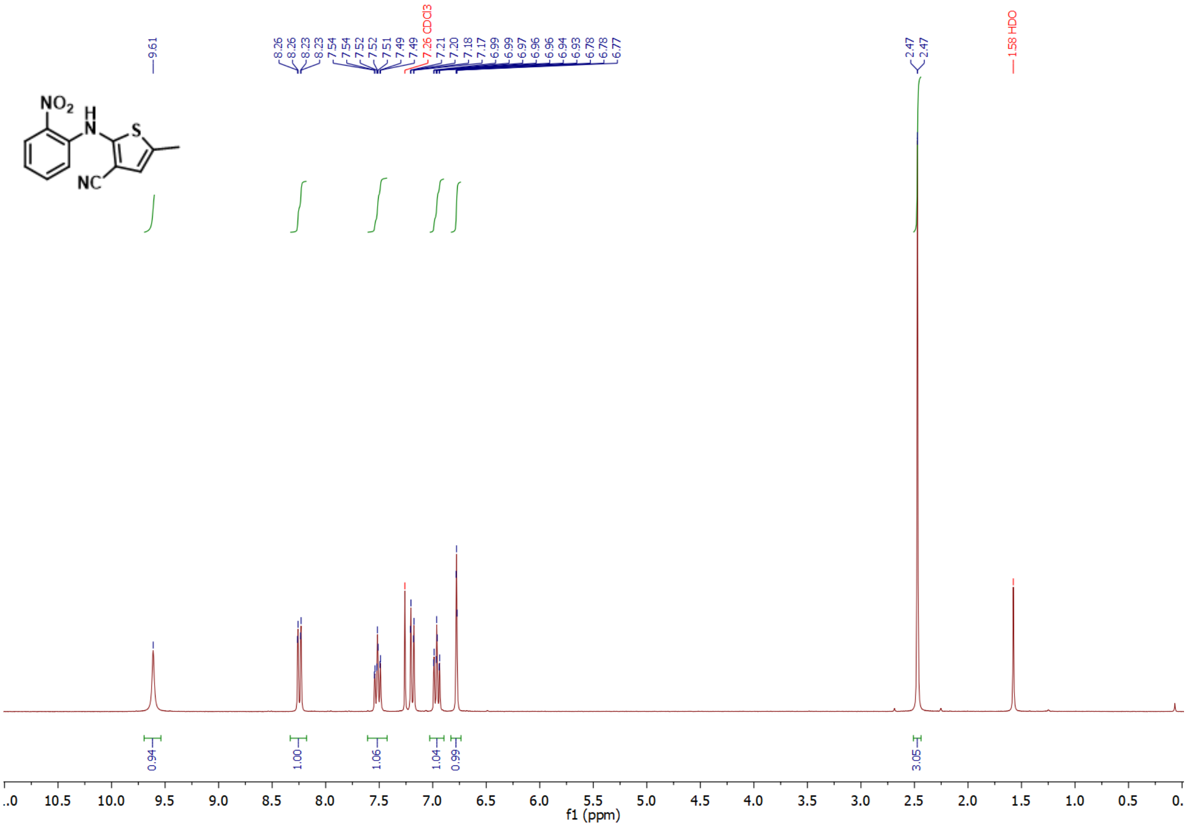

Figure S64. ^{1}H NMR spectrum of **3**.

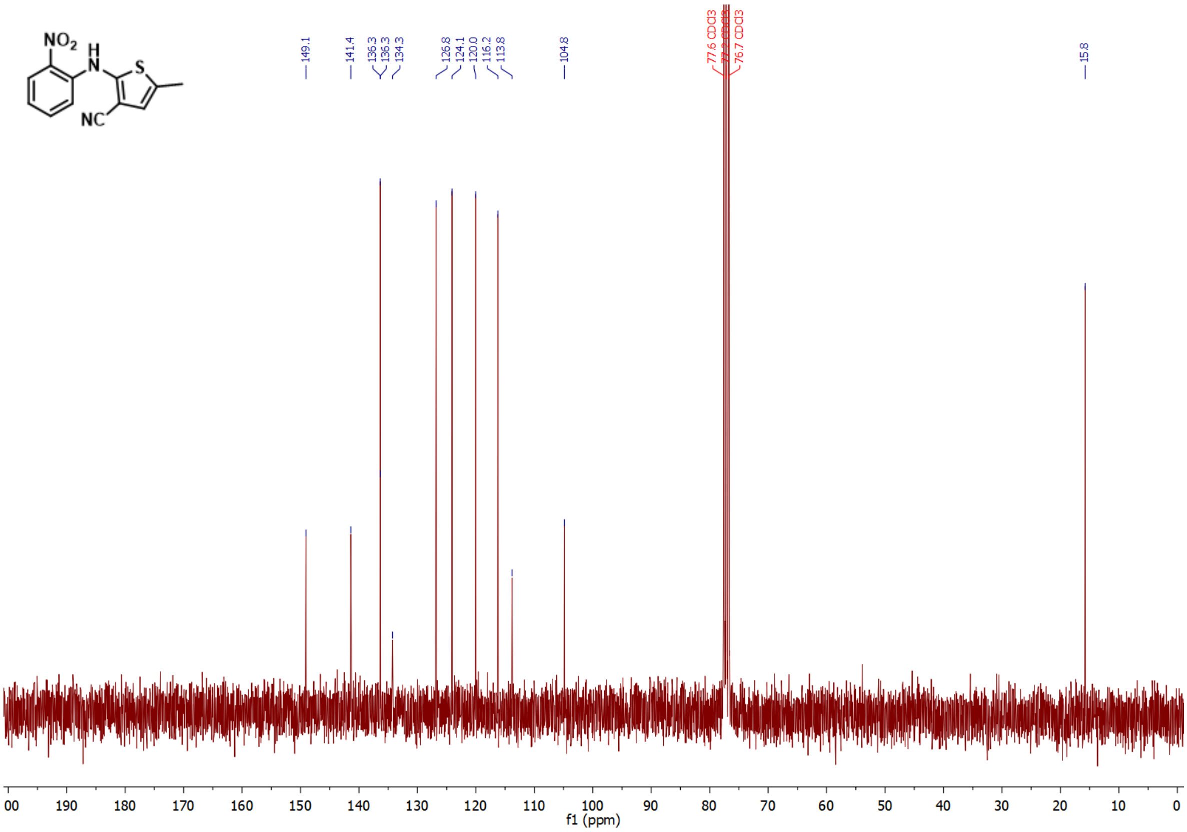

Figure S65. ^{13}C NMR spectrum of **3**.

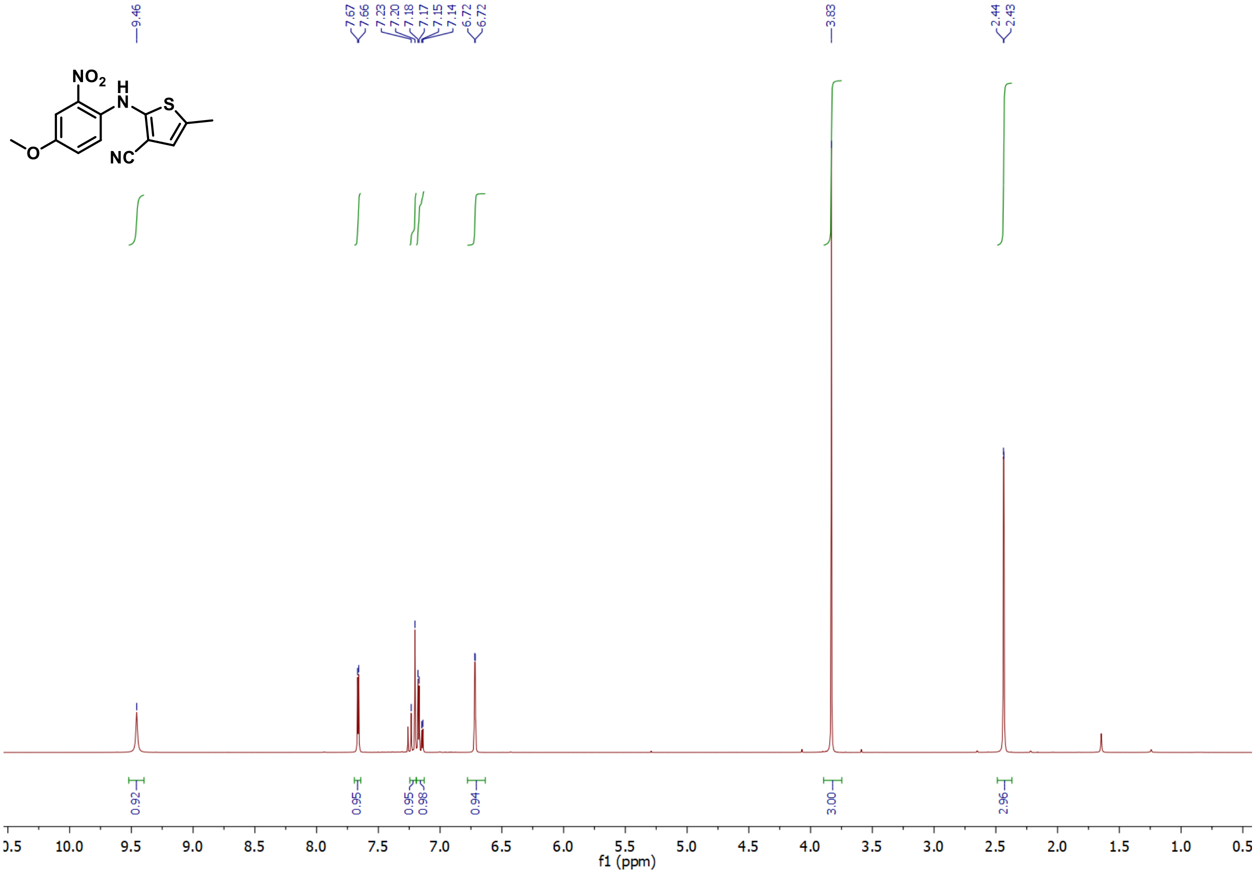

Figure S66. ^{1}H NMR spectrum of **3a**.

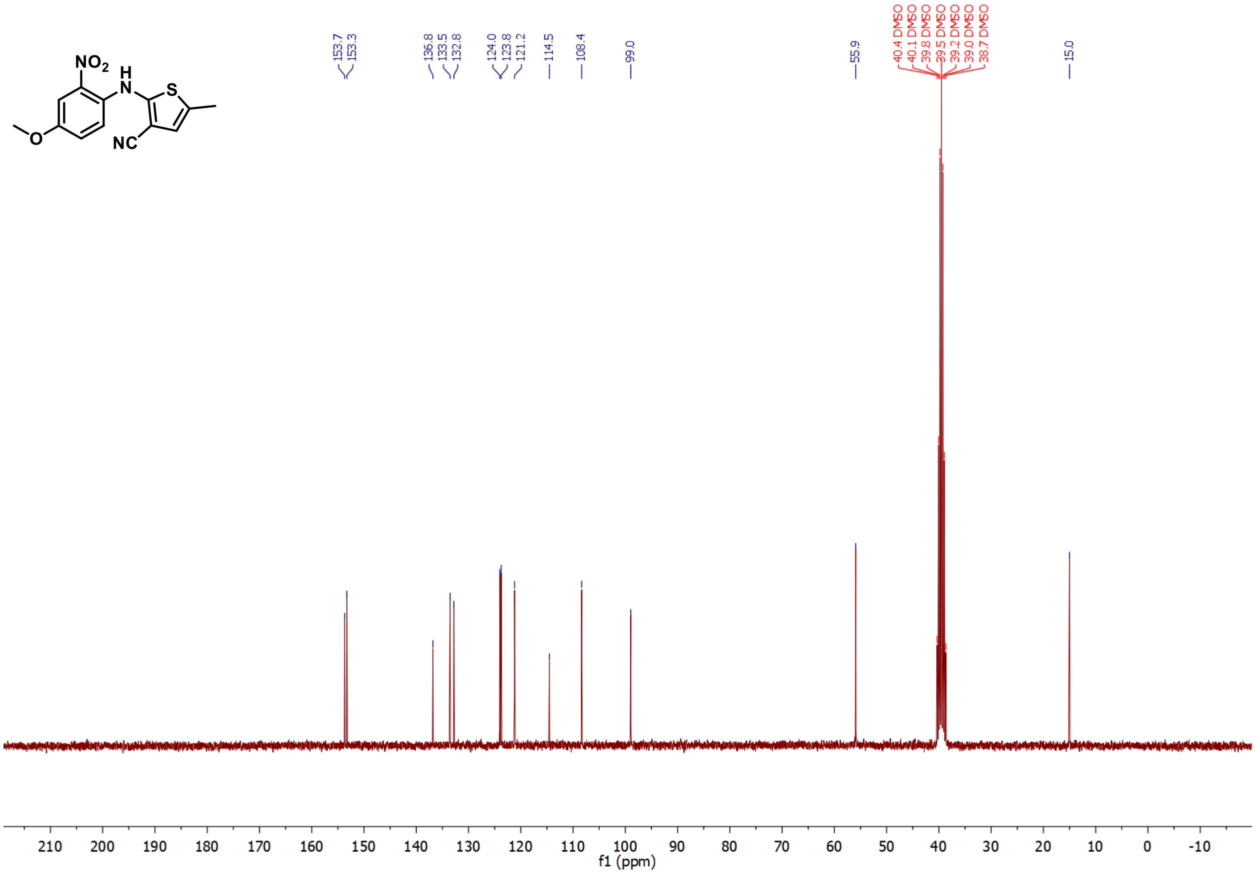

Figure S67. ^{13}C NMR spectrum of **3a**.

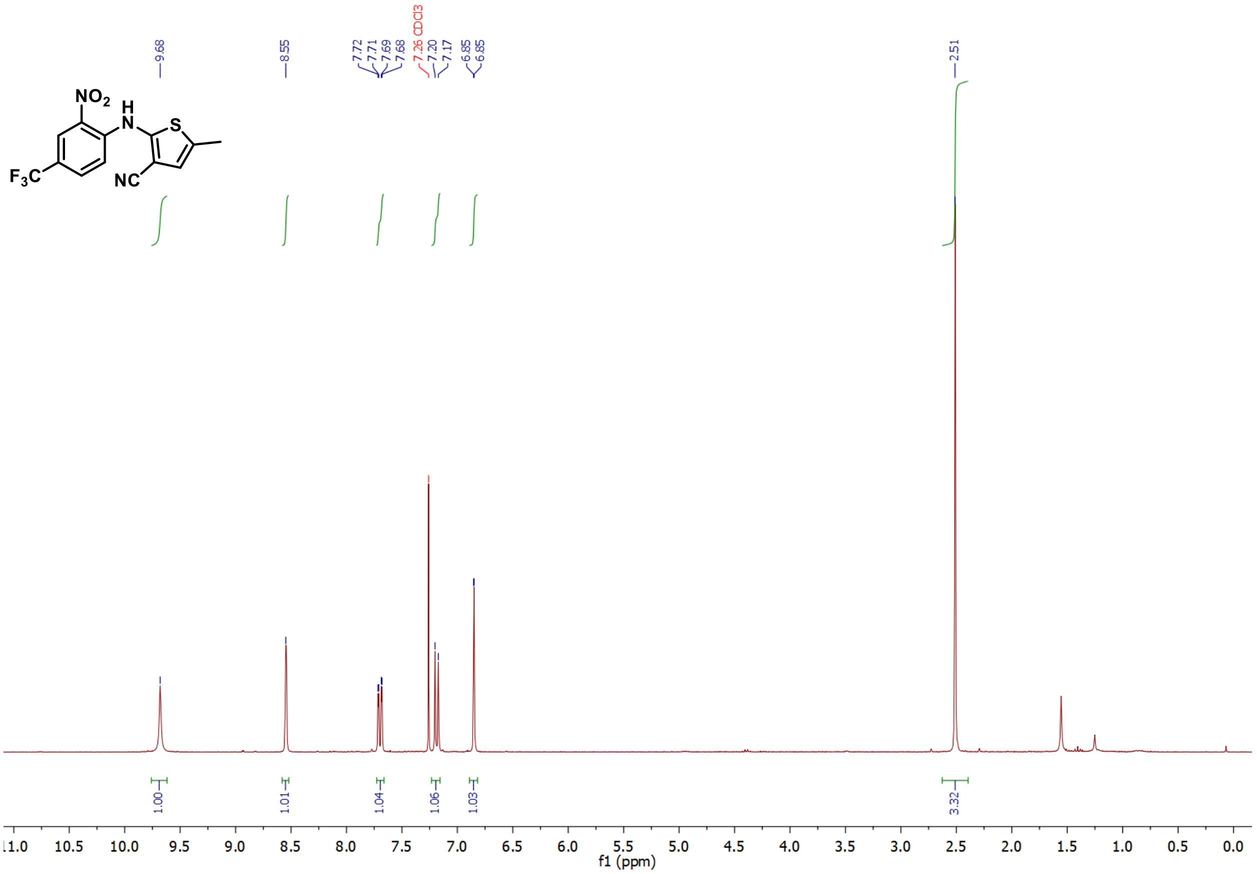

Figure S68. ^{1}H NMR spectrum of **3b**.

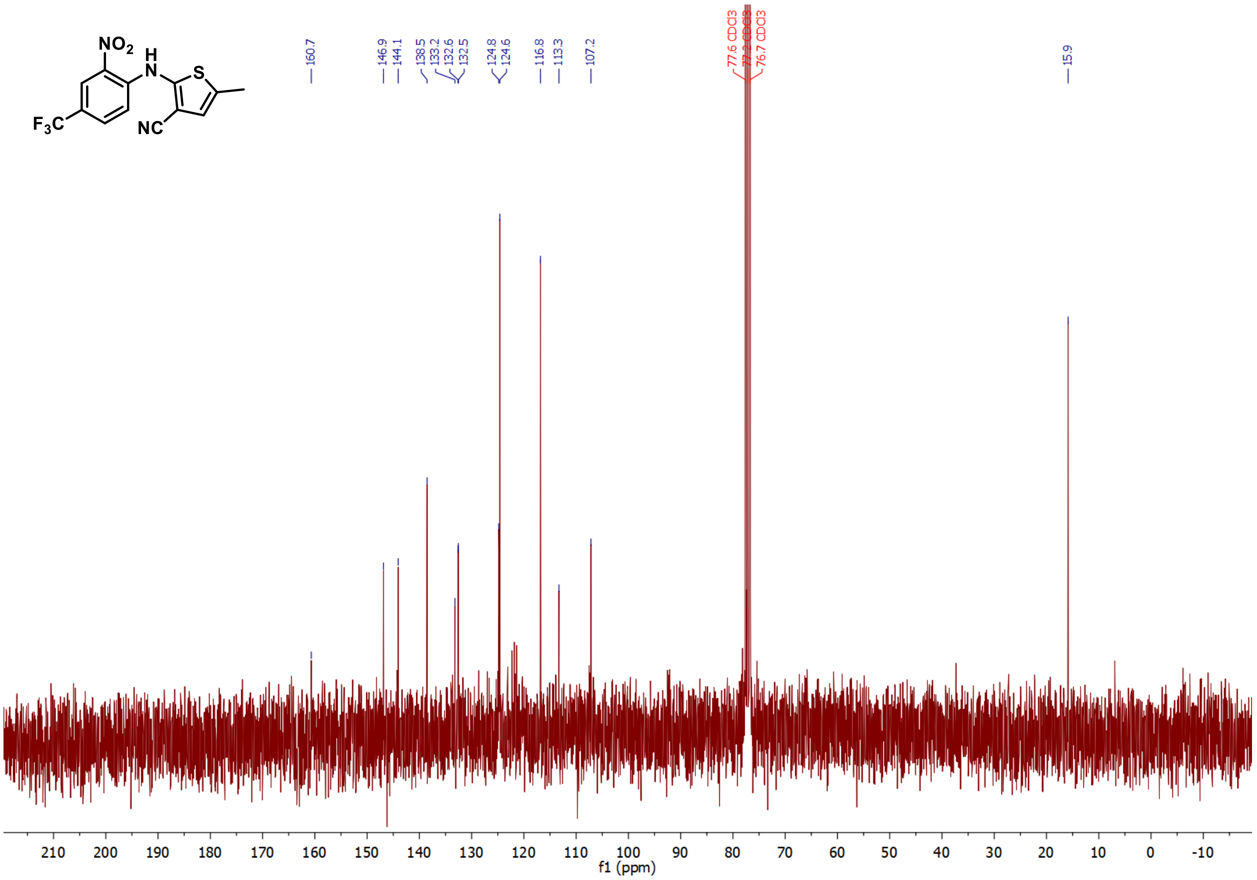

Figure S69. ^{13}C NMR spectrum of **3b**.

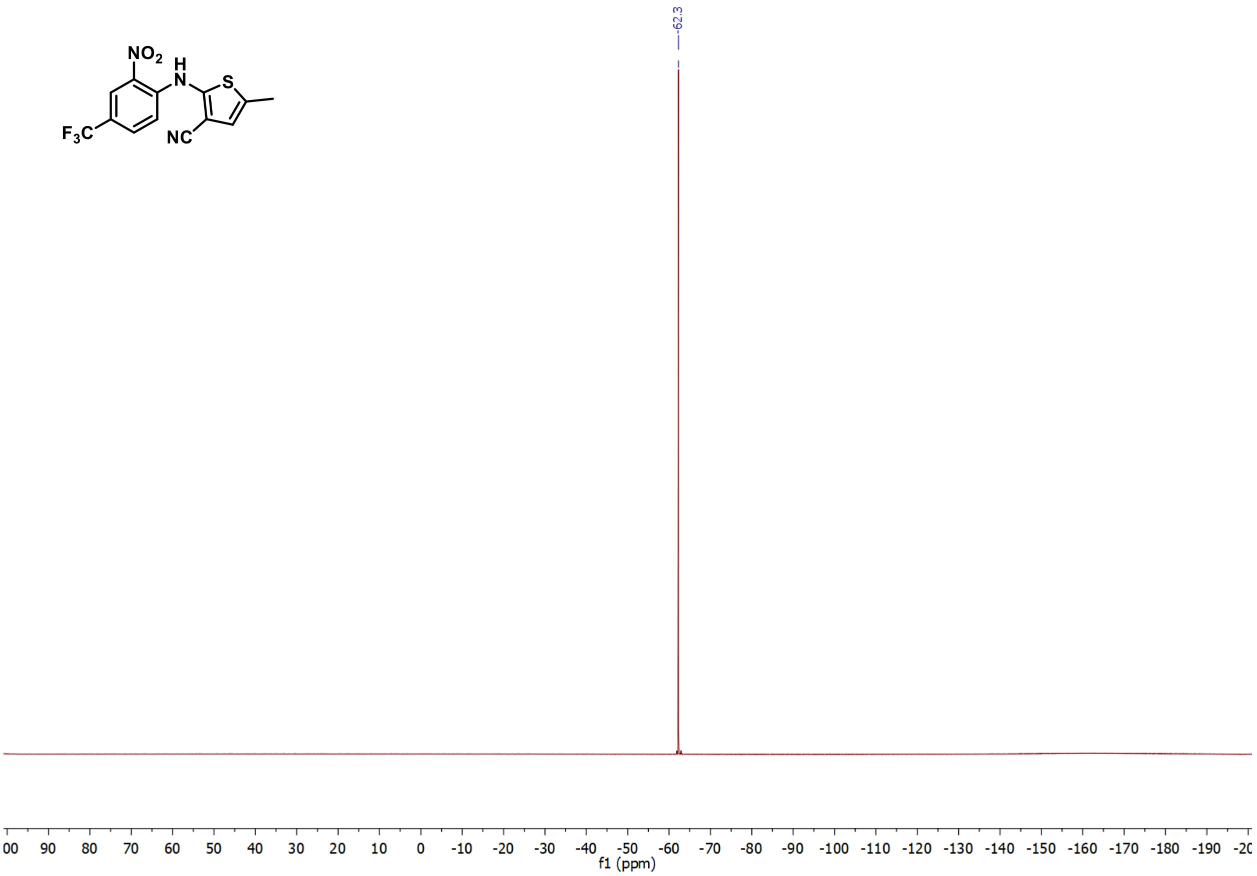

Figure S70. ^{19}F NMR spectrum of **3b**.

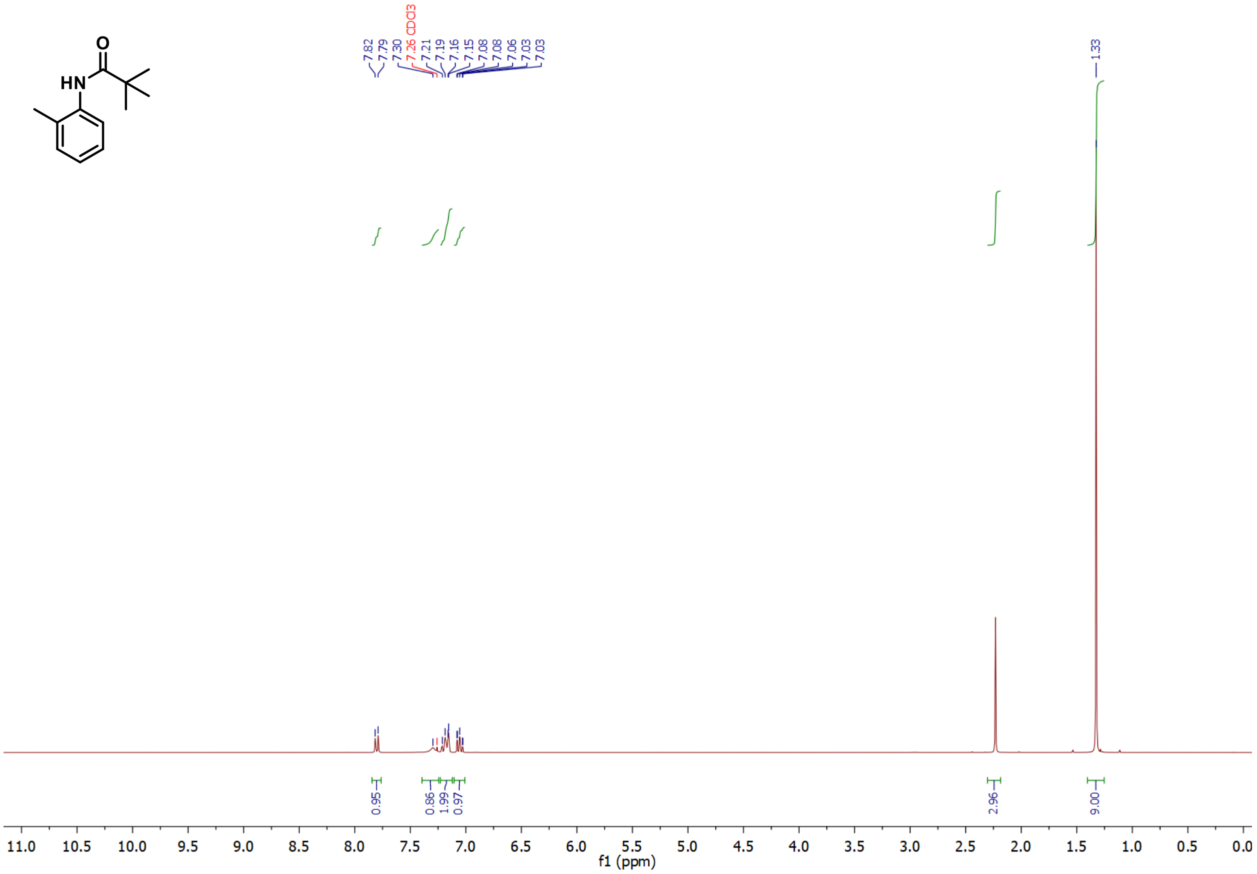

Figure S71. ^{1}H NMR spectrum of **6**.

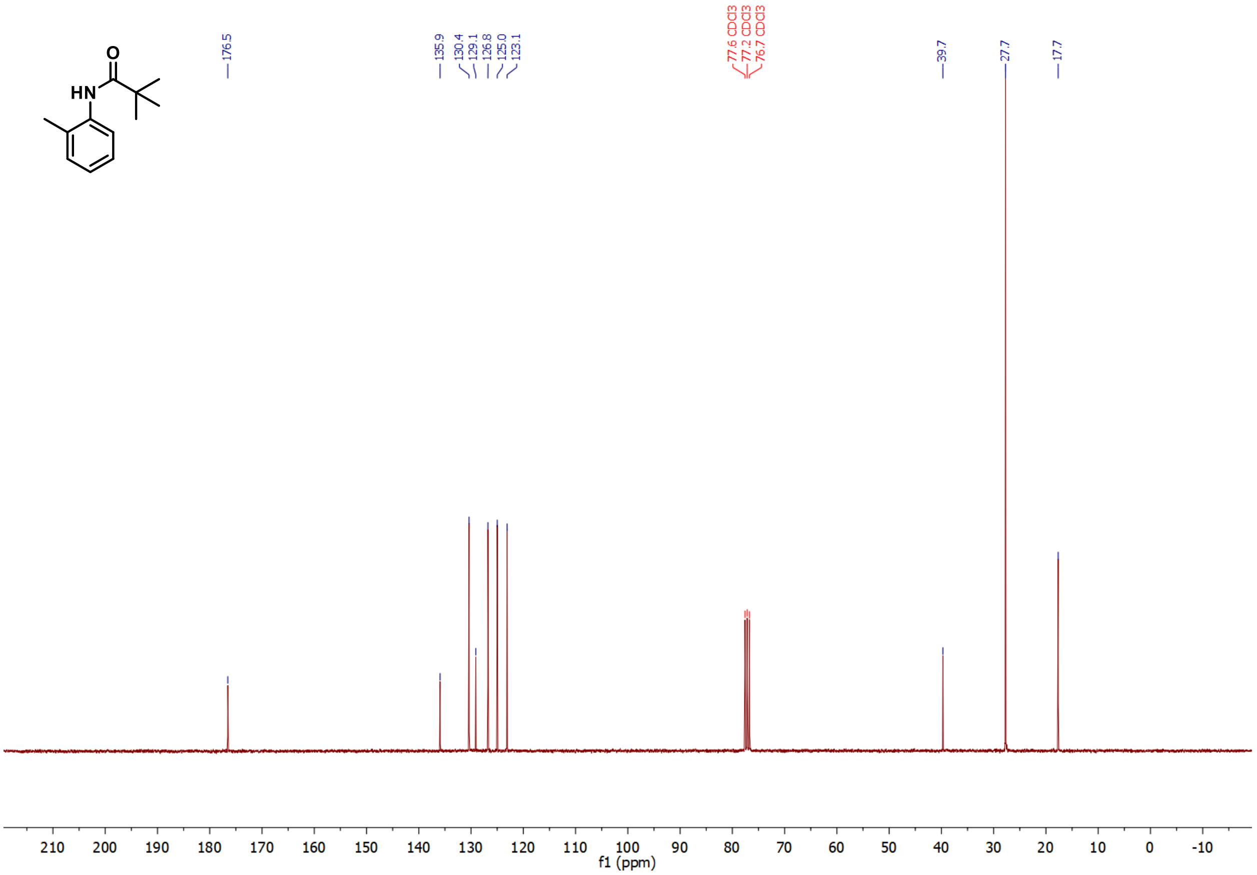

Figure S72. ^{13}C NMR spectrum of **6**.

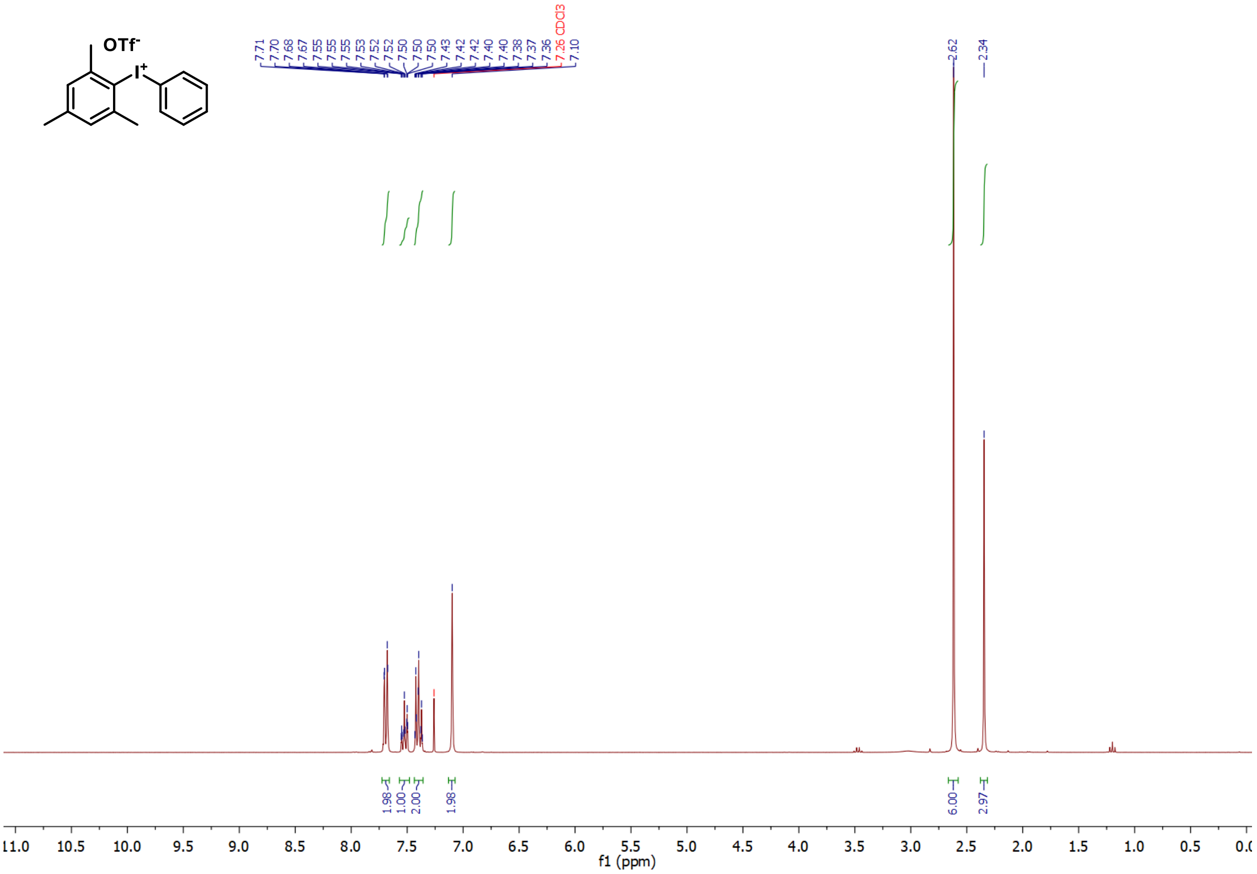

Figure S73. ^{1}H NMR spectrum of **7**.

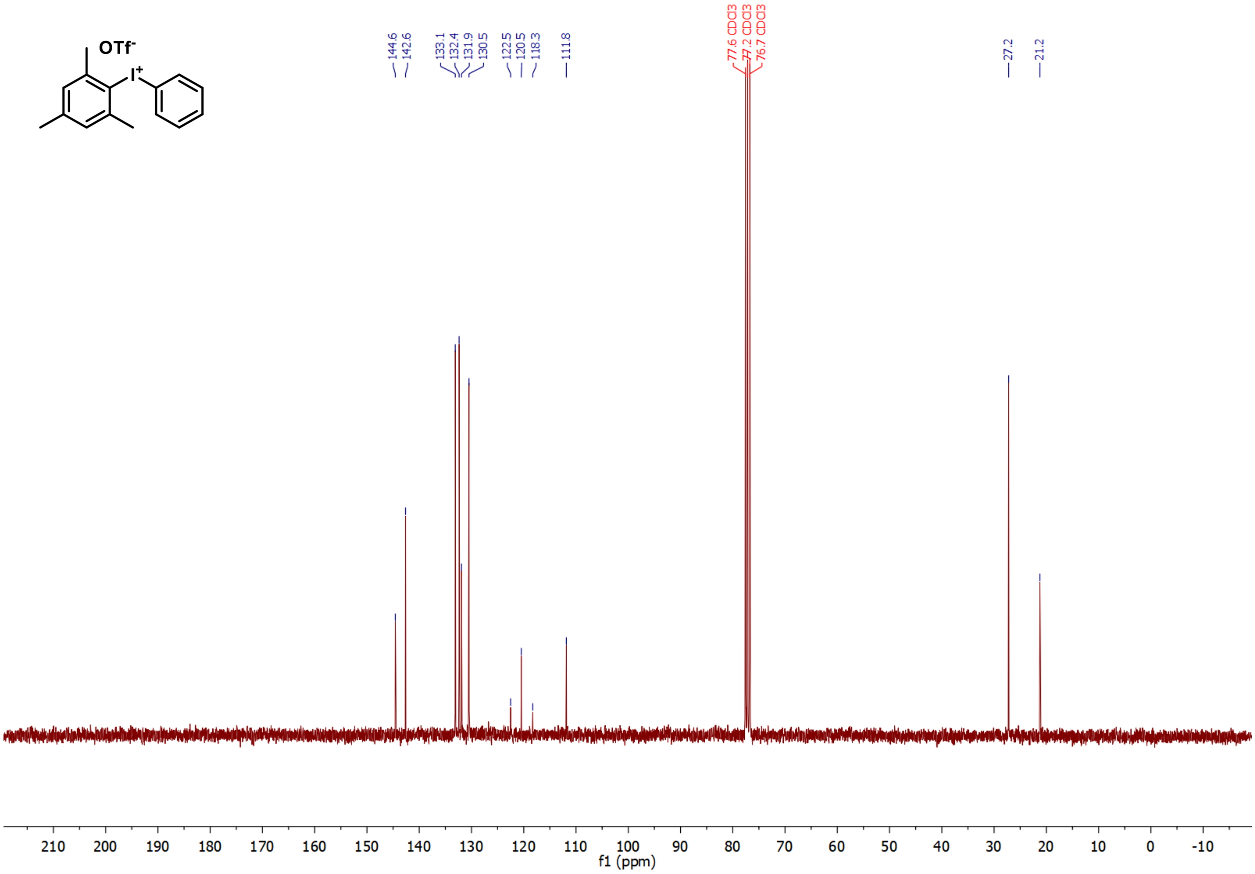

Figure S74. ^{13}C NMR spectrum of **7**.

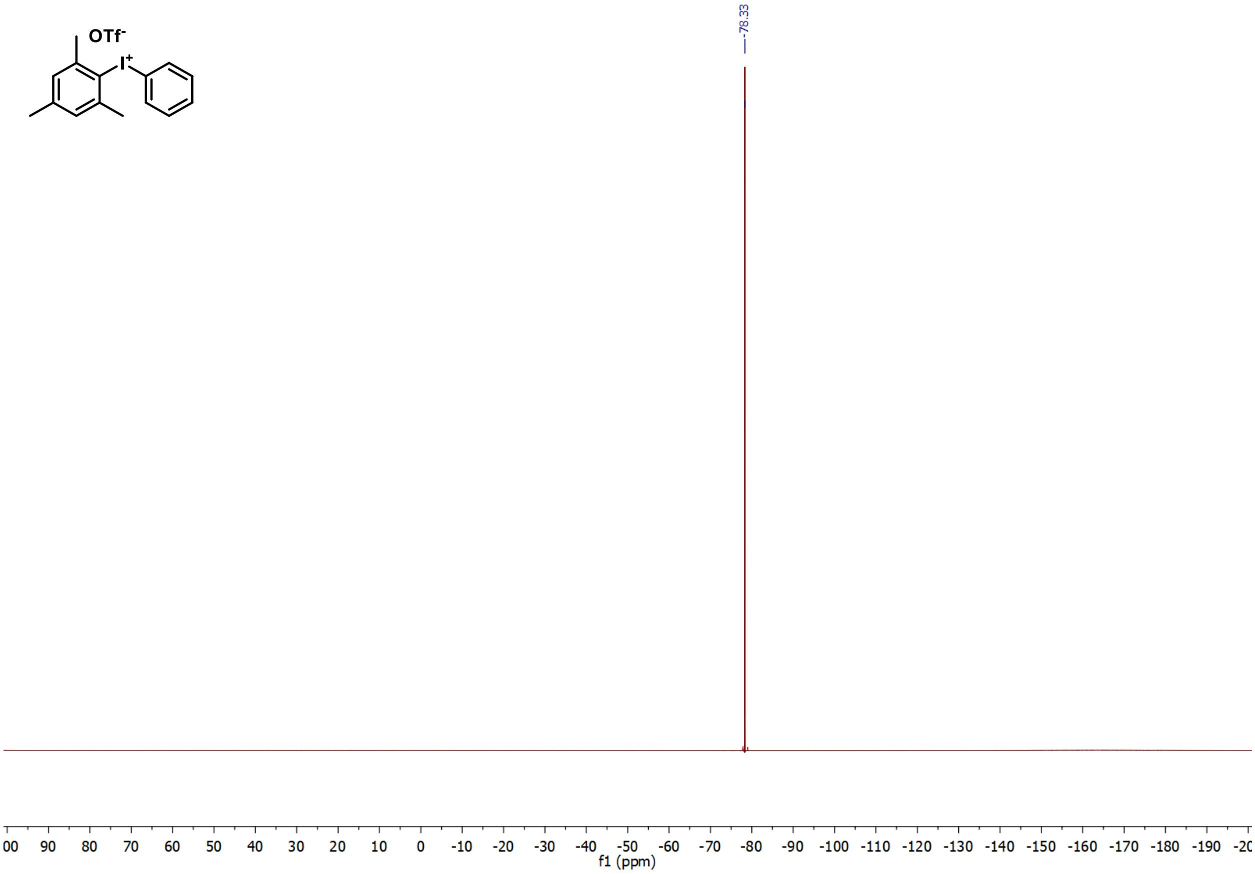

Figure S75. ^{19}F NMR spectrum of **7**.

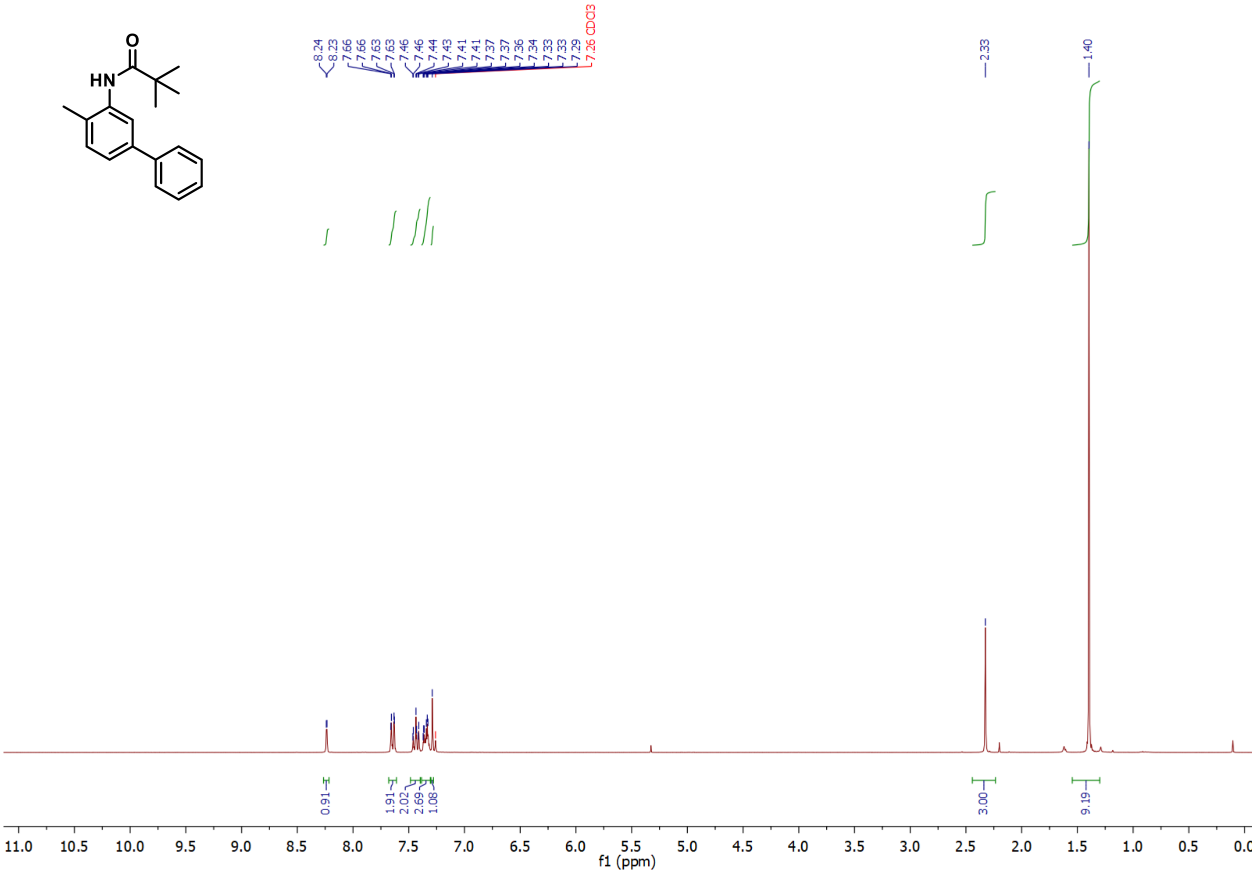

Figure S76. ^{1}H NMR spectrum of **8**.

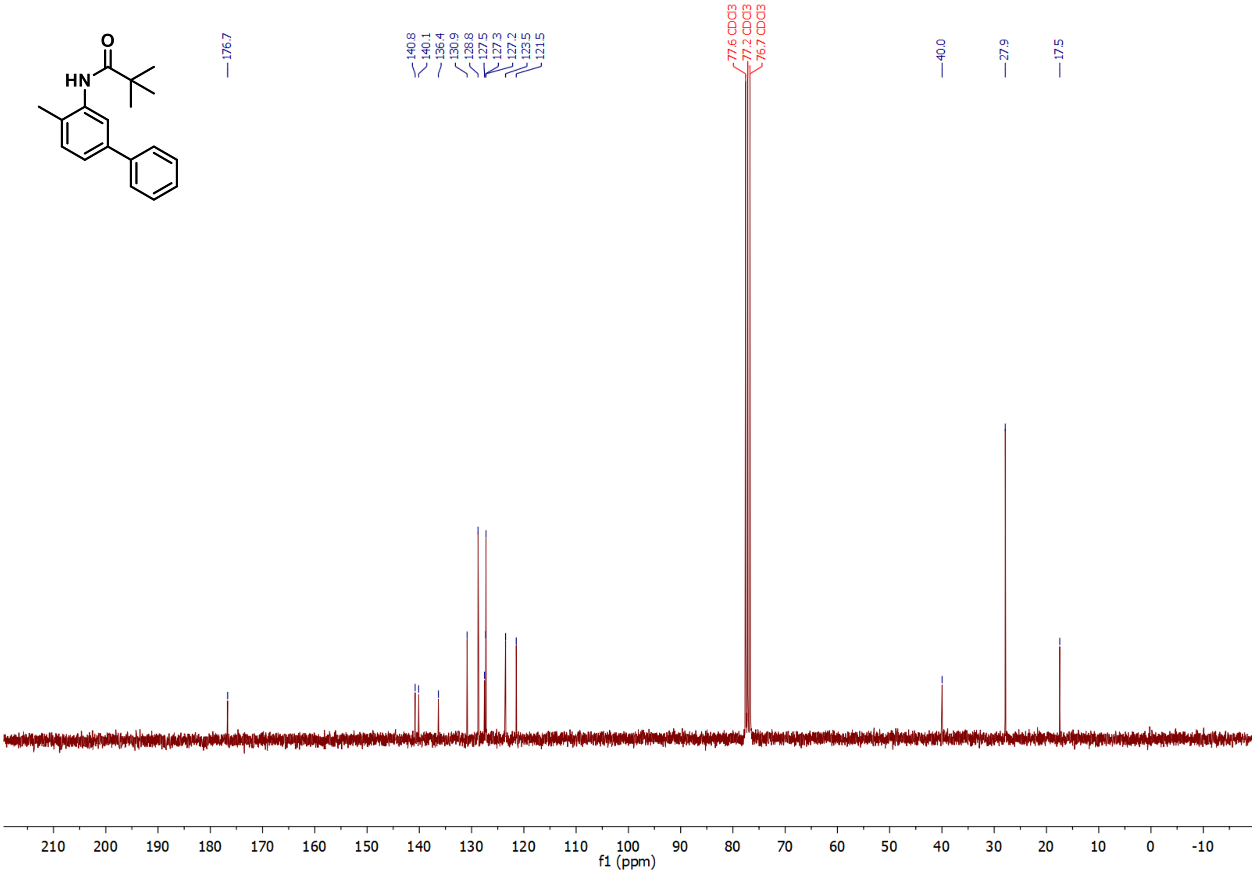

Figure S77. ^{13}C NMR spectrum of **8**.

References

[1] O. Levenspiel, *Chemical Reaction Engineering*, **1999**.

[2] K. Silber, F. L. Wagner, C. A. Hone, C. O. Kappe, *React. Chem. Eng.* **2026**, DOI 10.1039/D5RE00306G.

[3] K. Silber, P. Sagmeister, C. Schiller, J. D. Williams, C. A. Hone, C. O. Kappe, *React. Chem. Eng.* **2023**, *8*, 2849–2855.

[4] C. Waldron, A. Pankajakshan, M. Quaglio, E. Cao, F. Galvanin, A. Gavriilidis, *React. Chem. Eng.* **2020**, *5*, 112–123.

[5] P. A. Forero-Cortés, A. M. Haydl, *Org. Process Res. Dev.* **2019**, *23*, 1478–1483.

[6] A. Begouin, S. Hesse, M.-J. R. P. Queiroz, G. Kirsch, *European J. Org. Chem.* **2007**, *2007*, 1678–1682.

[7] A. J. and A. Mosleh, *Organometallics* **1995**, *14*, 1810–1817.

[8] R. J. Phipps, M. J. Gaunt, *Science (80-.).* **2009**, *323*, 1593–1597.

[9] B. Chen, X.-L. Hou, Y.-X. Li, Y.-D. Wu, *J. Am. Chem. Soc.* **2011**, *133*, 7668–7671.

[10] M. Bielawski, M. Zhu, B. Olofsson, *Adv. Synth. Catal.* **2007**, *349*, 2610–2618.

[11] H. P. L. Gemoets, G. Laudadio, K. Verstraete, V. Hessel, T. Noël, *Angew. Chemie Int. Ed.* **2017**, *56*, 7161–7165.
